# Design and implementation of a multi-country targeted health facility assessment to measure integrated, people-centered, quality of services

**DOI:** 10.1371/journal.pgph.0005736

**Published:** 2026-08-19

**Authors:** Shivam Gupta, Japneet Kaur, Rohit Jain, Alexander K. Rowe, Pankhuri Jha, Rasheed Raji, Arjun Arakal, Chris White, Marasi Mwencha, Shunsuke Mabuchi

**Affiliations:** 1 Johns Hopkins Bloomberg School of Public Health, Baltimore, Maryland, United States of America; 2 The Global Fund to Fight AIDS, Tuberculosis and Malaria, Geneva, Switzerland; 3 The Gates Foundation, Seattle, Washington, United States of America; PLOS: Public Library of Science, UNITED STATES OF AMERICA

## Abstract

Integrated, people-centred, high-quality health services are essential to achieving universal health coverage, yet traditional health facility assessments often focus on structural inputs and are too resource-intensive to support frequent, programmatic use. The Global Fund to Fight AIDS, Tuberculosis (TB), and Malaria developed the Targeted Health Facility Assessment (tHFA) to inform country plans and monitor progress on the Global Fund Strategy (2023–2028) of maximizing people-centred integrated systems for health. This paper describes the development, methodology, and implementation of the tHFA baseline round and discusses issues related to institutionalizing these assessments to monitor and improve service delivery processes and outcomes in low- and middle-income countries (LMICs). The tHFA is a cross-sectional, multi-country facility assessment designed to monitor progress on four key performance indicators in the Global Fund’s strategic monitoring framework and seven programmatic indicators for horizontal program investments. In 2024–2025, the Global Fund implemented a baseline survey across 2,272 facilities in 18 LMICs using a modular questionnaire covering integrated delivery of antenatal care, HIV, TB, and malaria services, supportive supervision, community health worker (CHW) support, and patient-reported experience measures. Probability sampling was used to select 120 health facilities per country, and data were collected through direct observation, health worker interviews, and record reviews. Post-stratification weighting accounted for facility replacement and other sampling deviations, and data quality assurance was embedded throughout via training, pilot testing, and real-time monitoring. The tHFA is a pragmatic, agile, and scalable approach designed to monitor integrated service delivery and people-centred quality of care in LMICs. Its focused scope, inclusion of CHWs, and alignment with the Global Fund’s strategic objectives position it as a tool designed to guide decision-making, inform grant monitoring, and strengthen health systems across LMICs. Its impact on these outcomes will be assessed through planned follow-up rounds of the tHFA over the strategy cycle.

## Introduction

Despite substantial progress in health service accessibility across low- and middle-income countries (LMICs) since 2000, actual health service delivery remains fragmented with quality deficits, resulting in low health system responsiveness and poor user satisfaction [1,2]. In 2015, all United Nations Member States adopted the 2030 Agenda for Sustainable Development, which contains 17 Sustainable Development Goals (SDGs). The broad scope of SDG 3 (“Good Health and Well-Being”)—particularly, Target 3.8 (“Achieve universal health coverage [UHC] by 2030”), requires global health policymakers, donors and program implementers to go beyond service access and improve what happens once people encounter health services [3,4]. This call to improve the process of service delivery was echoed in the World Health Organization’s (WHO’s) World Health Report 2013 and in the subsequent WHO Framework on Integrated, People-Centred Health Services (IPCHS) in May 2016, emphasizing integrated, high-quality, patient-centred services at all levels of care [5,6].

The IPCHS framework emphasizes the transformation of health services to ensure they are accessible, comprehensive, coordinated, and responsive to people’s needs and preferences. The Lancet Global Health Commission on High Quality Health Systems further highlighted that improving health system performance requires health facility assessments (HFAs) that are agile and focus on the process and outcome of care rather than inputs [2]. Concurrently, the Primary Health Care Performance Initiative (PHCPI) contributed significantly to the understanding that achieving quality, sustainable UHC necessitates a shift from vertical programming towards integrated health systems, with integrated service delivery as a key component. The WHO Operational Framework for Primary Health Care formalized this perspective [7].

Since its inception, the Global Fund to Fight AIDS, Tuberculosis (TB) and Malaria (Global Fund) has invested in strengthening the horizontal investments in health systems as an enabler for its disease-focused programming. Realizing the unique opportunity for action presented by the SDGs, and national commitments to UHC, and building on renewed attention on primary health care (PHC) catalysed by the 40th anniversary of Alma-Ata Declaration of 1978 and the Declaration of Astana in 2018 the Global Fund Strategy (2023‒2028) elevated the achievement of “maximizing people-centred, integrated systems for health to deliver impact, resilience, and sustainability” as a key objective across all HIV, TB, and malaria (HTM) investments [8,9]. This strategic shift aims to build on the health systems strengthening investments made in response to the COVID-19 pandemic while contributing to the SDG target of UHC through strengthened health systems. Monitoring Global Fund investments to strengthen health systems via integrated, people-centred quality services (IPCQS) has been operationalized via a framework that includes key performance indicators (KPIs) to measure integrated service delivery, service quality including patient-reported experience measures (PREMs), and support to community health workers (CHWs) [10].

Monitoring the Global Fund’s progress toward IPCQS over the five-year strategy period requires regular data collection on service delivery processes and outcomes at the point of care, using a baseline of 2024–25 to coincide with the signing of new Global Fund country grants, followed by regular rounds of HFAs. A review of available HFA questionnaires, including the Service Availability and Readiness Assessment (SARA), Health Facility Census (HFC), Service Provision Assessment (SPA), and the WHO Harmonized Health Facility Assessment (HHFA) revealed an emphasis on measuring structural readiness and a lack of data elements on people-centred outcomes and support to CHWs [11]. While structural inputs are the necessary foundations for care, they are not sufficient to describe its content or effects; insight into health system quality requires measurement of processes and outcomes of care [12]. SPA and HHFA include modules on process of care and patient satisfaction, however, historically these assessments have required extensive planning and are never conducted in multiple countries simultaneously at regular intervals. Using the available questionnaires as a starting point, the Global Fund designed a Targeted Health Facility Assessment (tHFA) and implemented a baseline round in 18 LMICs in 2024–25. This paper describes the development, methodology and the implementation of the baseline round of the tHFA and discusses key issues related to institutionalizing agile and focused facility assessments to monitor service delivery processes and outcomes to improve accountability for service integration and quality of health services in LMICs.

## Methodology

The baseline round of the tHFA is a cross-sectional, multi-country health facility survey designed to address the persistent gaps in monitoring of health systems strengthening by focusing on the process and outcome of service delivery. Its main objective is to measure 11 quantitative indicators that reflect the Global Fund’s strategic priority of integrating delivery of vertical (e.g., HTM) and horizontal (e.g., workforce, laboratory equipment, supervision) programs at the point of service delivery for an integrated, high-quality experience for the patient. For the purposes of this assessment, eligible health facilities were defined as public sector facilities across all 18 countries, with private sector facilities additionally included in eight countries (Madagascar, Gambia, Liberia, Guinea-Bissau, Mali, Cameroon, Niger, and Nigeria). Eligible facilities were required to offer HTM and/or ANC services and were stratified by level of care: primary (health centres/posts), secondary (district/sub-district hospitals), and tertiary (referral/specialty hospitals). Facilities offering only specialized services (e.g., maternity homes or dental clinics) were excluded.

### Ethics statement

An institutional review board (IRB) in each country reviewed the tHFA questionnaire, the protocol, and consent forms and approved the data collection (S1 Table lists IRB authorities and reference numbers). In Madagascar, Nigeria, Gambia, and Uganda, the country-level IRB determined the assessment to be public health practice and therefore exempted it from human-subjects research. At every facility, the study obtained written consent from the facility in-charge, participating patients, and CHWs prior to interview or observation. In case of minor patients, the written consent was obtained from their parent/guardian. No personally identifiable information was collected from patients, CHWs, or clients included in the interviews or record reviews. All data were recorded in anonymized form, with no names or unique identifiers retained, and client sampling lists were destroyed immediately after record abstraction to ensure confidentiality. The study was conducted in accordance with the approved protocol, with no deviations from the approved study design or data collection procedures.

#### Questionnaire development*.*

The Global Fund developed the tHFA questionnaire by systematically mapping existing questions from the WHO HHFA, filling gaps through a targeted literature review, and consulting with technical experts who participated in developing the strategic monitoring framework for the Global Fund. The group of experts helped with the technical validation and enhanced the specificity of these indicators, aligning them with the frameworks from the WHO and the World Bank that emphasize service delivery integration and quality of care. The consultation also included several Global Fund Secretariat teams including country teams for the 18 countries, three disease (HTM) teams, cross-cutting thematic teams (laboratory, human resources, community rights and gender, supply chain, and pandemic preparedness and response), the ministries of health of these countries, and their national disease programs. A distinctive feature of the tHFA is the inclusion of questions from WHO developed PREMs [13] and support for CHWs within the assessment questionnaire, both of which are Global Fund strategic priorities.

The baseline round was carried out in 2024–25 across 18 countries: Burkina Faso, Cameroon, Chad, Congo, Democratic Republic of the Congo (DRC), Gambia, Ghana, Guinea-Bissau, Liberia, Madagascar, Malawi, Mali, Mozambique, Niger, Nigeria, Tanzania, Senegal and Uganda. The tHFA measures 11 quantitative composite indicators, including four KPIs (Table 1: Section 1) and seven programmatic indicators (Table 1: Section 2) [10,14]. The seven programmatic indicators monitor progress on the Global Fund’s investment priorities at the facility level to strengthen horizontal programs of human resources, laboratory diagnostics, functional oxygen availability, community-led monitoring, and availability of disease guidelines and treatment completion rates.

**Table 1:** Components and attributes of the 11 indicators measured through Targeted Health Facility Assessment (tHFA).

Indicator	Component	Attributes
**Section 1: Key Program Indicators (KPIs)**		
**KPI S1: Provision of integrated, people-centred, high-quality services**	1. Recorded Treatment and Counselling	• ART record review ◦ ART clients with TB screening ◦ ART clients received TB treatment ◦ ART clients with BP recorded • TB record review ◦ TB clients with HIV status recorded ◦ Changes in TB symptoms recorded • ANC record review ◦ HIV testing ◦ ART for HIV+ women ◦ IPTp ◦ TB screening ◦ Syphilis testing ◦ Gestation age recorded ◦ Blood pressure recorded ◦ IFA Provision ◦ Pregnancy danger signs counselling ◦ Treatment for intestinal worms ◦ Hemoglobin testing
	2. Patient Centeredness	• Communication • Respect • Autonomy • Confidentiality • Social support • Informed about diagnosis • Danger signs discussed • Respectful treatment by health workers • Access related challenges
**KPI S2: Provision of integrated supportive supervision**	1. Supportive supervision occurred in last quarter by an external supervisor (e.g., district health officer or senior MoH staff) covering integrated HTM technical content	
	2. Covered technical content	• Data review • Observe clinical and lab functions • Give feedback • On-spot training • Community health activities • Answer questions from health workers
	3. Summary statistics	• Any health area from the following sources: patient or lab registers, commodity stock records, community data or community activities, or other facility data.
	4. Group problem solving	
	5. Community activity data	• This could include a simple discussion about which community activities had occurred in the preceding several months that involved community health workers.
**KPI S3: HTM integrated services offered to pregnant women**	1. HIV counselling and testing	
	2. ART for HIV+ women	
	3. IPT Provided at ANC	
	4. TB screening at ANC	• Current cough • Fever • Night sweats • Weight loss
**KPI S5: Systems readiness for CHWs**	1. Supportive supervision received by the CHWs in the last quarter from facility-based or district health staff covering their assigned health areas	
	2. Have a contract	• Scope of work • Full-time equivalent • Financial remuneration • Rest days • Leaves • Holidays • Health insurance
	3. Paid per contract	• Amount • Frequency • Timeliness
	4. No Stock outs commodities (e.g., medicines, test kits), equipment (e.g., thermometers, weighing scales), or job aids (e.g., registers, reporting forms) needed for CHW work during the reporting period	• Commodity • Equipment • Job aid
**Section 2: Programmatic indicators**		
**RSSH/PP HRH-6: Effective services composite**	1. Integrated services at ANC (TB, malaria, HIV) at the time of visit	
	2. Provider availability (absence rate on day of visit)	• All clinical providers supposed to be present at the facility at the time of the visit
	3. Provider caseload (number of outpatient visits per clinician per day)	• All providers with the primary responsibility of patient consultation
	4. ANC completion rate	• At least 4 ANC visits at 32 weeks or more
	5. DPT completion rate	• DPT 3 doses as a proportion of DPT 1 doses
	6. Treatment completion rate for new TB cases	
	7. 12-month retention on ART	
**CSS-3: Community-led monitoring**	1. Feedback on quality of services provided by clients	
	2. Trained community data collectors that routinely collect information on the availability, accessibility, acceptability, and quality of HIV, TB, or malaria services	
	3. Community provides feedback to this facility on (at least) a quarterly basis	
	4. Facility routinely analyzes and uses community-led monitoring data for quality improvement	
	5. Health care staff are aware of the community-led monitoring mechanism	
	6. Health care staff inform the clients about the community-led monitoring mechanism and its use.	
**HSG-1.1: Availability of clinical guidelines**	1. On-site availability of clinical guidelines for HIV, ART, TB, malaria, and/or primary health care (ANC, immunization, child health)	
**RSSH/PP RCS-1: Availability of functional oxygen systems**	1. Availability and functionality of Oxygen and delivery equipment	• Flow meter for oxygen source, with gradations in ml • Humidifier • Oxygen delivery apparatus (key connecting tubes and mask/nasal prongs)
	2. Functional Pulse oximeter	
**RSSH/PP LAB-5: Availability of essential diagnostics**	1. Availability of 27 Essential diagnostic tests for facilities with lab within the facility	• Facilities with lab included essential diagnostic tests on: ◦ HIV (4 tests) ◦ Malaria (1 test) ◦ TB (4 tests) ◦ Liver and Renal function (2 tests) ◦ Microbiological test (4 tests) ◦ Blood and Biochemistry test (5 tests) ◦ Other infectious diseases (7 tests) • Facilities without lab included essential diagnostic tests on: ◦ HIV (4 tests) ◦ Malaria (1 test) ◦ TB (1 test) ◦ Other infectious diseases (5 tests)
	2. Availability of 11 Essential diagnostic tests for facilities without lab within the facility	
**RSSH/PP HRH-1: Vacancy rate**	1. Proportion of full-time funded but currently vacant staff positions	• Physicians • Nurses and midwives • Lab technicians • Pharmacists • CHWs
**RSSH/PP HRH-4: Provision of high-quality HTM services by CHWs**	1. Quality of HIV, TB, and malaria service delivery by CHWs based on record reviews and monthly reports	• TB quality of care • Malaria quality of care• Pneumonia quality of care. • Percentage of recommended household visits done • Percentage of households with a child under five years old that slept under a long-lasting insecticide-treated net (LLIN)• Percentage of women who deliver at home that are referred to a health center• Percentage of children under 6 months old who are exclusively breastfed

ANC = antenatal care, ART = antiretroviral therapy, CHW = community health worker, DPT = diphtheria-pertussis-tetanus vaccine, HRH = human resources for health, IPTp = intermittent preventive treatment of malaria in pregnancy, IFA = Iron and Folic Acid, KPI = Key Performance Indicators, RSSH = Resilient and Sustainable Systems for Health, TB = tuberculosis.

The 11 indicators align with the Global Fund’s strategic monitoring framework and follow the terminology and coding system that corresponds to the thematic investment areas of the Global Fund Strategy 2023–2028. Each indicator is designed to capture service delivery processes (integrated service delivery and quality of care) and outcomes (patientreported experience), providing a pragmatic way to track health system performance across multiple countries. Table 2 describes the rationale, components, and data collection method for each indicator; detailed definitions of all attributes and the methodology for computing each indicator are provided in the supplementary material (S1 and S2 Annexes).

**Table 2:** Description of the 11 Targeted Health Facility Assessment (tHFA) indicators.

Indicator	Rationale	Components	Data collection method
***Section 1: Key Performance Indicators (KPIs)***			
**KPI S1: Provision of Integrated, People-** **Centred, High-Quality Services**	Measures the delivery of high-quality, integrated services at health facilities, incorporating technical and interpersonal aspects of care. Moves beyond input-based metrics to capture what happens at the point of care, emphasizing adherence to clinical protocols and respectful communication with patients.	Two key dimensions: (i) health worker (HW) competence/treatment and counselling provided to patients for sexual and reproductive health (SRH), antenatal care (ANC), and HTM delivered in an integrated fashion; and (ii) patient centeredness — patient-reported experience including communication, respect, autonomy, confidentiality, and social support.	Dimension 1: ANC, ART and TB record review (5 records each). Dimension 2: patient exit interviews (5 clients).
**KPI S2: Provision of Integrated Supportive Supervision**	Measures the components and quality of supportive supervision provided to HWs at the facility level. Routine, structured supervision contributes to higher adherence to clinical guidelines [15], improved service integration, and stronger staff motivation. In many settings, supervision remains irregular, disease-focused in scope, or punitive rather than constructive.	Frequency and scope of supervisory visits, including whether they cover multiple service areas (HTM), review records, offer feedback, and support group problem-solving.	Facility in-charge interview.
**KPI S3: HTM Integrated Services Offered to Pregnant Women**	Measures whether pregnant women receive a coordinated package of essential services across HTM domains during ANC visits. Pregnancy presents a critical opportunity to deliver multiple life-saving interventions, and fragmented service delivery can lead to missed diagnoses and preventable adverse outcomes.	Whether the facility provides: (i) HIV counselling and antiretroviral therapy (ART) for HIV-positive patients; (ii) intermittent preventive treatment of malaria in pregnancy (IPTp); and (iii) TB screening as part of routine ANC.	ANC record review (5 records per facility).
**KPI S5: Systems Readiness for CHWs**	Assesses whether health systems are structurally equipped to support CHWs who play a vital role in delivering primary care at the community level. Well-supported CHWs improve coverage and quality of services, especially for marginalized populations. However, many systems lack formal mechanisms to adequately train, compensate, and supervise them.	Availability of: formal contracts with detailed terms of reference; supervision mechanisms; supplies including key equipment, job-aids and commodities; and timely remuneration.	Structured in-person interviews with CHWs invited in advance to sampled facilities (up to 5 CHWs per facility).
***Section 2: Programmatic Indicators***			
**RSSH/PP HRH-6: Facilities Providing Effective Services**	Measures a facility’s service delivery capacity by combining performance across seven sub-indicators. Provides a snapshot of how effectively a health facility functions in delivering basic primary health services.	(i) Provider/staff availability on the day of visit; (ii) provider caseload (number of outpatient visits per clinician per day); (iii) ANC completion rate; (iv) TB treatment completion rate; (v) Diphtheria, Pertussis, and Tetanus (DPT) vaccine completion rate; (vi) HTM integration at ANC; and (vii) 12-month ART retention rate.	Record reviews and HW interviews.
**CSS-3: Community-Led Monitoring**	Gauges the presence of community-led monitoring mechanisms within health facilities [16]. Stems from the Global Fund’s investment strategy to promote accountability, community ownership, and data-driven advocacy, which can improve service responsiveness and equity.	Existence of: structured community-led monitoring processes (e.g., service user feedback mechanism); independent community leadership of these efforts; and integration of findings into facility improvement plans.	Facility in-charge interview.
**HSG-1.1: Availability of Clinical Guidelines**	Assesses whether up-to-date clinical guidelines for HTM and other primary health care services (e.g., ANC, immunization, child health) are available on-site. Increased access to standardized clinical protocols funded through Global Fund grants is a prerequisite for delivering quality and consistent care, helping reduce variability in practices and improve clinical outcomes.	Physical verification of printed or digital copies of relevant national guidelines during the facility visit.	Direct observation.
**RSSH/PP RCS-1: Availability of Functional Oxygen Systems**	Determines whether oxygen delivery systems (e.g., cylinders, concentrators, pulse oximeters, flowmeters) are available and functional in a facility. Recognizes the lack of standardized data across countries on oxygen functionality in routine information systems to monitor COVID-19-related large-scale investments in oxygen as a lifesaving essential element in managing severe conditions (e.g., pneumonia as well as COVID-19), reflecting the facility’s capacity to respond to critical care needs.[17]	Two key attributes: (i) availability of oxygen and delivery equipment (including functionality of flow meters for oxygen, humidifiers, and oxygen delivery apparatus); and (ii) availability of a functional pulse oximeter.	Direct observation.
**RSSH/PP LAB-5: Availability of Essential Diagnostics**	Checks whether a facility/laboratory has a predefined set of essential diagnostic tests based on the WHO Essential Diagnostics List with a focus on HTM services. Identifies diagnostic capacity as a critical bottleneck to accurate treatment and disease control.	A list of 27 essential diagnostic tests in facilities that have a laboratory and a list of 11 rapid and screening tests in facilities without a laboratory but that report conducting tests (e.g., in service delivery sites). The list is a sub-set of the WHO Essential Diagnostic List selected by Global Fund lab specialists to inform their investments in HTM services.	Direct observation.
**RSSH/PP HRH-1: Vacancy Rate**	Measures the proportion of full-time funded but currently vacant HW positions at a facility. Vacant posts signify systemic workforce shortages, which impair service delivery, reduce care quality, and limit a facility’s ability to implement integrated programming.	Identifying approved positions versus those currently filled for physicians, nurses, midwives, lab technicians, pharmacists, and CHWs.	Facility in-charge interview.
**RSSH/PP HRH-4: Provision of High-Quality HTM Services by CHWs**	Measures the extent to which CHWs deliver high-quality HTM services in line with national protocols. Reflects the critical role CHWs play in expanding access to essential services at the community level, while highlighting the need to assess not only their availability but also the quality of care they provide.	Core tasks such as case assessment, diagnostic testing, treatment, referral, and health promotion, as determined by review of CHW service records and reporting forms.	Abstraction of CHW registers and monthly reports.

ANC = antenatal care; ART = antiretroviral therapy; CHW = community health worker; DPT = diphtheria-pertussis-tetanus; HTM = HIV, TB and malaria; IPTp = intermittent preventive treatment of malaria in pregnancy; SRH = sexual and reproductive health; TB = tuberculosis. All indicators were measured in all 18 countries, except RSSH/PP HRH-4, which was measured only in DRC

#### Questionnaire structure.

The tHFA questionnaire has eight modules (Table 3), each designed for brevity and alignment with the 11 indicators. Screening questions on service availability (e.g., HIV treatment) in an early module allows for content filtering and quality-related questions for specific services (e.g., ART record review) in a subsequent module, which minimises respondent and data collector burden. The data collection methods chosen enable objectively verifiable data and do not require medically trained data collectors. All modules except patient exit interviews and CHW interviews incorporate the relevant and available questions from the HHFA. During the baseline survey, the questionnaire was pilot tested in field settings twice to refine the content and flow based on the understanding of the respondents and the interviewers.

**Table 3:** Summary of eight modules of the baseline Targeted Health Facility Assessment (tHFA) questionnaire.

Module	Data collection method	Key components	Total number of questions	Number of questions from HHFA
1.Facility identifiers	Facility in-charge interview	Location, level of care	16	14
2.Service availability & Readiness	Facility in-charge interview, direct observation	Services, staffing, guidelines, equipment	81	22
3.Supportive supervision	Facility in-charge interview	Scope, frequency, content	13	2
4.Patient-centeredness	Outpatient exit interview	Experience, respectful care, access challenges	18	5 questions from WHO PREMs
5.Support to CHWs	CHW interview	Support, supplies, remuneration	30	0
5a. and 5b. CHWs Record review	CHW client register book	Quality of care	31	0
6.Antenatal care	Record review	Service delivery record, tests, procedures	20	17
7.HIV services	ART record review	Service delivery record, tests, procedures	15	10
8.TB services	Record review	Service delivery record, tests, procedures	8	8

ART = antiretroviral therapy, CHW = community health worker, HHFA = Harmonized Health Facility Assessment, PREM = patient-reported experience measures, TB = tuberculosis, WHO = World Health Organization.

#### Sampling.

A master facility list (MFL) of all health facilities in each country was obtained by the Global Fund and updated with information on ANC and HTM service availability. Any facility that offered only specialized services such as maternity homes or dental clinics was excluded from sampling. All public sector health facilities were included in the MFL of each country and private sector facilities were included in Madagascar, Gambia, Liberia, Madagascar, Guinea-Bissau, Mali, Cameroon, and Niger.

**Fig 1:**
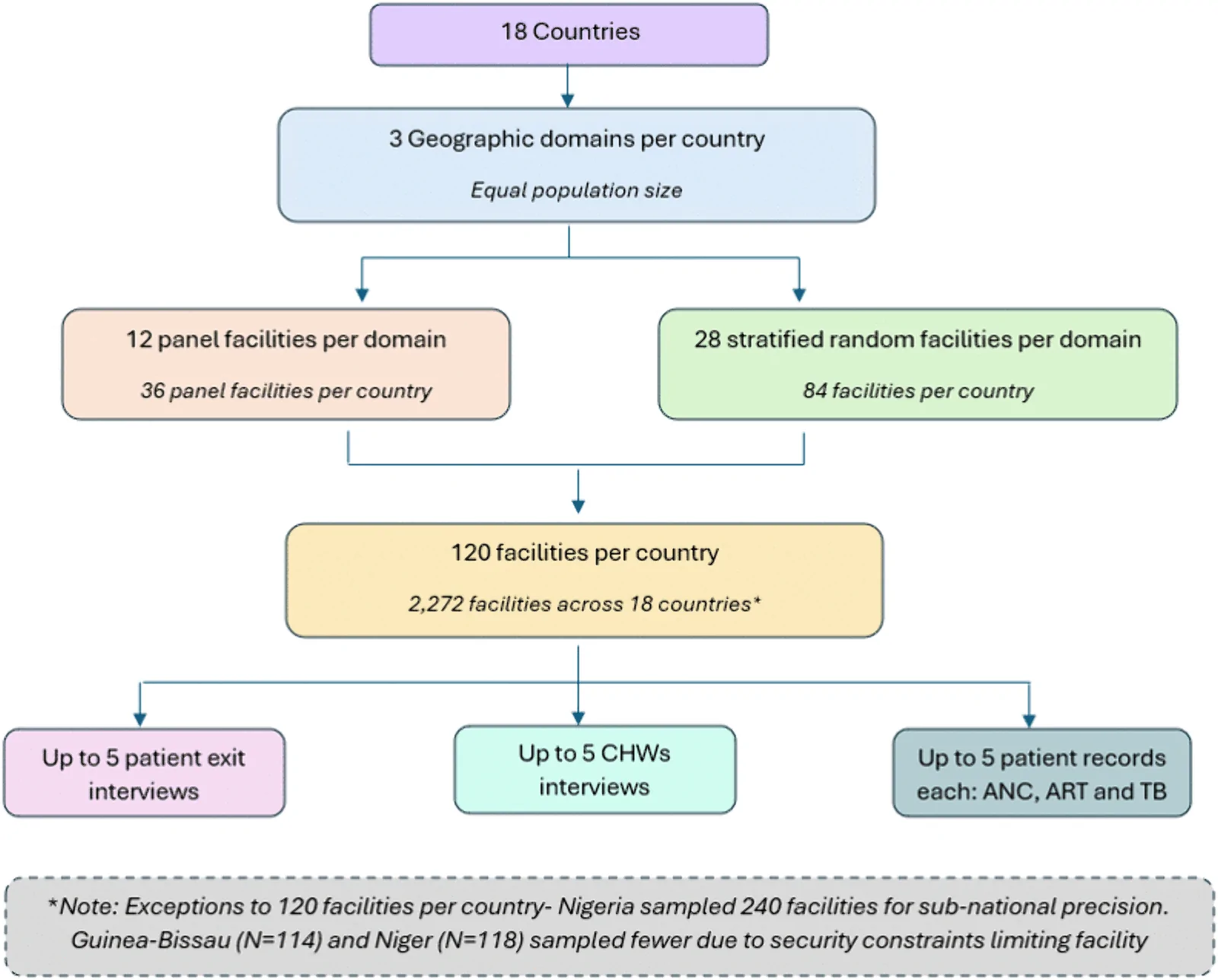
Sample size at country-level for the Targeted Health Facility Assessment (tHFA).

Each country was divided into three contiguous geographic domains, approximately equal in terms of population size and number of eligible primary health facilities. Facilities were then stratified by level of care provided: primary (health centres/posts), secondary (district/sub-district hospitals), and tertiary (referral/specialty hospitals) facilities. Altogether, 120 health facilities were selected using a two-stage sampling strategy (details below) in each country to balance representativeness at the national level with relevance for the Global Fund’s grant and program monitoring (Fig 1).

In stage 1, in each geographic domain, starting with the tertiary, then secondary and primary facility strata, 30% of the sample (12 facilities per domain) was randomly sampled from facilities that had large patient volumes and received direct support from the Global Fund. These facilities constitute the panel for future rounds of the tHFA during the strategy period. In stage 2, from the remaining pool of eligible facilities in each domain (including those not sampled in stage 1), stratified random sampling was used to select the remaining 70% of the sample (28 facilities per domain). Sampled facilities that were inaccessible due to security reasons were replaced with a randomly sampled facility from the same domain at the same level of care (primary, secondary or tertiary). On average, less than 10% of the initially sampled facilities were replaced due to security reasons. Post-stratification weighting was used to account for the sampling method, including facility replacement and other sampling deviations (weights = 1/facility selection probability). This allows for the estimation of a 95% confidence interval of ± 10 percentage points for facility-level indicators and a difference of 16 percentage points (or higher) between 2 rounds of tHFA. In each health facility, random sampling was used to select five outpatients (for exit interview questions on patient experience of care), five associated CHWs (for interview questions on support to CHWs) and extract data from five records for ANC, ART, and TB clients (for questions on integrated quality of care). In summary, sampling in each country approximated a probability based selection of eligible health facilities and respondents.

#### Data collection and quality assurance.

**Fig 2:**
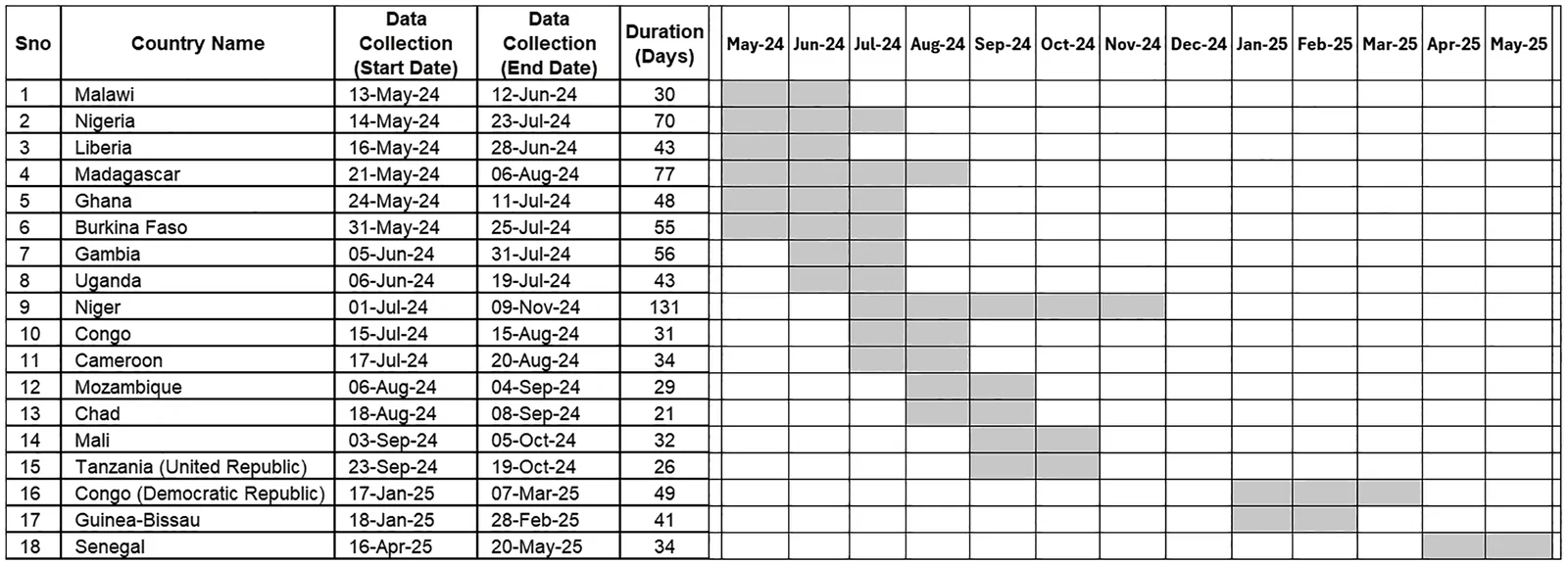
Data collection timelines in 18 countries (May 2024–May 2025).

The study collected all data in person, in-country, over four to six weeks, using tablet-assisted interviewing software (SurveyCTO; Dobility, Inc.; Cambridge, MA) to facilitate real-time quality checks and secure transmission. Two agencies managed the data collection: IQVIA (in 13 countries) and KPMG (in five countries) during the period of May 2024 to May 2025 (Fig 2), to coincide with the signing of the Global Fund country grants, and the data collection for most countries completed in approximately 4–6 weeks.

The average cost of data collection and database management for the tHFA in the 18 countries was US$ 70,000 per country (range of US $ 55,000–90,000).

Local data collectors with appropriate credentials, experience and language skills in each country underwent five to six days of training that included protocol review, questionnaire administration, field practice, research ethics (including written informed consent), privacy, and correct use of tablets. The team embedded comprehensive quality assurance (QA) throughout the tHFA implementation, through training, pilot-testing, and real-time monitoring, including before, during, and after data collection (Table 4). Independent data quality assurance (IDQA) was implemented in four of the 18 countries (Burkina Faso, Malawi, Guinea-Bissau and Chad) and data on a small sub-set of the tHFA questions were recollected in 19 of the 120 facilities within a week of the initial data collection. The agreement between question responses from the initial data collection and the IDQA was 80% or higher.

**Table 4:** Data quality assurance for tHFA baseline implementation in 18 countries (May 2024–May 2025).

Before data collection	During data collection	After data collection
• Two rounds of pilot testing of the questionnaire • Hiring of qualified data collectors with field experience and language skills • Five- to six-day rigorous training and pre and post training test of survey teams • Guidance documents including field and survey manuals • Validation of master data frame before initiation of data collection	• Centralized real-time data collection using a standardized tool • GPS and time stamping of collected data • End-to-end data management protocol and daily feedback from the Global Fund project team • Standard real-time data quality assessment of 10% of questions by field supervisor • Spot check at 5% of facilities and telephonic confirmation at 50% of facilities	• Check for inconsistencies and outliers within data files • Check for completeness and adherence to replacement protocols (selecting the replacement facility of the same level of care as that of original) • Independent data quality assurance in Burkina Faso, Malawi, Guinea-Bissau, and Chad (19 facilities each)

## Discussion

This paper describes the development and implementation of the baseline round of tHFA in 18 countries to monitor the Global Fund’s strategic objective of delivering IPCQS towards the goal of eliminating HTM. To our knowledge, tHFA is the first multi-country, nationally representative effort to monitor health service delivery processes and outcomes for the Global Fund grants and the national programs being implemented in these countries. By integrating insights on both disease-specific service delivery processes and system-level outcomes, the tHFA is designed to improve understanding of contribution of horizontal health systems strengthening investments to disease-specific programs. Institutionalized at the Global Fund, aligned with WHO’s IPCHS framework, it responds to the Lancet Global Health Commission’s call to increase the focus on service delivery processes and outcomes to improve the performance of health systems. It is also designed to address a gap in most health facility assessments by including CHW readiness and patient-reported experience, in line with the IPCHS principle of engaging communities and frontline providers.

Institutionally embedding the tHFA into the regular grant and strategic monitoring mechanisms of the Global Fund is intended to support its sustainability and rapid implementation with timely data collection, specifically, by using existing processes for coordinating between technical and country teams and data collection agencies and ministries of health. The tHFA’s short, targeted set of indicators and reliance on methods of data collection (facility observation, interviews, and record review) that do not require surveyors to have specialized clinical knowledge helped optimize field costs. The use of a standardised, modular approach incorporating relevant questions from HHFA was designed to allow for comparability across settings while aiming to maintain relevance to country-specific systems.

The targeted focus on measuring service delivery processes and outcomes, coupled with relatively low cost compared to existing HFAs, can allow the tHFA to be agile and support repeated assessments over the five-year Global Fund strategy cycle for timely decision-making. The modular architecture is designed to provide the flexibility to add country-specific questions while aiming to maintain KPI comparability. Close partnership with MoH through shared decision-making is intended to promote mutual accountability and institutionalization, increasing the likelihood of sustained use for national planning, grant monitoring and incorporation of health systems outcomes into future grant applications. Future follow-up would determine the degree to which the methods and indicators are being adopted and financed by the country governments through inclusion into country grant proposals and other funding applications.

With recent reduction in the official development assistance (ODA) for Global Health, specifically US government funding for large scale health facility assessments (HFAs) like the SPA and HHFA, the tHFA can serve as cost-efficient platform for Global Health Institutions (GHI’s) to monitor investments through reliable data on service delivery processes and outcomes at the point of service delivery. The tHFA could facilitate alignment with the principles of mutual accountability, ownership and local leadership in health system strengthening efforts articulated in the Lusaka Agenda, a consensus document developed by stakeholders around the world regarding the future of the GHIs [18]. This collaborative approach aligns with IPCHS priorities on creating enabling environments and the Lancet Commission’s call for stronger health system leadership [2,6]. In the long term, the tHFA is envisioned as a tool that ministries of health and national statistics offices can institutionalize within their routine health facility surveys, building national capacity for periodic, self-financed monitoring of integrated, people-centred service delivery. As country ownership deepens and data collection costs are further optimized, adoption by sub-national health management teams for district-level decision-making would further strengthen its utility for sub-national health management.

The tHFA has limitations, however. First, the cross-sectional design does not allow measurement of changes in service integration and quality of care for a given patient over time, especially when management of conditions included in the assessment requires multiple visits to a health facility. The longitudinal measurement of the process and outcomes of care for pregnant women is part of the Maternal and Newborn Health (MNH) eCohort [19] and the Performance Monitoring and Accountability (PMA) [20] studies and could be included in the patient exit interviews for future rounds of tHFA. Second, the data on service integration and quality were abstracted from paper-based patient records, which may yield incomplete or inaccurate information, compared to other methods of data collection, such as direct observation of service delivery, clinical vignettes, or mystery clients [21,22]. Available literature indicates an increase in availability of electronic health records in LMICs [23], use of which can improve the accuracy of estimates in future rounds. Moreover, where methodologically feasible, the paper-based data collection of tHFA can be complemented with faster methods for data collection, for example the Global Financing Facility (GFF) led Frequent Assessment & Health Systems tools for Resilience (FASTR) initiative [24] that collects data from the facility in-charge via phone interviews to identify and reduce service delivery constraints in PHC facilities. Third, social desirability bias during the patient exit interview may cause over-reporting of service quality. Existing literature suggests that conducting phone-based exit surveys and collecting data after a few days of the facility visit can avoid this bias and could be implemented in future rounds [25]. Fourth, while tHFA questionnaire was pilot tested before in-country rollout, patients were not directly involved in the development of the tHFA questions related to patient-centeredness (KPI S1, Dimension 2). These questions are drawn from the WHO PREMs and WHO HHFA, both globally validated instruments, providing confidence that these items have been tested across diverse LMIC settings. Greater engagement of patients and community representatives in adapting and refining the tool for specific country contexts remains an important priority for future rounds, to ensure that the attributes measured align with the priorities and lived experiences of the populations served. Finally, some structural indicators such as guideline availability (HSG-1.1) and vacancy rates (HRH-1) are best interpreted directionally. Low scores on these indicators reliably signal health system weaknesses that require urgent attention, whereas high scores indicate enabling conditions for quality care but do not, on their own, confirm that integrated, people-centred services are being delivered [12]. Notwithstanding these limitations, given the dearth of data on IPCQS in these countries and a historical preponderance of input-based monitoring metrics in HFAs, the tHFA can provide valuable data that can be used to improve performance of health systems in LMICs.

The baseline tHFA results across the 18 countries are being written up in a separate manuscript. To assess whether the tHFA is meeting its objectives as a monitoring tool, a formal evaluation framework will be applied to future rounds, examining four dimensions: (1) data quality, capturing whether data are complete, consistent, and reproducible; (2) cost-efficiency, examining whether cost per-round remain lower than existing HFAs; (3) use of results, assessing whether findings inform grant monitoring, country plans, and programme decisions; and (4) sustainability and country ownership, reflecting whether ministries of health are integrating the tHFA into national monitoring frameworks and independently financing future rounds.

The progress on Global Fund’s strategic KPIs is based on change in tHFA indicators, therefore, we expect that any bias in measurement at baseline will remain constant when comparing 2 data sets collected the same way and in the same country at two points in time. Future rounds will require continued coordination between the Global Fund and the established national partners to maintain a core questionnaire and sampling consistency for KPI comparability, while retaining flexibility for country-specific priorities. In the face of constrained funding, the tHFA can offer a scalable platform for GHIs to work with LMIC institutions in building a country-led, harmonized and efficient monitoring system for stronger health systems focused on mutual accountability to support efforts to improve actual services delivery.

## Conclusion

The tHFA is a pragmatic, agile, scalable platform designed to monitor integrated service delivery and people-centred quality of care in LMICs. With a focused set of process- and outcome-oriented measures, inclusion of CHWs, and alignment with the Global Fund Strategy (2023–2028), it is designed to support decision-making, programme monitoring, and health-system strengthening. By linking disease-specific delivery with system outcomes, it has the potential to bridge the vertical–horizontal divide. Partnership with ministries of health is intended to foster accountability, institutionalization and integration in national plans. Its modular design is intended to adapt to country priorities while aiming to maintain KPI comparability, allowing for repeated use for performance management and HSS tracking in a mutually accountable manner.

## Supporting information

S1 Annex Indicator details of The Global Fund’s Targeted Health Facility Assessment (t-HFA) for use in the Democratic Republic of the Congo (DRC). (DOCX)

S2 Annex Questionnaire for The Global Fund’s Targeted Health Facility Assessment for use in the Democratic Republic of the Congo (DRC). (DOCX)

S1 Table IRB Approval authority and reference number by Country. (DOCX)

## References

[1] Bitton A, Ratcliffe HL, Veillard JH, Kress DH, Barkley S, Kimball M, et al. Primary Health Care as a Foundation for Strengthening Health Systems in Low- and Middle-Income Countries. J Gen Intern Med. 2017;32(5):566–71. doi: 10.1007/s11606-016-3898-5 27943038 PMC5400754

[2] Kruk ME, Gage AD, Arsenault C, Jordan K, Leslie HH, Roder-DeWan S, et al. High-quality health systems in the Sustainable Development Goals era: time for a revolution. Lancet Glob Health. 2018;6(11):e1196–252. doi: 10.1016/S2214-109X(18)30386-3 30196093 PMC7734391

[3] World Health Organization, World Bank. Tracking universal health coverage: first global monitoring report. Geneva: World Health Organization. 2015. https://www.who.int/publications/i/item/9789241564977

[4] Leslie HH, Malata A, Ndiaye Y, Kruk ME. Effective coverage of primary care services in eight high-mortality countries. BMJ Glob Health. 2017;2(3):e000424. doi: 10.1136/bmjgh-2017-000424 29632704 PMC5887868

[5] World Health Organization. World health report 2013: Research for universal health coverage. 2013. https://iris.who.int/handle/10665/85761

[6] World Health Organization. Framework on integrated, people-centred health services: report by the Secretariat. Geneva: World Health Organization. 2016. https://apps.who.int/gb/ebwha/pdf_files/WHA69/A69_39-en.pdf?ua=1&ua=1

[7] World Health Organization, United Nations CF. Operational Framework for Primary Health Care: Transforming Vision into Action. Geneva: World Health Organization. 2020.

[8] World Health Organization. Declaration of Astana. Geneva: World Health Organization. 2019. https://iris.who.int/bitstream/handle/10665/328123/WHO-HIS-SDS-2018.61-eng.pdf

[9] The Global Fund. Fighting pandemics and building a healthier and more equitable world: Global Fund strategy (2023–2028). Geneva: The Global Fund to Fight AIDS, Tuberculosis and Malaria. 2021. https://www.theglobalfund.org/media/11612/strategy_globalfund2023-2028_narrative_en.pdf

[10] The Global Fund. Key Performance Indicators (KPIs) Handbook for the 2023-2028 Strategy. 2023. https://www.theglobalfund.org/media/12681/strategy_globalfund2023-2028-kpi_handbook_en.pdf

[11] Asefa A, Dossou J-P, Hanson C, Hounsou CB, Namazzi G, Meja S, et al. Methodological reflections on health system-oriented assessment of maternity care in 16 hospitals in sub-Saharan Africa: an embedded case study. Health Policy Plan. 2022;37(10):1257–66. doi: 10.1093/heapol/czac078 36087095 PMC9661265

[12] Leslie HH, Sun Z, Kruk ME. Association between infrastructure and observed quality of care in 4 healthcare services: A cross-sectional study of 4,300 facilities in 8 countries. PLoS Med. 2017;14(12):e1002464. doi: 10.1371/journal.pmed.1002464 29232377 PMC5726617

[13] World Health Organization. Patient-reported experiences in primary care: metrics and assessment tool, rapid version. Geneva. 2025. https://www.who.int/publications/i/item/9789240112438

[14] The Global Fund. Modular framework handbook. The Global Fund. 2023. https://resources.theglobalfund.org/media/13907/cr_modular-framework_handbook_en.pdf

[15] Rowe AK, Rowe SY, Peters DH, Holloway KA, Chalker J, Ross-Degnan D. Effectiveness of strategies to improve health-care provider practices in low-income and middle-income countries: a systematic review. Lancet Glob Health. 2018;6(11):e1163–75. doi: 10.1016/S2214-109X(18)30398-X 30309799 PMC6185992

[16] UNAIDS. Establishing community-led monitoring of HIV services: principles and process. Geneva: UNAIDS. 2021. https://www.unaids.org/en/resources/documents/2021/establishing-community-led-monitoring-hiv-services

[17] Ijaz N, Lee T, Furtado N, Macher E, Muitheri Z, Park BJ, et al. A rapid facility-level assessment of oxygen systems in 39 low-income and middle-income countries: a cross-sectional study. Lancet Glob Health. 2025;13(4):e646–55. doi: 10.1016/S2214-109X(24)00561-8 40024266 PMC11954661

[18] Future of Global Health Initiatives. The Lusaka Agenda: Conclusions of the Future of Global Health Initiatives Process. 2023. https://d2nhv1us8wflpq.cloudfront.net/prod/uploads/2023/12/Lusaka-Agenda.pdf

[19] Arsenault C, Wright K, Taddele T, Tadele A, Derseh Mebratie A, Tiruneh Tiyare F, et al. The maternal and newborn health eCohort to track longitudinal care quality: study protocol and survey development. Glob Health Action. 2024;17(1):2392352. doi: 10.1080/16549716.2024.2392352 39163134 PMC11338195

[20] Performance Monitoring for Action (PMA). PMA Data. Baltimore (MD): Johns Hopkins Bloomberg School of Public Health. https://www.pmadata.org/

[21] Martin SK, Hall MAK, Molitch-Hou E, Benaderet A, Park JJ, Sweet M. Case to Vignette: A Framework to Elevate Narrative by Embracing Vignette-Based Learning in Medicine. J Gen Intern Med. 2025;40(13):3223–7. doi: 10.1007/s11606-025-09531-5 40281275 PMC12508412

[22] Fitzpatrick A, Tumlinson K. Strategies for Optimal Implementation of Simulated Clients for Measuring Quality of Care in Low- and Middle-Income Countries. Glob Health Sci Pract. 2017;5(1):108–14. doi: 10.9745/GHSP-D-16-00266 28126970 PMC5493448

[23] Ngugi P, Babic A, Kariuki J, Santas X, Naanyu V, Were MC. Development of standard indicators to assess use of electronic health record systems implemented in low-and medium-income countries. PLoS One. 2021;16(1):e0244917. doi: 10.1371/journal.pone.0244917 33428656 PMC7799790

[24] The Global Financing Facility and the World Bank Group. FASTR: The GFF’s approach to rapid cycle analytics and data use. 2024. https://data.gffportal.org/key-theme/FASTR

[25] Shamebo D, Derseh Mebratie A, Arsenault C. Using an Interactive Voice Response Survey to Assess Patient Satisfaction in Ethiopia: Development and Feasibility Study. JMIR Form Res. 2025;9:e67452. doi: 10.2196/67452 39946713 PMC11892327

