# Supplementary material for: Design and implementation of a multi-country targeted health facility assessment to measure integrated, people-centered, quality of services
Source: PLOS Glob Public Health. 2026 Aug 19;6(8):e0005736. doi: 10.1371/journal.pgph.0005736 (PMC13489417; doi:10.1371/journal.pgph.0005736)
Supplement: Supplementary file 1 [file pgph.0005736.s001.docx]

**S1 Annex. Indicator details of The Global Fund’s Targeted Health Facility Assessment (t-HFA) for use in the Democratic Republic of the Congo (DRC)**

**Purpose of this document**

This annex describes t-HFA operational indicator definitions, additional documentation about indicators from The Global Fund’s KPI Handbook and Indicator Guidance Sheet (IGS), measurement methods, analysis methods, documentation on why certain decisions were made, some questionnaire items (for indicators not in the WHO Harmonized Health Facility Assessment [HHFA]), and question numbers (i.e., numbers that correspond to t-HFA questionnaire items) for each t-HFA indicator (which will be needed to analyze t-HFA data). There is a separate questionnaire document with detailed methods and questions (Protocol Annex 2). Altogether, eleven RSSH t-HFA indicators are described. Regarding methods, there is a description of whether each t-HFA indicator can be measured by the HHFA (and if so, which specific HHFA indicators or questions were used).

**How this document is organized**

Section A presents key performance indicators (KPIs), and Section B presents non-KPIs. The eleven t-HFA indicators are presented using the following 5-part format: a) indicator code and name; b) sources used to develop the indicator definition and measurement methodology; c) indicator definition; d) measurement methodology; and e) comments. Most indicators also have an analysis method and a table or section of text that describes which questions in the t-HFA questionnaire are used to measure the indicator.

**Where to find details on HHFA indicators**

WHO’s online HHFA Indicator Inventory (<https://indicator-inventory.hhfa.online/>) has details on HHFA indicators.

**SECTION A. KEY PERFORMANCE INDICATORS**

**1.a. KPI S1: “Provision of integrated, people-centered, high-quality services”**

1.b. Sources: Slides 51–53 of the KPI Handbook, row 27 of the IGS (indicator RSSH/PP HRH-7), and some WHO/UNICEF sources described in the Comments.

1.c. Definition

This indicator has two dimensions:

1. Health worker (HW) competence/treatment and counseling provided to patients delivered in an integrated fashion. I.e., Adherence to clinical standards/guidelines for tracer services (SRH, ANC, HTM). Assessment will be based on review of patient records. This dimension has 13 attributes (see Table 1, below).

2. Patient centeredness. Patients that report meeting key attributes of patient experience (communication, respect, autonomy, confidentiality, and social support). Assessment based on patient exit interviews. This dimension has 9 attributes (see Table 1, below).

Note: This definition has been slightly revised and clarified (see Comments).

1.d. Measurement methodology

Measurement methods for each dimension are described below, as well as an analysis method for summarizing the dimensions into a composite score. Also, see Table 1, below.

**Dimension 1. HW competence/treatment and counseling for SRH, ANC, and HTM delivered in an integrated fashion**

- Method is record review with 5 ANC clients per facility. For details on sampling, see t-HFA Annex 2, which is based on page 13 of WHO’s 2023 HHFA Module 3 (Quality of care [QoC]).
- For simplicity and alignment with WHO norms, the HHFA method was selected. The integrated ANC and HTM attributes are covered by KPI S3 (HTM integrated services offered to pregnant women; see below on page 10). The integrated SRH attribute is the “ANC testing for syphilis” indicator (HHFA indicator 5.1.1.1.9; permanent ID “DAP”). Note that screening is not, technically speaking, “treatment and counseling”; but it still fits under the broader “HW competence” domain of the indicator. To broaden the scope of the QoC assessment, the ANC record review will also include attributes on the following: 1) whether gestational age was recorded at the last ANC visit, 2) blood pressure measurement (HHFA indicator 5.1.1.1.4: permanent ID “DAR”; percentage of ANC clients with blood pressure measured at most recent ANC contact), 3) iron and folate supplements (HHFA question 13004-10), 4) danger signs (HHFA question 13004-11), 5) treatment of intestinal worms (HHFA indicator 5.1.1.1.12: permanent ID “CIB”; percentage of ANC clients with any medicine for intestinal worms received during ANC), and 6) hemoglobin measurement (HHFA question 13005-05).
- A second record review (of ART clients) will be done to measure attributes of integrated HIV/TB care: HHFA indicator 5.3.2.3.5 (Permanent ID "DET"; Percentage of ART clients with active TB and currently enrolled in TB treatment) and an indicator on TB screening for ART clients at the most recent clinical visit (which is similar to, but not exactly the same as, HHFA indicator 5.3.2.1.8 [Permanent ID "DBK"; Percentage of ART clients with standard screening for TB recorded at most recent clinical visit]—see explanation below). For details on sampling, see t-HFA Annex 2 (page 28), which is based on page 37 of WHO’s 2023 HHFA Module 3 (QoC). Note that the additional HIV/TB integrated care would not necessarily be integrated with the ANC care because the additional HIV/TB record review would probably examine different clients. To broaden the scope of the QoC assessment, the ART client record review will also include whether blood pressure was recorded (see Table 1).
- A third record review (of TB client) will be done to assess the general TB QoC with HHFA indicators 5.4.1.1.10 (Permanent ID “DFX”; Percentage of TB clients with changes in symptoms recorded at every clinical visit) and 5.4.1.1.11 (Permanent ID "DFZ"; Percentage of TB clients with HIV status recorded at least once).
- Items for questionnaire.
  - SRH. See methods for HHFA indicator 5.1.1.1.9 (Permanent ID "DAP"; Percentage of ANC clients with screening for syphilis received during ANC).
  - ANC. Use result from KPI S3 (HTM integrated services offered to pregnant women; see below).
  - Integrated HIV/TB screening and treatment. For TB screening among ART clients, five questions are asked: four symptom-specific questions (cough, fever, night sweats, and weight loss), and a fifth question that indicates that the HW screened for TB without documenting the presence or absence of each of the four TB symptoms (e.g., the HW recorded “TB screening negative”). TB screening is considered done if **either** the client’s chart shows that all four TB symptoms have been assessed **or** there is a more general note in the client’s chart that TB screening was done (e.g., “TB screening negative”). Note that HHFA indicator 5.3.2.1.8 (Permanent ID "DBK"; Percentage of ART clients with standard screening for TB recorded at most recent clinical visit) was **not** used because this indicator is measured with questions that only ask about three of the four WHO-recommended TB screening questions (cough, fever, and weight loss). Two other HHFA indicators are also used to measure integrated HIV/TB care: indicator 5.3.2.3.5 (Permanent ID "DET"; Percentage of ART clients with active TB and currently enrolled in TB treatment) and indicator 5.4.1.1.11 (Permanent ID "DFZ"; Percentage of TB clients with HIV status recorded at least once).

**Dimension 2. Patient centeredness**

- The measurement method is exit interviews with 5 clients per facility. Sampling: Select the first outpatient at the end of his/her visit to the facility; when that first interview is complete, select the very next outpatient encountered, and so on. Note that a potential limitation of this sampling approach is that QoC in mornings and afternoons could vary, and the t-HFA method would likely oversample clients seen in the morning. The method was selected for its simplicity.
- Items for questionnaire (see Comments for source of questions). The first five questions are measured on a Likert-scale and begin with an introduction that surveyors will read to potential subjects and a request for consent. Response choices are: never, rarely, sometimes, often, always, and don’t know.
  - Communication. Q132. Did your primary care professionals speak to you in a way you could understand?
  - Respect. Q122. Did your primary care professionals treat you with respect?
  - Autonomy. Q125. Did you feel like you were involved in making decisions about your care as much as you wanted?
  - Confidentiality. Q119. Did you believe your primary care professionals would keep your information confidential?
  - Social support. Q129. Did your primary care professionals discuss with you the care and support you receive from family members, friends, and others?
  - There are also five other questions added by t-HFA team:
    1) D003. Did the HW tell you what illness you have? [responses: yes/no/don’t know]
    2) D004 and D005. Could the patient recall a danger sign that would require returning to the facility immediately? [responses for D004: yes/no/don’t know; responses for D005 are danger signs]
    3) D006. Did a worker at the facility shout, scold, insult, threaten, or treat you rudely? [responses: yes/no/don’t know]
    4) D011. What are the key challenges/issues that you face while seeking care at this facility? [multiple responses]

Table 1. Indicators and survey questions on integrated, people-centered, high-quality services

| **Topic** | **HHFA indicator** | **t-HFA survey questions** |
| --- | --- | --- |
| Dimension 1: HW competence/treatment and counseling for SRH, ANC, and HTM delivered in an integrated fashion | | |
| Attribute 1.1. Integrated ANC and HTM care | - Indicators for 1) HIV counselling and testing, 2) access to ART for HIV-positive women, 3) IPTp given at ANC, and 4) TB screening are described in Table 3. | See Table 3 |
| Attribute 1.2. Integrated ANC and SRH | - Indicator 5.1.1.1.9 (Permanent ID "DAP"; Percentage of ANC clients with screening for syphilis received during ANC). | 13005_01 |
| Attribute 1.3. General ANC (gestational age recorded) | - No HHFA indicator, but this HHFA question was used: “What was the recorded gestational age (in weeks) at the most recent ANC visit?” | 13004_01 |
| Attribute 1.4. General ANC (blood pressure recorded) | - Indicator 5.1.1.1.4 (Permanent ID “DAR”; Percentage of ANC clients with blood pressure measured at most recent ANC contact) | 13004_08 |
| Attribute 1.5. General ANC (iron and folic acid) | - No HHFA indicator, but this HHFA question was used: “Is it documented that the client was provided or prescribed iron and folic acid during the most recent visit?” | 13004_10 |
| Attribute 1.6. General ANC (danger signs) | - No HHFA indicator, but this HHFA question was used: “Is there documentation that the client received counseling on pregnancy danger signs at the most recent visit?” | 13004_11 |
| Attribute 1.7. General ANC (treat intestinal worms) | - HHFA indicator (Permanent ID “CIB”; Percentage of ANC clients with any medicine for intestinal worms received during ANC) | 13005_02 |
| Attribute 1.8. General ANC (hemoglobin) | - No HHFA indicator, but this HHFA question was used: “Is any hemoglobin or hematocrit result documented for any ANC visit during this pregnancy?” | 13005_05 |
| Attribute 1.9. Integrated HIV and TB care (TB screening) | - Four symptom-specific questions (cough, fever, night sweats, and weight loss) and one non-specific TB screening question. Note that HHFA indicator 5.3.2.1.8 (Permanent ID "DBK"; Percentage of ART clients with standard screening for TB recorded at most recent clinical visit) is **not** used. See explanation above. | 13408_01, 13408_02, 13408_03,  G003, and G004. |
| Attribute 1.10. Integrated HIV and TB care (TB treatment) | - Indicator 5.3.2.3.5 (Permanent ID "DET"; Percentage of ART clients with active TB and currently enrolled in TB treatment) | 13408_08 and 13408_09 |
| Attribute 1.11. Integrated HIV and TB care (TB status recorded) | - Indicator 5.4.1.1.11 (Permanent ID "DFZ"; Percentage of TB clients with HIV status recorded at least once) | 13507_01 |
| Attribute 1.12. Blood pressure recorded for ART clients | - No HHFA indicator. Question developed by t-HFA team. | G001 |
| Attribute 1.13.  Changes in TB symptoms recorded | - Indicator 5.4.1.1.10 (Permanent ID “DFX”; Percentage of TB clients with changes in symptoms recorded at every clinical visit) | 13510_01 |
| Dimension 2: Patient centeredness | | |
| Attribute 2.1. Communication | - No HHFA indicator. Question developed by WHO.* | Q132 |
| Attribute 2.2. Respect | - No HHFA indicator. Question developed by WHO.* | Q122 |
| Attribute 2.3. Autonomy | - No HHFA indicator. Question developed by WHO.* | Q125 |
| Attribute 2.4.  Confidentiality | - No HHFA indicator. Question developed by WHO.* | Q119 |
| Attribute 2.5.  Social support | - No HHFA indicator. Question developed by WHO.* | Q129 |
| Attribute 2.6. HW told diagnosis | - No HHFA indicator. Question developed by GF t-HFA team. | D003 |
| Attribute 2.7. Patient recalled danger sign | - No HHFA indicator. Question developed by GF t-HFA team. | D004 and D005 |
| Attribute 2.8. Worker rudeness | - No HHFA indicator. Question developed by GF t-HFA team. | D006 |
| Attribute 2.9. Multiple factors | - No HHFA indicator. Question developed by GF t-HFA team. | D011 |

* Questions developed by WHO Special Program on Primary Health Care. “*WHO (2023). Patient-reported experiences in primary care: Patient questionnaire.*” Draft. (Personal communication from Dirk Horemans on April 18, 2023).

**Analysis method**

The method must account for the differing number of attributes that could apply to a given facility (e.g., not all facilities provide ANC and HIV services), the differing number of clients for whom data were collected at a given facility (e.g., it might not be possible to complete five exit interviews), and missing data (e.g., a client might respond “don’t know” to a question during the exit interview). For a given client record or exit interview, each question is scored on a scale of 0–100 (a higher score means better quality). Binary items (e.g., did a pregnant woman get at least 1 dose of IPTp?) get a value of "100" if the service was provided or a "0" if the service was not provided. For the exit interview question on whether the client was treated rudely (shouted at, scolded, insulted, or threatened), the attribute gets a score of “0” if the client was treated rudely or “100” if not treated rudely. For exit interview questions on danger signs, the score is “100” if the patient responds “YES” that the HW mentioned a danger sign (question D004) **and** the patient recalls a danger sign (question D005, responses 1–7); but the score is “0” if **either** the patient responds that the HW did not mention a danger sign (“NO” to question D004) **or** the patient cannot recall it (“DON’T KNOW” to question D005). For the exit interview question on key challenges/issues (question D011), the score is “100” if none of the key challenges/issues are reported (i.e., none of the response options “A” to “H” are mentioned), and the score is “0” if at least one key challenge/issue is reported (i.e., at least one of the response options “A” to “H” is mentioned). If the only response is “OTHER” (i.e., “J”) **and** the response specified in “J1” is a challenge or issue (e.g., “I could not communicate with the health worker because he did not speak my language“), then the score is “0”. If the only response is “OTHER” (i.e., “J”) **and** the response specified in “J1” indicates that there are no challenges or issues (e.g., “My visit went well. I cannot think of any problems.“), then the score is “100”. Likert-scale questions get a "0" for never, "25" for rarely, "50" for sometimes, "75" for often, or a "100" for always. For TB screening in the ANC and ART record review, the score is “100” if all four symptoms are recorded in the ANC record (current cough, fever, night sweats, and weight loss — each recorded as present or absent); otherwise, the score is “0”. If any question has a response of “don’t know”, it is excluded from the calculation of the score (except for D005, see above). For the attribute “ART clients with active TB and currently enrolled in TB treatment”, if the question "What was the client’s TB status at the most recent time this was documented?" (question 13408_08) has a response of either "LATENT TB", "NO TB INFECTION", or "NOT DOCUMENTED", it is excluded from the calculation of the score. For each surveyed client (assessed by record review or exit interview), calculate a score between 0 and 100 (a higher score means better quality) that equals the unweighted average of question-specific scores for questions that apply to the client. For each of the two indicator dimensions (HW competence and patient-centeredness), calculate a score between 0 and 100 (a higher score means better quality) that equals the unweighted average of client-specific scores for the clients assessed for the dimension. The indicator value for a given facility equals the unweighted average of the two dimension-specific scores. With this approach, one can calculate a mean score (from 0 to 100) for an individual facility, group of facilities (e.g., in a geographic province, or a facility type), entire country, or group of countries. Below is a summary of the steps needed to calculate values for KPI S1.

1) For each ANC client assessed by record review, calculate a client-specific score by averaging the 11 item-specific scores (i.e., HIV counseling and testing, access to ART for HIV-positive women, TB screening, IPTp, syphilis screening, gestational age recorded, blood pressure measured, iron/folate provided, danger sign counseling, worm treatment, and hemoglobin measured).
2) For each ART client assessed by record review, calculate a client-specific score by averaging the three item-specific scores (i.e., TB screening for patients on ART, TB treatment for ART patients with active TB, and blood pressure measured).
3) For each TB client assessed by record review, calculate a client-specific score by averaging the two item-specific scores (i.e., HIV status recorded, and changes in symptoms recorded).
4) Calculate a dimension-specific score for Dimension 1 (HW competence) by averaging all the ANC, HIV, and TB client-specific scores. Note that there could be up to five ANC client records, five ART client records, and five TB client records, so the score for Dimension 1 could be based on the average of up to 15 client-specific scores.
5) For each client exit interview, calculate a client-specific score by averaging the nine item-specific scores on patient centeredness (e.g., communication, respect, autonomy, etc.).
6) Calculate a dimension-specific score for Dimension 2 (patient centeredness) by averaging all the patient-specific scores. Note that there could be up to five client exit interviews, so the score for Dimension 2 could be based on the average of up to five client-specific scores.
7) Calculate a facility-specific score by averaging the facility’s Dimension 1 and Dimension 2 scores.
8) Calculate the value of KPI S1 for a country by averaging all the facility-specific scores.

1.e. Comments

- Regarding the indicator definition, this indicator was originally defined as having 3 dimensions (competence, treatment and counseling, and patient centeredness). As the definition of “competence” (i.e., “adherence to clinical standards/guidelines”) largely overlapped with the “treatment and counseling” dimension, these were combined.
- Regarding the definition of “patient centeredness”, page 121 of “*WHO and UNICEF (2022). Primary health care measurement framework and indicators: monitoring health systems through a primary health care lens. Web Annex. Technical specifications*”, which describes PHME indicator 74 (source cited for indicator RSSH/PP HRH-7) on “Patient-reported experiences”, states that “people-centeredness” includes: 1) communication, 2) respect, 3) autonomy, 4) confidentiality, and 5) social support. Questions (i.e., questions Q119, Q122, Q125, Q129, and Q132) come from: “*WHO (2023). Patient-reported experiences in primary care: Patient questionnaire.*” Draft. (Personal communication from Dirk Horemans on April 18, 2023). **Important Note:** The field testing for these questions is not yet complete (this testing will probably be completed later this year).

**********************************************************************************

**2.a. KPI S2: “Provision of integrated supportive supervision”**

2.b. Sources: Slides 54–56 of the KPI Handbook, and row 25 of the IGS (indicator RSSH/PP HRH-5)

2.c. Definition

Percentage of facilities that implement supportive supervision defined by the following five attributes.

1) At least 1 supervision visit has occurred in the last period.
2) The last supervision visit covered integrated technical content.
3) Summary statistics related to program performance and quality were presented and discussed during last supervision visit.
4) The supervisor facilitated group problem-solving during the last supervision visit, based on a review of performance data.
5) The last supervision visit considered data on community-level activities provided by community health workers (CHWs).

Note: This definition has been somewhat revised and clarified (see Comments).

2.d. Measurement methodology

Measurement methods for each attribute are described below, as is an analysis method for summarizing the five attributes into a composite score. Also, see Table 2, below.

- **Attribute 1** (At least 1 supervision visit has occurred in the last period). This attribute can be measured by the HHFA indicator 4.3.1.4.1 (Permanent ID “CVU”; Percentage of facilities with a reported external supervision visit within the past three months). For details on the survey questions, see page 15 of the WHO HHFA Combined questionnaire (Availability, Readiness, and Management and finance), core and additional questions, version 2.0, June 2023.
- **Attribute 2** (The last supervision visit covered integrated technical content).
  - For facilities that provide services for HIV and TB and malaria, Attribute 2 means that the supervisor did at least one of the following six activities for HIV and TB and malaria (all three diseases). The six activities are: a) reviewing data (e.g., patient or lab registers, commodity stock records, community data, or summary statistics), b) observing clinical consultations or lab functions, c) giving feedback to HWs, d) answering HWs’ questions, e) providing on-the-spot training for HWs (e.g., on a new guideline, or how to perform a clinical task), or f) discussing community-based health activities. For example, the supervisor reviewed data for HIV (activity “a”), reviewed data for TB (activity “a”), and observed clinical consultations for malaria (activity “b”). That is, at least one supervisory activity that involved technical content for each of the three diseases (i.e., something done for all three diseases).
  - For facilities that do **not** provide services for HIV and TB and malaria, Attribute 2 means that the supervisor did at least one of the following six activities for at least three of the following health areas: HIV, TB, malaria, ANC, immunizations, or integrated management of childhood illness (IMCI)/child health. The six activities are: a) reviewing data (e.g., patient or lab registers, commodity stock records, community data, or summary statistics), b) observing clinical consultations or lab functions, c) giving feedback to HWs, d) answering HWs’ questions, e) providing on-the-spot training for HWs (e.g., on a new guideline, or how to perform a clinical task), or f) discussing community-based health activities. For example, if the facility provided services for malaria, ANC, and IMCI/child health, the supervisor reviewed data for malaria (activity “a”), observed clinical consultations for ANC (activity “b”), and gave feedback to HWs about IMCI/child health care (activity “c”). That is, at least one supervisory activity that involved technical content for each of the three health areas (i.e., something done for all three areas).
  - For facilities that provide services for fewer than three of the six health areas in the t-HFA questions (e.g., only HIV, or only HIV and TB), then Attribute 2 is not applicable and is assigned a missing value for the KPI S2 calculation (i.e., the concept of integration does not apply to facilities that do not provide services for multiple health areas).
- **Attribute 3** (Summary statistics related to program performance and quality were presented and discussed during last supervision visit). This attribute includes the presentation and discussion of data on any health area from any of the following sources: patient or lab registers, commodity stock records, community data or community activities, or summary statistics. The supervisor does not have to give the presentation; the supervisor only needs to participate in the activity.
- **Attribute 4** (The supervisor facilitated group problem-solving during the last supervision visit, based on a review of performance data). This attribute means any kind of formal or informal problem-solving activity that involves at least two facility staff members. The activity includes a review of any kind of health data (e.g., as in Attribute 3: patient or lab registers, commodity stock records, community data or community activities, or summary statistics).
- **Attribute 5** (The last supervision visit considered data on community-level activities provided by CHWs). This attribute means that the supervisor reviewed and discussed data on community-level activities provided by CHWs. This could include a simple discussion about which CHW-related community-level activities had occurred in the preceding period (typically 3 months).

Table 2. Indicators and survey questions on integrated supportive supervision

| **Indicator attribute** | **HHFA indicator** | **t-HFA survey questions** |
| --- | --- | --- |
| Attribute 1. Supervision occurred in the last period | - Indicator 4.3.1.4.1 (Permanent ID “CVU”; Percentage of facilities with a reported external supervision visit within the past three months). | 610 and 611 |
| Attribute 2. Last supervision visit covered integrated content | - No HHFA indicator. Questions developed by GF the t-HFA team. | C001A–C001F, C002A–C002F, C003A–C003F, C004A–C004F, C005A–C005F, and C006A–C006F |
| Attribute 3. Summary statistics discussed during last visit | - No HHFA indicator. Questions developed by the GF t-HFA team. | C007 |
| Attribute 4. Group problem solving during last visit | - No HHFA indicator. Questions developed by the GF t-HFA team. | C008 and C009 |
| Attribute 5. Data on community activities discussed during last visit | - No HHFA indicator. Questions developed by the GF t-HFA team. | C010 and C011 |

**Analysis method**

This method gives a score from 0 to 100 for each facility (a higher score means better quality supervision). For attribute 1, if there was no supervision visit in the last period, then the indicator score = 0. Each of the other four attributes is scored as a 100 (facility has the supervision attribute) or else 0. Each facility is scored as follows. If the facility supports and/or supervises CHWs that are linked to the facility, then the score = the sum of four attribute scores/4 (i.e., attributes 2–5 are considered). If the facility does **not** have CHWs linked to the facility, then the score = the sum of three attribute scores/3 (i.e., Attribute 5 is not applicable, so only consider the remaining three attributes 2, 3, and 4). Attributes with missing values are excluded from the numerator and denominator of the score calculation.

2.e. Comments

- The original indicator definition included a sixth attribute: the supervisor reported receiving supervision themselves. This attribute was removed because of concerns that: i) the measurement of the attribute would complicate the survey methodology (i.e., it required a telephone call to the facility’s supervisor), and ii) the measurement was susceptible to bias (i.e., compared to urban areas, supervisors from rural areas might be less likely to respond to the telephone call).
- Regarding the indicator definition, there was a key difference between the IGS (i.e., supervision was defined by meeting **all** five attributes) and the KPI Handbook (i.e., KPI is based on an average of scores across questions with available answers”—i.e., not that “all” key attributes are present). A choice was made to align with the KPI Handbook definition because it is more sensitive to change. For example, if a country (at baseline) met few attributes but (over time) met 4 of the 5 attributes consistently, that country’s score would not increase with the IGS definition but would increase with the KPI Handbook definition. GF and countries could also analyze this indicator with both definitions to obtain different perspectives on improvement.

**********************************************************************************

**3.a. KPI S3: “HTM integrated services offered to pregnant women”**

3.b. Sources: Slides 57–59 of the KPI Handbook, and row 5 of the IGS (indicator RSSH O-1)

3.c. Definition

Pregnant women receive the following services in an integrated fashion: HIV counseling and testing, access to ARVs for HIV positive women, IPTp, and TB screening.

Note: This definition has been slightly revised (see Comments).

3.d. Measurement methodology

- Use HHFA record review methods. Sample records for 5 ANC clients per facility for the HIV and IPTp indicators—for details, see page 13 of the WHO 2023 HHFA Module 3 (QoC). These methods cover HIV and IPTp, but not TB. Also, see Table 3, below.
- For TB, during the ANC record review, surveyors will abstract data to determine if, at any ANC visit, the pregnant woman is asked each of the following (i.e., four yes/no questions): a) current cough, b) fever, c) night sweats, and d) weight loss. These questions came from WHO's 2016 ANC guidelines: Recommendation B.1.8 on page 52 of "*WHO recommendations on antenatal care for a positive pregnancy experience*" (<https://www.who.int/publications/i/item/9789241549912>). Use of these four questions was approved by the GF TB Team. Note that this recommendation on TB screening with the 4 questions is also in ANC guidelines for South Africa (from 2015) and Tanzania (from 2018).
- Additional details
  - HIV counseling and testing. The HHFA indicator 5.1.1.1.10 (Permanent ID "CIA"; Percentage of ANC clients with counselling and testing for HIV received during ANC) was **not** used because the wording of HHFA is confusing (it conflates “offering” a test and “receiving” a test). In consultation with the GF HIV Team, a clearer question was created: “Is there documentation at any visit that the client received an HIV test during ANC?” Also note that HHFA question 13009_01 (“Was the client on ART prior to attending ANC?”) was added before the HIV testing question because women who are already on ART do not need HIV testing.
  - Access to ART for HIV positive women. This is measured by two questions developed by the GF t-HFA team: “Is there documentation that the client is HIV positive (diagnosed either during or prior to her pregnancy)?” and “For HIV-positive women, is there documentation that the woman is on ART during pregnancy/breastfeeding?”
  - IPTp. See methods for HHFA indicator "Percentage of ANC clients with IPT x 1 dose" (Permanent ID "DBA"; 5.1.1.2.4).
  - TB screening. Data on use of 4-question screening (current cough, fever, night sweats, and weight loss) will be abstracted from ANC records.

3.e. Comments

- Regarding the indicator definition, the IGS and KPI Handbook had definitions that were similar but not identical^[1]^. Therefore, elements from both were combined into a single definition.

Table 3. Indicators and survey questions on HTM integrated services offered to pregnant women

| **Attribute** | **HHFA indicator** | **t-HFA survey questions** |
| --- | --- | --- |
| Attribute 1. HIV counselling and testing | - A question developed by the GF t-HFA team was used, which is similar to, but not the same as, the HHFA question used to measure HHFA indicator 5.1.1.1.10 (Permanent ID "CIA"; Percentage of ANC clients with counselling and testing for HIV received during ANC). See explanation in section 3.d, above. | F003 and 13009_01 |
| Attribute 2. Access to ART for HIV-positive women | - No HHFA indicator. This attribute is measured with two questions developed by the GF t-HFA team. | F001 and F004 |
| Attribute 3. IPTp given at ANC | - Indicator 5.1.1.2.4 (Permanent ID "DBA"; "Percentage of ANC clients with IPT x 1 dose") | 13007_03_1 to 13007_03_5 |
| Attribute 4. TB screening | - No HHFA indicator. This attribute is measured with questions developed by the GF t-HFA team (based on WHO’s ANC guidelines). | F002_1_A, F002_1_B, F002_1_C, F002_1_D, F002_2_A, F002_2_B, F002_2_C, F002_2_D, F002_3_A, F002_3_B, F002_3_C, F002_3_D, F002_4_A, F002_4_B, F002_4_C, F002_4_D, F002_5_A, F002_5_B, F002_5_C, and F002_5_D |

**Analysis method**

First, for each of the four attributes, all elements must be present to get “credit” for the attribute (e.g., for the TB screening attribute, responses to all four TB screening questions must be documented). Second, for question 13009_01 (“Was the client on ART prior to attending ANC?”), if the response is yes, then remove it from the calculation because HIV testing is not needed for women who are already on ART (as they clearly had been tested in the past, before the current pregnancy). Third, for the overall indicator score for one ANC client, a continuous value (from 0 to 100) will be calculated for each ANC client that equals the percentage of attributes that are present multiplied by 100. If the response for an attribute is “don’t know” (i.e., missing information) or not applicable, it is excluded from the calculation. For example, if attributes 1 and 2 were present for an ANC client (and there were no “don’t know” responses and no “not applicable” responses), then the ANC client’s score would be 50 (i.e., 2/4 x 100). The facility-level score will be a simple, unweighted average of the individual client-level scores for ANC clients whose records were abstracted during the t-HFA visit.

**********************************************************************************

**4.a. KPI S5: “Systems readiness for CHWs”**

4.b. Sources: Slides 63–65 of the KPI Handbook, and row 3 of the IGS (indicator RSSH/PP O-1)

4.c. Definition

Percentage of CHWs with the following attributes:

1) Received integrated supportive supervision during the period
2) Have a contract specifying their scope of work, expected full-time equivalent (FTE) or expected hours per month/week/day, a level of financial remuneration that does not fall below the national minimum wage (pro-rated to their expected %FTE), timing of financial remuneration (e.g., monthly), rest days, annual leave, paid sick leave, holidays, and health insurance
3) Paid per their contract (all three of the following: amount, frequency, timeliness) during the period
4) Had no stockouts of commodities, equipment or job aids (e.g., registers, reporting forms per country norms) needed for their work during the period (i.e., all three supports were available)

4.d. Measurement methodology

- Sampling CHWs. The KPI Handbook states that “the data on CHWs will be collected via an interview with facility in-charge and/or record review of CHW related activities”, and the IGS states that “data will be collected from 5 CHWs associated with each sampled facility.” Therefore, the t-HFA survey team should contact the facility at least one week before the facility visit and do the following: 1) ask how many “RECO site” CHWs (see details on indicator HRH-4, below) are associated with the facility, and record their names on an enumerated list; 2) randomly select 5 RECO site CHWs from the list; and 3) ask the facility staff to invite the 5 randomly selected CHWs to come the facility on the day of the HFA visit for an interview. As the CHWs are likely employed by SANRU (an NGO), the survey team will contact SANRU (or any other organization that manages RECO site CHWs) and perform the above three steps. To determine which organization employs CHWs in a given facility catchment area, before the field work begins, the survey team should contact SANRU, the CCM, MoH, PRs and community PRs, and SRs. If a given facility has a combination of SANRU-employed CHWs and government-supported CHWs, then a harmonized list that includes all RECO site CHWs should be made, and 5 CHWs should be randomly selected from that harmonized list (sampling frame).
- If on the day of the HFA visit, there is at least 1 CHW that consents to participate in the survey, then the information from the CHW(s) will be collected via an interview. If there are no CHWs that are present and that give consent, then the information will be collected from the facility in-charge or other facility or SANRU staff member that interacts with the CHWs. Surveyors will record the data source (i.e., interview with CHWs or non-CHW staff person, such as the facility in-charge or SANRU staff).
- See Table 4 for details on questionnaire items.

- **Attribute 1.** Received integrated supportive supervision during the period.
  - Ask what the CHW’s responsibilities are: HIV, TB, malaria, nutrition, and MNCH.
  - Ask if the CHW received a supervision visit during the last completed calendar quarter. E.g., a survey conducted on August 1 would ask about supervision during the preceding April–June quarter. As monthly supervision is a best practice, this attribute will be measured with six questions: a) did the CHW receive a supervision visit during month 1 of the preceding quarter? (yes/no); b) if yes to “a”, which health areas did the supervision cover?; c) did the CHW receive a supervision visit during month 2 of the preceding quarter? (yes/no); d) if yes to “c”, which health areas did the supervision cover?; e) did the CHW receive a supervision visit during month 3 of the preceding quarter? (yes/no); and f) if yes to “e”, which health areas did the supervision cover?
  - Integrated supervision is defined below.
    - If the CHW is responsible for HIV and TB and malaria, then integrated supervision must address all three diseases during the same supervisory visit at least once in the past quarter.
    - If the CHW is not responsible for all three diseases, then were at least two health areas discussed (e.g., HIV and TB, or malaria and nutrition, etc.) during the same supervisory visit at least once in the past quarter?
    - Note. If a CHW receives supervision for a health area that she/he is not responsible for, then supervision on that health area does not contribute to integrated supervision (because such supervision would not help improve quality for the health areas that the CHW is responsible for).
- **Attribute 2.** Have a contract specifying their scope of work, expected full-time equivalent (FTE) or expected hours per month/week/day, a level of financial remuneration that does not fall below the national minimum wage (pro-rated to their expected % FTE), timing of financial remuneration (e.g., monthly), rest days, annual leave, paid sick leave, holidays, and health insurance. Yes/no questions for each of these elements.
- **Attribute 3.** Paid per their contract (amount, frequency, timeliness) during the period. Yes/no questions for each of these elements. Note that the CHW must have been paid on time and in-full (as per their contract) every month during the reporting period (typically, the past 3 months). This attribute is the same as indicator RSSH/PP HRH-3.
- **Attribute 4.** Commodities, equipment, and job aids (e.g., registers, reporting forms per country norms) needed for their work during the period were always available (and functioning properly, for equipment) during the reporting period (typically, the past 3 months). Yes/no questions for each element.

Table 4. Indicators and survey questions on system readiness for CHWs

| **Attribute** | **HHFA indicator** | **t-HFA survey questions** |
| --- | --- | --- |
| Attribute 1. Integrated supportive supervision | - There is no HHFA indicator. Questions developed by the GF t-HFA team. | E003A-E003E, E004, E005A-E005E, E006, E007A-E007E, E008, and E009A-E009E |
| Attribute 2.  CHW contract | - There is no HHFA indicator. Questions developed by the GF t-HFA team. | E010-E019 |
| Attribute 3. Remuneration | - There is no HHFA indicator. Questions developed by the GF t-HFA team. This attribute is the same as indicator RSSH/PP HRH-3. | E020-E023 |
| Attribute 4. Commodities, equipment, and job aids always available | - There is no HHFA indicator. Questions developed by the GF t-HFA team. | E024-E029 |

**Analysis method**

The IGS states that all four attributes must be present. However, the KPI Handbook states that: “The KPI is based on an average of scores across questions with available answers”—i.e., not that “all” key attributes are present. As a compromise, the following was developed. First, for each of the four attributes, all elements must be present to get “credit” for the attribute (e.g., all elements of a contract, for attribute 2). Second, for the overall indicator score, a continuous value (from 0 to 100) will be calculated for each CHW that equals the percentage of attributes that are present multiplied by 100. Third, if the response for a question about an attribute (e.g., question E010 on whether the CHW has a contract) is “don’t know”, then the response will be analyzed as if it was a “no”. The reason is that if a CHW does not know or is not sure whether (for example) he/she has a contract, then from a practical perspective it is as if the CHW does not have a contract. Here is an example calculation of the readiness score for an individual CHW: if attributes 1 and 2 were present for a CHW, then the readiness score would be 50 (i.e., 2/4 x 100). The reason for this approach is that it is more sensitive to change. For example, if a country (at baseline) met few criteria for CHW readiness, but (one year later) it met three of the four criteria consistently, that country’s score would not increase with the IGS definition but would increase with the KPI Handbook definition. GF and countries could also analyze this indicator with both definitions to obtain different perspectives on improvement. The KPI S5 indicator at the facility level is the average value of the individual readiness scores for the CHWs interviewed for the facility. For example, if the five CHW readiness scores for a facility are 50, 25, 75, 100, and 75, then the facility-level indicator value would be 65 (i.e., [50 + 25 + 75 + 100 + 75]/5).

************************************************************************************

**SECTION B. NON-KPI INDICATORS**

**5.a. RSSH/PP HRH-6: “Percentage of facilities providing effective services. Composite facility level indicator” with 7 attributes**

5.b. Sources: Row 26 of the IGS

5.c. Definition

Composite facility level indicator with seven attributes:

1) % of facilities observed to provide integrated services at ANC (TB, malaria, HIV) at the time of visit;
2) Provider availability (absence rate on day of visit);
3) Provider caseload (number of outpatient visits per clinician per day);
4) ANC dropout rate;
5) DPT dropout rate;
6) Treatment completion rate for new TB cases;
7) Twelve-month retention on ART

5.d. Measurement methodology

Measurement methods for each attribute are described below, as well as an analysis method for summarizing the seven attributes into a composite score. For details on questions in the t-HFA survey instrument, see Table 5, below.

1) **Attribute 1** (% of facilities providing integrated HTM services at ANC). First, ask if the facility provides ANC services. If yes, then measure this attribute with the result from indicator KPI S3. Note that Attribute 1 cannot be fully measured by the HHFA (see description of KPI S3, above).
2) **Attribute 2** (provider absence rate). This is the percentage of HWs expected to work on the day of the HFA visit (at the time of the visit) who are observed as absent. Note that Attribute 2 cannot be measured by the HHFA.
3) **Attribute 3** (caseload—number of outpatient visits/clinician/day). This is the average of the number of outpatient visits per clinician on the day of the HFA visit during usual working hours. It will be measured by dividing the number of outpatients seen at the facility by the end of working day by the number of HWs observed as present (see Attribute 2). Note that Attribute 3 cannot be measured by the HHFA.
4) **Attribute 4** (ANC dropout rate). First, ask if the facility provides ANC services. If yes, then randomly select records of five women seen at ANC who had been at least 32 or more weeks pregnant during the most recent visit. For details on sampling, see page 13 of WHO’s 2023 HHFA Module 3 (QoC). For each record, determine if the woman had had at least the minimum number of recommended ANC visits according to the country’s guideline (often four or eight visits). The ANC dropout rate is the proportion of women who did not have at least the minimum number of recommended ANC visits. Note that Attribute 4 can be measured by HHFA indicator 5.1.1.1.2 (permanent ID "DAN"; Percentage of ANC clients with at least four ANC contacts during the pregnancy), or indicator 5.1.1.1.3 (permanent ID "BBM"; Percentage of ANC clients with at least eight ANC contacts during the pregnancy).
5) **Attribute 5** (DPT dropout rate). First, ask if the facility provides immunization services. If yes, then measure this attribute, which is the dropout rate between infants receiving the first dose of DPT (DPT1) and the third dose (DPT3). The DPT1 to DPT3 dropout rate = ([DPT1 – DPT3] / DPT1) x 100%, where DPT1 is the number of infants receiving the first DPT dose in the past year, and DPT3 is the number receiving the third dose in the past year. Surveyors can examine facility immunization tally sheets over the preceding 12 months to obtain DPT1 and DPT3. Note that Attribute 5 cannot be measured by the HHFA.
6) **Attribute 6** (treatment completion rate for new TB clients). First, ask if the facility provides TB treatment services. If yes, then randomly select records of five TB clients. For details on sampling, see page 43 of WHO’s 2023 HHFA Module 3 (QoC). For each record, determine if the client had completed his/her TB treatment. Attribute 6 is the number of TB clients at the facility who completed treatment divided by the TB cases at the facility that were sampled. Note that Attribute 6 can be measured by HHFA indicator 5.4.1.1.12 (Permanent ID "AVN"; % of TB clients with successful treatment, either documented as cured or completed treatment at the end of treatment).
7) **Attribute 7** (12-month retention on ART). Ideally, this attribute would be assessed by determining if people living with HIV (PLHIV) who had started ART at least 12 months ago had been on ART continuously (i.e., for every month) in the 12 months before the facility HFA visit. However, HIV experts have advised that this would be challenging to measure in the context of a relatively rapid HFA. Thus, the following approach was adopted, which is aligned with the WHO indicator “ART.2 Total attrition from ART (Updated)” (source: page 327, *Consolidated guidelines on person-centred HIV strategic information: strengthening routine data for impact*. WHO, 2022; available at: https://www.who.int/publications/i/item/9789240055315). First, ask if the facility provides HIV treatment services. If yes, then randomly select records of five PLHIV who had started ART at least 6 months ago (as per the HHFA sampling methodology). For each record: 1) determine if the patient had started ART at least 12 months ago; and 2) for patients who had started ART at least 12 months ago, determine if the patient has been on ART at two time points at least 12 months apart. For example, if the survey visit was on August 1, 2023, and there was documentation that the patient was on ART in August 2022 and July 2023, then the criteria for Attribute 7 would be met. Note that if there is clear documentation that the patient had stopped ART during this time period, then the criteria for Attribute 7 would not be met. Attribute 7 is the number of PLHIV retained on ART over the previous 12 months divided by the number of PLHIV who started ART at least 12 months ago. Note that Attribute 7 cannot be measured by the HHFA.

Table 5. Indicators and survey questions on effective services

| **Attribute** | **HHFA indicator** | **t-HFA survey questions** |
| --- | --- | --- |
| Attribute 1. % of facilities providing integrated HTM services at ANC | - There is no HHFA indicator. This attribute will be measured by GF KPI S3. | See Table 3 |
| Attribute 2. Provider absence rate | - There is no HHFA indicator. Questions developed by the GF t-HFA team. | B025 and B026 |
| Attribute 3. Caseload | - There is no HHFA indicator. Questions developed by the GF t-HFA team. | B026 and B034 |
| Attribute 4.  ANC dropout rate | - Indicator 5.1.1.1.2 (Permanent ID “DAN”; Percentage of ANC clients with at least four ANC contacts during the pregnancy) - Indicator 5.1.1.1.3 (permanent ID "BBM"; Percentage of ANC clients with at least eight ANC contacts during the pregnancy) | 13004_07 |
| Attribute 5.  DPT dropout rate | - There is no HHFA indicator. Questions developed by the GF t-HFA team. | B032 and B033 |
| Attribute 6. Treatment completion rate for new TB clients | - Indicator 5.4.1.1.12 (Permanent ID "AVN"; % of TB clients with successful treatment, either documented as cured or completed treatment at the end of treatment) | 13506_4  . |
| Attribute 7.  12-month retention on ART | - There is no HHFA indicator. Questions developed by the GF t-HFA team. - Did the client begin ART at least 12 months ago? - If yes, has the patient been on ART at two time points at least 12 months apart?? | G002 and G003 |

**Analysis method**

To account for the differing number of attributes that apply to a given facility (e.g., not all facilities provide ANC, HIV, and TB care), the following approach will be used. Each attribute is scored on a scale of 0–100. (So, if an attribute is calculated as a percentage, for the purpose of this analysis, treat the percentage as a number between 0 and 100.) The indicator value for a specific facility equals the sum of attribute scores divided by the number of attributes that are relevant for the facility, based on the services that it delivers.

1) **Attribute 1** (% of facilities providing integrated HTM services at ANC) is 100 if the facility provides integrated HTM services at ANC, or 0 if not.
2) **Attribute 2** (provider absence rate) is 1 – the provider absence proportion (expressed as a number between 0 and 1) x 100.
3) **Attribute 3** (caseload—number of outpatient visits/clinician/day) is scored by multiplying the mean number of outpatient visits/clinician/day x 5. If the mean is greater than 20 patients/clinician/day, then the score is capped at a maximum of 100.
4) **Attribute 4** (ANC dropout rate) is scored as the ANC completion proportion (or “rate”) x 100. I.e., if one expresses the ANC dropout rate as a proportion between 0 and 1, then the ANC completion proportion is the complement (i.e., 1 – ANC dropout proportion).
5) **Attribute 5** (DTP dropout rate) is scored as the DPT completion proportion (or “rate”) x 100. I.e., the DPT completion proportion is the complement of the dropout rate, or DPT3/DPT1 (expressed as proportion between 0 and 1). If the completion proportion is greater than one (e.g., due to data errors or population shifts that allowed for more DPT3 doses given in the previous year than DPT1 doses), then the completion proportion is capped at a maximum of one.
6) **Attribute 6** (treatment completion rate for new TB clients) is scored as the completion proportion (expressed as a number between 0 and 1) x 100.
7) **Attribute 7** (12-month retention on ART) is scored as the retention proportion (expressed as a number between 0 and 1) x 100.

For example, if facility Y does not provide HIV and TB treatment services, then attributes 6 and 7 would not apply. If facility Y had scores of 0, 75, 50, 80, and 85, for attributes 1–5, respectively, then the facilities indicator score would be 58 (i.e., 0 + 75 + 50 + 80 + 85 divided by 5 attributes, or 290/5).

**********************************************************************************

**6.a. RSSH/PP HRH-3: “Percentage of community health workers remunerated on time and in-full (as per their contract) every month during the reporting period”**

6.b. Sources: Row 23 of the IGS

6.c. Definition

- Numerator = Number of CHWs remunerated on time and in-full (as per their contract) every month during the reporting period.
- Denominator = Total number of CHWs expected to be remunerated every month during the reporting period.

6.d. Measurement methodology

This indicator is the same as Attribute 3 of KPI S5 (Systems readiness for CHWs). Data collection will use the same method as used for KPI S5.

**********************************************************************************

**7.a. CSS-3: “Percentage of health service delivery sites with a community-led monitoring mechanism in place”**

7.b. Source: Row 36 of the IGS

7.c. Definition

- Numerator = Number of health service delivery sites with a community-led monitoring mechanism in place.
- Denominator = Total number of health service delivery sites in the administrative area.
- Data source: Targeted HFA / LFA spot checks / CLM reporting by PR
- Comment in column P (Analysis and interpretation) of IGS. Having a community-led monitoring mechanism in place at health service delivery sites, depending on the mechanism or the tool used, is defined as:
  - When trained community data collectors systematically and routinely collect information on the availability, accessibility, acceptability, quality of HIV, TB or malaria services, and provide feedback to the health facilities/sites on (at least) a quarterly basis.
  - When health care staff and/or administration of a health service delivery site are sensitized and made aware of the community-led monitoring mechanism/tool in place with the purpose of informing the clients of its existence and promoting its use.

7.d. Measurement methodology

This indicator has six attributes. The following six questions (B021 to B024, B035, and B045) are asked of the facility staff member who is most knowledgeable of community-level health activities. The questions ask about parts of the CLM definition, above. The facility gets a score on a 0–100 scale that equals the percentage of the six questions with a “strongly agree” or “agree” response (e.g., a score of 16.7 [i.e., 1/6] if one of the six questions has a strongly agree or agree response, a score of 33.3 if two of the six questions have a strongly agree or agree response [i.e., 2/6], and so on). Any questions not asked because of a skip pattern get a score of zero. This is because the element of the CLM definition is absent — even if the question is not asked. The skip patterns omit questions that would be obvious or illogical to ask given the response to the preceding "gateway" question. For example, if staff at the facility are unaware of the CLM mechanism (question B023), it would be illogical to ask if staff inform clients about the CLM mechanism (question B024). The denominator is always 6 for this indicator.

1) Clients that use this facility provide feedback on the quality of services at this facility. [responses: strongly agree/agree/neither agree nor disagree/disagree/strongly disagree/don’t know]
2) The community that this facility serves has trained community data collectors that routinely collect information on the availability, accessibility, acceptability, and quality of HIV, TB, or malaria services. [responses: strongly agree/agree/neither agree nor disagree/disagree/strongly disagree/don’t know]
   - IF STRONGLY AGREE OR AGREE, ASK:
3) The community provides feedback to this facility on (at least) a quarterly basis. [responses: strongly agree/agree/neither agree nor disagree/disagree/strongly disagree/don’t know]
4) This facility routinely analyzes and uses the community-led monitoring data for quality improvement. [responses: strongly agree/agree/neither agree nor disagree/disagree/strongly disagree/don’t know]
5) Health care staff at this facility are aware of the community-led monitoring mechanism. [responses: strongly agree/agree/neither agree nor disagree/disagree/strongly disagree/don’t know]
   - IF STRONGLY AGREE OR AGREE, ASK:
6) Health care staff inform the clients at this facility of the existence of the community-led monitoring mechanism and promote its use. [responses: strongly agree/agree/neither agree nor disagree/disagree/strongly disagree/don’t know]

**********************************************************************************

**8.a. HSG-1.1: “Percentage of facilities with written and updated clinical guidelines for HIV, TB, malaria and/or PHC (based on the services provided) developed by the national or sub-national government (as appropriate by the country context)”**

8.b. Sources: Row 34 of the IGS

8.c. Definition

- Numerator = Healthcare facilities, supported by TGF grant, that provide HIV, TB, Malaria and/or PHC services which can present updated written guidelines for HIV, TB, Malaria and/or PHC (as relevant based on the services provided) developed by the national or sub-national government (as appropriate by the country context).
- Denominator = Total number of facilities, supported by TGF grant, that provide HIV, TB, Malaria and/or PHC services.
- Data source: T-HFA. This measures availability of clinical guidelines. It can be collected annually whether used or not.
- Note 1. For this indicator, “PHC” is operationally defined as these services: ANC, IMCI/child health, and/or immunizations.
- Note 2. The condition “supported by TGF grant” (in the IGS numerator and denominator) will not be used because the condition is: 1) not in the wording of the indicator, 2) not part of other RSSH indicators, and 3) difficult to operationalize in the field (e.g., countries might not have accurate lists of which facilities are “supported” by GF grants, and facility staff might not know).

8.d. Measurement methodology

- Direct observation by t-HFA surveyors and interviews with facility staff. The ideal method is direct observation of a guideline document by a surveyor. The secondary method is when a facility staff member reports that the guideline is present, even when it cannot be directly observed.
- Each surveyed country defines what is meant by “written updated clinical guidelines… developed by the national or sub-national government” for each clinical topic (see Table 6, below). The t-HFA has a gateway question that asks if the facility offers a given service that is associated with a guideline, as well as survey items on the guideline itself. E.g., Question 1400. Does this facility offer diagnosis and/or treatment of malaria? Question 1406 (which has several parts, but only the last part is the response entered by the survey). Are national guidelines for the diagnosis and/or treatment of malaria available in this service site today? [COUNTRY ADAPT – NAME OF DOCUMENT AND VERSION] IF YES, ASK: May I see the guidelines? There are three responses to this last part of the question: YES, OBSERVED; YES, REPORTED, NOT SEEN; and NO/NOT AVAILABLE.

Table 6. Indicators and survey questions on the presence of clinical guidelines

| **Attribute** | **HHFA indicator** | **t-HFA survey questions** |
| --- | --- | --- |
| HIV | - Indicator 3.2.4.2.1 (Permanent ID "BOG"; Percentage of facilities offering HIV testing with guidelines on HIV counselling and testing)* | 2300 and 2308_01 |
|  | - Indicator 3.2.5.2.1 (Permanent ID "BQP"; Percentage of facilities offering ART with guidelines for ART)* | 2312** and 2315B_01 |
| TB | - Indicator 3.2.3.3.1 (Permanent ID "BME"; Percentage of facilities offering TB services with guidelines for diagnosis and treatment of TB)* | 2400, 2419_01, and 2419_03 |
| Malaria | - Indicator 3.2.2.2.1 (Permanent ID "BKH"; Percentage of facilities offering malaria services with guidelines for diagnosis and treatment of malaria)* | 1400 and 1406 |
| PHC: ANC | - Indicator 3.1.2.2.1 (Permanent ID "AXK"; Percentage of facilities offering ANC services with national guidelines on ANC)* | 1810 and 1817_01 |
| PHC: immunizations | - Indicator 3.2.1.2.1 (Permanent ID "BHS"; Percentage of facilities offering immunization services with guidelines for child immunization)* | 2100 and 2119_01 |
| PHC: IMCI/ child health | - Indicator 3.1.8.2.1 (Permanent ID "BFI"; Percentage of facilities offering child health preventative and curative care services with guidelines for IMCI)* | 2000 and 2005_01 |

* As per the GF RSSH indicator definition, only national guidelines are measured.

** “Life-long” has been removed from the question, as per the recommendation of the GF HIV Team and in consultation with WHO.

**Analysis method**

To account for the differing number of guidelines that apply to a given facility (which depends on the services provided by the facility), the following approach will be used. For each health condition, the score is 100 if the guideline is present or 0 if not present. Note that for HIV, "HIV counseling and testing" and "ART" are considered separately (because not all facilities that do HIV counseling and testing also provide ART). The indicator value for a specific facility equals the sum of health condition-specific scores divided by the number of health conditions that are relevant for the facility, based on the services that it delivers. For example, if a facility provides services for four of the health conditions (HIV counseling and testing, malaria, ANC, and IMCI/child health) and guidelines are only available for malaria and ANC, then the score for the facility would equal 0 (for HIV counseling and testing) + 100 (for malaria) + 100 (for ANC) + 0 (for IMCI/child health) divided by 4, or 200/4 = 50.

**********************************************************************************

**9.a. RSSH/PP RCS-1: “Percentage of health facilities able to provide oxygen therapy related services among those providing the service”**

9.b. Sources: Row 42 of the IGS

9.c. Definition

- Numerator = Number of health facilities with functional oxygen systems.
- Denominator = Total number of health facilities (surveyed/in sample).
- Data source: Spot Check and t-HFA.
- Analysis and interpretation:
  - Oxygen source (PSA plant, Liquid Oxygen, Oxygen concentrators), Facility type (primary, secondary, tertiary)
  - This indicator is based on consensus list of indicators emerging from a WHO global consultation on key performance indicators needed to measure oxygen investments under the domains of 1) oxygen ecosystem, 2) procurement, 3) operational 4) patient impact. The current indicator is a measurement of the coverage of functional oxygen systems in the surveyed facilities. Services is a broad umbrella term that includes Oxygen source -> distribution -> delivery to patients -> oxygen use at point of care. Measuring services would involve several data points along that results chain. Hence, in the case of this indicator we would limit the term "services" to that of "functional oxygen system" in the facility to measure the availability (coverage) of functional oxygen systems in the sampled facilities.

9.d. Measurement methodology

- Direct observation by t-HFA surveyors and interviews with facility staff.
- See Table 7, below.

**Analysis method**

For a specific facility, the value of the indicator ranges between 0 and 100. The value is 100 if attribute 1 (availability of oxygen) and attribute 2 (availability of pulse oximetry) are both true (see details in Table 7). The value is 50 if only one of these two attributes is true, and the value is 0 if neither attribute is true.

Table 7. Indicators and associated survey questions on oxygen therapy

| **Attribute** | **HHFA indicator** | **t-HFA survey questions** |
| --- | --- | --- |
| Attribute 1. Oxygen availability and delivery equipment | - The attribute 1 indicator is the same as the numerator of HHFA indicator 2.4.4.2.1 (Permanent ID "AVL"; Percentage of facilities offering oxygen services with oxygen available). Thus, it either has a value of true or false. | 1323, 1324,  1325_04 (A & B), 1325_05 (A & B), 1325_06 (A & B),  and 1326 |
|  | - Additional equipment: centrally piped oxygen supply; oxygen concentrator; oxygen tank/cylinder with attached pressure gauge, pressure regulator. Note that these 3 data elements are not used in attribute 1 and not used to calculate t-HFA indicator RSSH/PP RCS-1. They should be used as complementary information. | 1325_01, 1325_02, and 1325_03 |
| Attribute 2. Pulse oximetry | - The attribute 2 indicator is the same as the numerator of HHFA indicator 2.4.4.2.3 (Permanent ID "AVO"; Percentage of facilities offering oxygen services with pulse oximeter). Thus, it either has a value of true or false. | 1322_09 (A & B) |

**********************************************************************************

**10.a. RSSH/PP LAB-5: “Percentage of health facilities that have an appropriate set of diagnostics for their healthcare facility level, based on adapted WHO model list of essential in vitro diagnostics (EDL 3)” (Important note on the definition: See section 10.c, below.)**

10.b. Sources: Row 41 of the IGS. “Adapted” was added to the definition based on a recommendation from the GF t-HFA team.

10.c. Definition

- Modification of the indicator definition.
- Original definition from the IGS. Numerator = Number of health facilities assessed to have the appropriate set of diagnostics available for the healthcare facility level. Denominator = Number of healthcare facilities with diagnostic sample-taking capability.
- Modified definition is used. The indicator is the mean percentage of diagnostic tests available at health facilities, based on an adapted WHO model list of essential in vitro diagnostics. For details, see the Analysis Method, below.
- Data source: T-HFA. Some countries may be implementing HF surveys; and if they do, not do a t-HFA but rather build on the national protocol.

10.d. Measurement methodology

T-HFA survey teams will ask about which diagnostic test methods are available at the facility. The t-HFA questions are: 2900, B001, and TEST1 to TEST28. Note that the original the t-HFA questionnaire had an instruction stating that the tests to be assessed will be based on country-specific essential diagnostic lists (EDLs). However, as it was not possible to obtain country-specific EDLs, a standard list of tests (based on WHO recommendations) was developed and used for all countries. See details below in the Analysis Method.

**Analysis method**

Rather than an all-or-nothing binary score at the facility level (as described in the original definition above), the calculation of this indicator for a specific facility is the percentage (i.e., a score between 0 and 100) of required diagnostic tests for that facility type that are found in the facility. This approach, which is aligned with other t-HFA indicators, allows for greater sensitivity in showing progress over time.

If the response for a question about a test is “don’t know”, then the response will be analyzed as if it was a “no”. The reason is that respondents are selected by surveyors because they are knowledgeable about lab tests at the facility; and if such respondents do not know whether the facility has a particular test, then from a practical perspective it is as if the facility does not have the test. The list of required diagnostic tests depends on whether the facility performs any tests and whether the facility has a laboratory (from question 2900). Here are the details.

- If the response to question 2900 is "YES, with laboratory in the facility", then calculate a score from 0–100 that corresponds to the percentage of 27 tests that the facility performs. The questions for these 27 tests are TEST1 to TEST27. Note that the test assessed by question TEST28 (i.e., Vibrio cholerae antigen) is not used in this calculation. For example, if a facility with a lab has 18 of the 27 tests (and no “don’t know” responses), then the facility’s score would be 67 (i.e., 18/27 x 100).
- If the response to question 2900 is "YES, without laboratory in the facility", then calculate a score from 0–100 that corresponds to the percentage of 11 tests that the facility performs. The questions for these 11 tests are TEST1 to TEST7, TEST19 to TEST21, and TEST28.
- If the response to question 2900 is "NO LABORATORY TESTS PERFORMED", then the facility’s score is 0 (i.e., the facility has no testing capability).

To calculate the indicator at the country level, all facility-specific scores are averaged together.

**********************************************************************************

**11.a. RSSH/PP HRH-1: "Vacancy rate: Ratio of unfilled posts to total number of posts"**

11.b. Source: Row 21 of the IGS.

11.c. Definition

- Numerator = Number of funded full-time posts that have not been filled for at least six months.
- Denominator = Total number of funded posts.
- Data source: Country HMIS. If the HMIS is not functioning well, then this indicator will be measured by the t-HFA.
- From column Q. Source: National Heath Workforce Accounts 2017, indicator 5-07; page 66.

<https://apps.who.int/iris/bitstream/handle/10665/259360/9789241513111-eng.pdf;jsessionid=1E615EBC16A15A949CDF8B3EBBB03AFE?sequence=1>

- Note: This indicator did not seem to be in the HHFA.

11.d. Measurement methodology

The t-HFA questions are the following.

- Question B046. What is the total number of funded full-time posts at this facility? That includes filled and unfilled posts.
- Question B047. How many funded full-time posts do you have at this facility that have not been filled for at least six months?
- Question B048. What is the number of funded full-time posts at this facility for **physicians**? That includes filled and unfilled posts.
- Question B049. How many funded full-time posts do you have at this facility for **physicians** that have not been filled for at least six months?
- Question B050.	What is the number of funded full-time posts at this facility for **nurses and midwives**? That includes filled and unfilled posts.
- Question B051.	How many funded full-time posts do you have at this facility for **nurses and midwives** that have not been filled for at least six months?
- Question B052.	What is the number of funded full-time posts at this facility for **lab technicians**? That includes filled and unfilled posts.
- Question B053.	How many funded full-time posts do you have at this facility for **lab technicians** that have not been filled for at least six months?
- Question B054.	What is the number of funded full-time posts at this facility for **pharmacists**? That includes filled and unfilled posts.
- Question B055.	How many funded full-time posts do you have at this facility for **pharmacists** that have not been filled for at least six months?
- Question B056. What is the number of funded full-time posts at this facility for **community health workers**? That includes filled and unfilled posts.
- Question B057. How many funded full-time posts do you have at this facility for **community health workers** that have not been filled for at least six months?

**********************************************************************************

**12.a. RSSH/PP HRH-4: “Percentage of community health workers providing high quality HIV, TB and malaria services.”**

12.b. Source: Row 24 of the IGS

12.c. Definition

- Modification of the indicator definition.
- Original definition from the IGS. Numerator = CHWs meeting 80% and above threshold for providing tracer HTM services according to protocol. Denominator = CHWs providing HTM services.
  - Modified definition that is used. The indicator is a mean of individual CHW scores that each range from 0–100. For details, see the Analysis Method, below. The primary reason for this modified definition is that this approach is more sensitive to change over time. Additionally, this is the approach used for most of the other t-HFA indicators.
- Data source: Abstracted from individual CHW consultation forms (from sample of CHWs attached to health facilities) assessed through targeted health facility assessment.

12.d. Measurement methodology

- In the Democratic Republic of the Congo (DRC), which is the only country currently requesting measurement of the HRH-4 indicator, there are two types of GF-supported CHWs that will be evaluated. The first CHW type treats ill children (in addition to other responsibilities) at the community level. In French, they are called “*Relais Communautaire site*” or “RECO site”. The second CHW type does not treat ill children; rather, they make household visits, provide health communication, and identify clients that need to be referred to a facility (e.g., women who deliver at home), in addition to other responsibilities. In French, they are called “Relais Communautaire Promotionnels” or “RECO PL”. In general terms, both CHW types will be evaluated using a record review. However, as the two CHWs types have different responsibilities and use different records to document their work, the attributes and measurement methods differ, too.
- **Measurement methodology for RECO site CHWs**
  - When treating clients, these CHWs use a paper register with each row being a place to record information for one client and each column corresponding to a different data element (e.g., client’s name, weight, temperature, symptoms, and treatments). There are 66 columns in total. The content of each column varies. For example:
    - In column 1 (“No. (#)”), the CHW records a number;
    - In column 2 (“Date”), the CHW records the date;
    - In column 4 (“Age”), the CHW records a number and letter for the client’s age (e.g., “4 a” means “4 *ans*”, which is French for 4 years; or “6 m” for “6 *mois*”, which is French for 6 months; or “5 j” for “5 *jours*”, which means 5 days); and
    - In column 9 (“*Toux/Rhume*”, which is French for “cough/cold”), the CHW records either an “X” (meaning no cough or cold) or a number, which is the number of days that the client has had cough or cold symptoms.
    - Additionally, some columns might be intentionally blank. For example, if column 9 has an “X” (meaning no cough or cold), then column 18 (“*Comptage mvt resp*”, which is a French abbreviation for “count a 60-second respiratory rate”) might be left blank because if a client has no respiratory illness, then the health worker does not need to count a respiratory rate.
  - The t-HFA survey team should invite 5 RECO site CHWs to each facility on the day of the t-HFA visit. For details on sampling CHWs, see t-HFA Annex 2, Section 5 (pages 22 and 27), which is based on page 13 of WHO’s 2023 HHFA Module 3 (Quality of care [QoC]). Note that these 5 RECO site CHWs will be the same CHWs recruited for the CHW interview (see indicator KPI S5, above). In DRC, the RECO site CHWs are supported by the non-governmental organization SANRU, so SANRU can assist with contacting and inviting CHWs to the facility on the day of the t-HFA survey visit. **It is important that RECO site CHWs bring their client registers for the record review. Without the client registers, no data can be collected on quality of care.**
  - **HIV quality of care.** For RECO site CHWs, given the documentation in the patient register (pictured above), there did not appear to be any indicators that could be measured for HIV quality of care.
  - **Attribute 1. TB quality of care.** For each enrolled RECO site CHW, randomly select 5 client records for record review for TB. Sampling instructions are in Annex 2, page 27. The only eligibility criterion for TB quality of care is that the client had a household exposure to a TB case patient — i.e., column 16 of the client register (“*Notion de Contage TB*”) has a check mark. If column 16 is blank or has an “X” or has anything written that is not a check mark, then the client record is ineligible. For each of the 5 selected client records, respond to questions EA06 and EA07 (for the purpose of explaining the analysis, below, the client-specific variable names are: EA06A, EA06B, EA06C, EA06D, and EA06E; and EA07A, EA07B, EA07C, EA07D, and EA07E).
  - **Attributes 2–7. Malaria quality of care.** For each enrolled RECO site CHW, randomly select 5 client records for record review for malaria. The only eligibility criterion for malaria quality of care is that the client had a febrile illness — i.e., column 11 of the client register (“*Fièvre (jrs)*”, which is French for “fever (days)”) has a number (which means the number of days that the client had fever). If column 11 is blank or has an ”X” or has anything written that is not a number, then the client record is ineligible. Here are the specific malaria quality of care attributes.
    - Attribute 2. Client with febrile illness is assessed for important signs and symptoms. For each of the 5 selected client records, respond to questions EA08 (cough), EA09 (diarrhea), EA10 (fever), and EA11 (signs indicating need for referral).
    - Attribute 3. Client with non-severe febrile illness is tested with a malaria rapid diagnostic test. For each of the 5 selected client records, respond to questions EA10 (fever), EA11 (signs indicating need for referral), and EA16 (malaria rapid diagnostic test [RDT] was done).
    - Attribute 4. Client with severe febrile illness is referred to a health facility. For each of the 5 selected client records, respond to questions EA10 (fever), EA11 (signs indicating need for referral), and EA25 (client referred).
    - Attribute 5. Client with non-severe febrile illness is correctly classified. For each of the 5 selected client records, respond to questions EA10 (fever), EA11 (signs indicating need for referral), EA17 (malaria classification), EA18 (non-malaria fever classification), and EA19 (suspected malaria case).
    - Attribute 6. Client with uncomplicated malaria is treated with artemisinin-based combination therapy (ACT). For each of the 5 selected client records, respond to questions EA10 (fever), EA11 (signs indicating need for referral), EA17 (malaria classification), EA19 (suspected malaria case), EA21 (treatment with Artemether-Amodiaquine [Art-AQ], young child dose), EA22 (treatment with Art-AQ, child dose), EA23 (treatment with Art-AQ, older child/adult dose), and EA24 (treatment with Artemether-Lumefantrine).
    - Attribute 7. Client with non-severe, non-malaria febrile illness is not treated with ACT. For each of the 5 selected client records, respond to questions EA10 (fever), EA11 (signs indicating need for referral), EA18 (non-malaria fever classification), EA21 (treatment with Art-AQ, young child dose), EA22 (treatment with Art-AQ, child dose), EA23 (treatment with Art-AQ, older child/adult dose), and EA24 (treatment with Artemether-Lumefantrine).
  - **Attributes 8–9. Pneumonia quality of care.**
    - Attribute 8. Client with non-severe febrile illness and cough is evaluated for pneumonia. For each of the 5 selected client records, respond to questions EA08 (cough), EA10 (fever), EA11 (signs indicating need for referral), EA12 (check respiratory rate), and EA13 (check for chest indrawing).
    - Attribute 9. Client with non-severe febrile illness and non-severe pneumonia is treated with an antibiotic. For each of the 5 selected client records, respond to questions EA10 (fever), EA11 (signs indicating need for referral), EA15 (pneumonia classification), and EA20 (treatment with amoxicillin).
- **Measurement methodology for RECO PL CHWs**
  - Each month, each RECO PL CHW summarizes the results of their household visits and other community contacts in a paper form. DRC colleagues have shared two similar documents with similar data elements. One is called the “*SNIS RDC / Rapport Mensuel du RECO*” (6 pages long, with a date of June 9, 2024), and the other is called the “*SNIS RDC/ Rapport Mensuel de CAC*” (3 pages long, with a date of January 3, 2023). See screen shots below of the tops of page 1 from both documents. These monthly forms are supposed to be stored at the facility to which the CHWs are linked. Thus, if these monthly forms can be found, it is not be necessary to invite the RECO PL CHWs to come to facilities on the day of the t-HFA visit. For each RECO PL linked to the facility, the t-HFA survey team should identify monthly forms for the 3 most recently completed months. For example, if the DRC facility visit occurs in December, monthly forms for September, October, and November should be sought.
  - Attribute 10. Measurement of the percentage of recommended household visits done. For the reporting forms of each of the 3 most recently completed months, respond to question EA28 (“*VAD/ Visite des menages réalisées*”, or households visited). For the purpose of describing the analysis, the variables for the 3 months are called EA28A, EA28B, and EA28C.
  - Attribute 11. Measurement of the percentage of households with a child under five years old that slept under a long-lasting insecticide-treated net (LLIN). For the reporting forms of each of the 3 most recently completed months, respond to questions EA29 ("*Ménages qui disposent la MIILD*", or number of households with a LLIN) and EA30 (“*Ménages ayant des enfants de moins de 5 ans qui dorment sous la MIILD*”, or number of households with a child under five years old that slept under an LLIN).
  - Attribute 12. Measurement of the percentage of women who deliver at home that are referred to a health center. For the reporting forms of each of the 3 most recently completed months, respond to questions EA31 ("*Femmes ayant accouché à domicile*", or number of women who gave birth at home) and EA32 (“*Femmes ayant accouché à domicile et orientées vers le CS*”, or number of women who gave birth at home that were referred to a health center).
  - Attribute 13. Percentage of children under 6 months old who are exclusively breastfed. For the reporting forms of each of the 3 most recently completed months, respond to questions EA33 ("*Enfants de 0 à 6 mois*”, or number of children under 6 months old) and EA34 (“*Enfants de 0 à 6 mois allaités exclusivement au sein*”, or number of children under 6 months old who are exclusively breastfed).

.

- See Table 8, below.

**Analysis method**

As described above, in DRC, there are two CHW types that will be evaluated for indicator HRH-4: RECO site CHWs and RECO PL CHWs. Ideally, there will be data from 5 RECO site CHWs and 5 RECO PL CHWs per facility. For each of these (ideally) 10 CHWs, calculate a score from 0–100 (a higher score means better quality), which is based on the CHW’s performance for the 13 attributes. (The attribute-specific methods are described below.) The overall HRH-4 indicator value for a facility equals the unweighted mean of the 10 CHW-specific scores. For example, if 5 RECO site CHWs have scores of 20, 50, 50, 80, and 100, and if 5 RECO PL CHWs have scores of 40, 60, 100, 100, and 100, then the HRH-4 indicator value for the facility would be the average of these 10 values — i.e., (700)/10, or 70. With this approach, one can calculate a mean score (from 0–100) for an individual facility, group of facilities (e.g., in a geographic province, or a facility type), entire country, or group of countries. To calculate the indicator at the country level, all facility-specific scores are averaged together.

Here are calculation details for the attributes. Note that Attributes 1–9 only apply to RECO site CHWs, and Attributes 10–13 only apply to RECO PL CHWs.

*Analysis method for RECO site CHWs*

To calculate the score of an individual CHW, consider the calculation as a proportion from 0–1, which at the end is multiplied by 100 to get a score from 0–100. As each CHW score is a proportion (for calculation purposes), the methods below described in terms of adding a point to a CHW’s numerator and denominator. For example, with Attribute 1, each client record has a score of either 0 (task not done) or 1 (task done). If there are 5 client records for a given CHW, then each client record contributes 1 point to the CHW’s denominator, and (hypothetically) the Attribute 1 task is done for 3 client records, then 3 points are given to the CHW’s numerator. Hence (for this simple Attribute 1 example), the CHW’s proportion would be 3/5 or 0.6.

- **Attribute 1.** Client with household contact with a TB case patient is referred. For each client record with a “YES” response to question EA06 (i.e., the 5 client-specific variables are EA06A to EA06E) (i.e., client had a household contact with a TB case patient), increase the CHW’s denominator by 1 point. For each client record that indicates that the client had a TB contact (e.g., EA06A = “YES”) **AND** that the client is referred (using responses to question EA07 — e.g., EA07A = “YES”), then increase the CHW’s numerator by 1 point.
- **Attribute 2.** Client with febrile illness is assessed for important signs and symptoms. For each client record with a “1” response to question EA10 (client had fever), increase the CHW’s denominator by 1 point. For each client record with a “1” response to question EA10 **AND** a “1” or “2” response to question EA08 (patient checked for cough/cold) **AND** a “1” or “2” response to question EA09 (patient checked for diarrhea) **AND** a “1” or “2” response to question EA11 (patient checked for signs indicating need for referral), increase the CHW’s numerator by 1 point.
- **Attribute 3.** Client with non-severe febrile illness is tested with a malaria rapid diagnostic test. For each client record with a “1” response to question EA10 (client had fever) **AND** a “2” response to question EA11 (no signs indicating need for referral), increase the CHW’s denominator by 1 point. For each client record with a “1” response to question EA10 (client had fever) **AND** a “2” response to question EA11 (no signs indicating need for referral) **AND** a “1” response to question EA16 (RDT done), increase the CHW’s numerator by 1 point.
- **Attribute 4.** Client with severe febrile illness is referred to a health facility. For each client record with a “1” response to question EA10 (client had fever) **AND** a “1” response to question EA11 (client had signs indicating need for referral), increase the CHW’s denominator by 1 point. For each client record with a “1” response to question EA10 (client had fever) **AND** a “1” response to question EA11 (client had signs indicating need for referral) **AND** a “1” response to question EA25 (client referred), increase the CHW’s numerator by 1 point.
- **Attribute 5.** Client with non-severe febrile illness is correctly classified. For each client record with a “1” response to question EA10 (client had fever) **AND** a “2” response to question EA11 (no signs indicating need for referral), increase the CHW’s denominator by 1 point. For each client record with a “1” response to question EA10 (client had fever) **AND** a “2” response to question EA11 (no signs indicating need for referral) **AND** a “1” response to question EA16 (RDT done) **AND** a “1” response to either question EA17 (malaria classification) or EA18 (non-malaria fever classification), increase the CHW’s numerator by 1 point. For each client record with a “1” response to question EA10 (client had fever) **AND** a “2” response to question EA11 (no signs indicating need for referral) **AND** a “2” response to question EA16 (RDT not done) **AND** a “1” response to question EA19 (suspected malaria case), increase the CHW’s numerator by 1 point.
- **Attribute 6.** Client with uncomplicated malaria is treated with ACT. For each client record with a “1” response to question EA10 (client had fever) **AND** a “2” response to question EA11 (no signs indicating need for referral) **AND** a “1” response to either question EA17 (malaria classification) or EA19 (suspected malaria case), increase the CHW’s denominator by 1 point. For each client record with a “1” response to question EA10 (client had fever) **AND** a “2” response to question EA11 (no signs indicating need for referral) **AND** a “1” response to either question EA17 (malaria classification) or EA19 (suspected malaria case) **AND** a “1” response to any of the four ACT treatments [i.e., EA21 (treatment with Art-AQ, young child dose), EA22 (treatment with Art-AQ, child dose), EA23 (treatment with Art-AQ, older child/adult dose), or EA24 (treatment with Artemether-Lumefantrine)], increase the CHW’s numerator by 1 point.
- **Attribute 7.** Client with non-severe, non-malaria febrile illness is not treated with ACT. For each client record with a “1” response to question EA10 (client had fever) **AND** a “2” response to question EA11 (no signs indicating need for referral) **AND** a “1” response to question EA18 (non-malaria fever classification), increase the CHW’s denominator by 1 point. For each client record with a “1” response to question EA10 (client had fever) **AND** a “2” response to question EA11 (no signs indicating need for referral) **AND** a “1” response to question EA18 (non-malaria fever classification) **AND** a “2” response to question EA21 (no treatment with Art-AQ, young child dose) **AND** a “2” response to question EA22 (no treatment with Art-AQ, child dose) **AND** a “2” response to question EA23 (no treatment with Art-AQ, older child/adult dose) **AND** a “2” response to question EA24 (no treatment with Artemether-Lumefantrine)], increase the CHW’s numerator by 1 point.
- **Attribute 8.** Client with non-severe febrile illness and cough is evaluated for pneumonia. For each client record with a “1” response to question EA08 (client had cough) **AND** a “1” response to question EA10 (client had fever) **AND** a “2” response to question EA11 (no signs indicating need for referral), increase the CHW’s denominator by 1 point. For each client record with a “1” response to question EA08 (client had cough) **AND** a “1” response to question EA10 (client had fever) **AND** a “2” response to question EA11 (no signs indicating need for referral) **AND** a “1” response to question EA12 (check respiratory rate) **AND** a “1” response to question EA13 (check for chest indrawing), increase the CHW’s numerator by 1 point.
- **Attribute 9.** Client with non-severe febrile illness and non-severe pneumonia is treated with an antibiotic. For each client record with a “1” response to question EA10 (client had fever) **AND** a “2” response to question EA11 (no signs indicating need for referral) **AND** a “1” response to question EA15 (pneumonia classification), increase the CHW’s denominator by 1 point. For each client record with a “1” response to question EA10 (client had fever) **AND** a “2” response to question EA11 (no signs indicating need for referral) **AND** a “1” response to question EA15 (pneumonia classification) **AND** a “1” response to question EA20 (treatment with amoxicillin), increase the CHW’s numerator by 1 point.

*Analysis method for RECO PL CHWs*

Assuming the ideal case in which 5 RECO PL CHWs are identified for each facility and each RECO PL CHW has submitted month reporting forms for all three of the last completed months (and that all these reporting forms can be found) and assuming that data are available to calculate each of the 4 attributes, then for each CHW, there were be a total of 12 values: one value for each of the 4 indicators for each of the 3 months. Each attribute will have a value from 0–100. To calculate that score for a given CHW, use the unweighted average of these 12 values. The CHW score (across all 4 attributes) will have values from 0–100.

- **Attribute 10.** Percentage of recommended household visits done. As the DRC recommendations are for 50 households to be visited each month, for each of the past 3 months for which the number of household visits (question EB03) is available, divide the number of household visits by 50 and multiply by 100. Cap the percentage at 100. For example, if the numbers of households visited for the past 3 months are 45, 50, and 55, then the corresponding Attribute 10 values are 90 (i.e., [45/50] x 100), 100 (i.e., [50/50] x 100), and 100 (i.e., [55/50] x 100, but capped at 100).
- **Attribute 11.** Percentage of households with a child under five years old that slept under an LLIN. For each of the past 3 months for which data are available, the Attribute 11 value equals the EB05 response (households with <5s who slept under an LLIN)/EB04 response (households with an LLIN) x 100. If the calculated value is greater than 100, cap it at 100. Note that this calculation is an estimate of the ideal Attribute 11 value because the monthly reporting form does not include either of the two ideal denominators for this Attribute — i.e., “the number of households with children under 5 years old” and “the number of households with children under 5 years old with an LLIN”. However, it likely that a large majority of households with an LLIN will also have a child under 5 years old — especially, if one considers the types of households that the RECO PL CHWs are likely to visit (i.e., those with children under 5 years old).
- **Attribute 12.** Percentage of women who deliver at home that are referred to a health center. For each of the past 3 months for which data are available, the Attribute 12 value equals the EB07 response (women who deliver at home that are referred)/EB06 response (women who deliver at home) x 100. If the calculated value is greater than 100, cap it at 100.
- **Attribute 13.** Percentage of children under 6 months old who are exclusively breastfed. For each of the past 3 months for which data are available, the Attribute 13 value equals the EB09 response (infants under 6 months exclusively breastfed)/EB08 response (infants under 6 months) x 100. If the calculated value is greater than 100, cap it at 100.

Table 8. Indicators and associated survey questions on CHW quality of care

| **Attribute** | **HHFA indicator** | **t-HFA survey questions** |
| --- | --- | --- |
| Attribute 1. Client with household contact with a TB case patient is referred. RECO site CHW. | - There is no HHFA indicator. Questions developed by the GF t-HFA team. | EA06A to EA06E,  EA07A to EA07E |
| Attribute 2. Client with febrile illness is assessed for important signs and symptoms. RECO site CHW. | - There is no HHFA indicator. Questions developed by the GF t-HFA team. | EA08A to EA08E, EA09A to EA09E,  EA10A to EA10E,  EA11A to EA11E |
| Attribute 3. Client with non-severe febrile illness is tested with a malaria RDT. RECO site CHW. | - There is no HHFA indicator. Questions developed by the GF t-HFA team. | EA10A to EA10E,  EA11A to EA11E,  EA16A to EA16E |
| Attribute 4. Client with severe febrile illness is referred to a health facility. RECO site CHW. | - There is no HHFA indicator. Questions developed by the GF t-HFA team. | EA10A to EA10E,  EA11A to EA11E,  EA25A to EA25E |
| Attribute 5. Client with non-severe febrile illness is correctly classified. RECO site CHW. | - There is no HHFA indicator. Questions developed by the GF t-HFA team. | EA10A to EA10E,  EA11A to EA11E,  EA17A to EA17E,  EA18A to EA18E,  EA19A to EA19E |
| Attribute 6. Client with uncomplicated malaria is treated with ACT. RECO site CHW. | - There is no HHFA indicator. Questions developed by the GF t-HFA team. | EA10A to EA10E,  EA11A to EA11E,  EA17A to EA17E,  EA19A to EA19E,  EA21A to EA21E,  EA22A to EA22E,  EA23A to EA23E,  EA24A to EA24E |
| Attribute 7. Client with non-severe, non-malaria febrile illness is not treated with ACT. RECO site CHW. | - There is no HHFA indicator. Questions developed by the GF t-HFA team. | EA10A to EA10E,  EA11A to EA11E,  EA18A to EA18E,  EA21A to EA21E,  EA22A to EA22E,  EA23A to EA23E,  EA24A to EA24E |
| Attribute 8. Client with non-severe febrile illness and cough is evaluated for pneumonia. RECO site CHW. | - There is no HHFA indicator. Questions developed by the GF t-HFA team. | EA08A to EA08E,  EA10A to EA10E,  EA11A to EA11E,  EA12A to EA12E,  EA13A to EA13E |
| Attribute 9. Client with non-severe febrile illness and non-severe pneumonia is treated with an antibiotic. RECO site CHW. | - There is no HHFA indicator. Questions developed by the GF t-HFA team. | EA10A to EA10E,  EA11A to EA11E,  EA15A to EA15E,  EA20A to EA20E |
| Attribute 10. Percentage of recommended household visits done. RECO PL CHW. | - There is no HHFA indicator. Questions developed by the GF t-HFA team. | EB03A to EB03C |
| Attribute 11. Percentage of households with a child under five years old that slept under an LLIN. RECO PL CHW. | - There is no HHFA indicator. Questions developed by the GF t-HFA team. | EB04A to EB04C,  EB05A to EB05C |
| Attribute 12. Percentage of women who deliver at home that are referred to a health center. RECO PL CHW. | - There is no HHFA indicator. Questions developed by the GF t-HFA team. | EB06A to EB06C,  EB07A to EB07C |
| Attribute 13. Percentage of children under 6 months old who are exclusively breastfed. RECO PL CHW. | - There is no HHFA indicator. Questions developed by the GF t-HFA team. | EB08A to EB08C,  EB09A to EB09C |

**********************************************************************************

1. From IGS: “*Proportion of facilities where pregnant women receive/are offered a HTM disease specific intervention as appropriate for the gestational age of the pregnancy at their visit, as observed during the patient provider interaction. This would include HIV counseling, HIV testing, TB screening and/ or IPTp for malaria - as per the national guidelines.*” From KPI Handbook: “*Broad areas of measurement proposed for refinement via pilot testing are: Integration with and referrals to other HIV prevention and HIV testing services, Counseling and access to ARVs for HIV positive women, Malaria diagnosis and treatment, and TB screening, diagnosis and treatment.*”
