# Supplementary material for: Design and implementation of a multi-country targeted health facility assessment to measure integrated, people-centered, quality of services
Source: PLOS Glob Public Health. 2026 Aug 19;6(8):e0005736. doi: 10.1371/journal.pgph.0005736 (PMC13489417; doi:10.1371/journal.pgph.0005736)
Supplement: Supplementary file 2 [file pgph.0005736.s002.docx]

**S2 Annex. Questionnaire for The Global Fund’s Targeted Health Facility Assessment for use in the Democratic Republic of the Congo (DRC)**

The targeted health facility assessment (t-HFA) questionnaire has eight sections. See Table 1, below.

Table 1. T-HFA questionnaire sections and the Global Fund (GF) indicators measured by questions in the section

| **T-HFA section** | **Topic** | **Global Fund indicators** |
| --- | --- | --- |
| 1 | Facility identifiers and consent | None. Questions in this section are used to map surveyed facilities and to perform stratified analyses of any indicator (e.g., hospitals vs. health centers). |
| 2 | Availability of services, guidelines, equipment, and staff; use of services; and community-led monitoring | HSG-1.1 (availability of guidelines), RSSH/PP RCS-1 (availability of oxygen), RSSH/PP LAB-5 (availability of diagnostic tests), CSS-3 (community-led monitoring), RSSH/PP HRH-1 (health worker vacancy rate), and  RSSH/PP HRH-6 (effective services) |
| 3 | Supervision | Key performance indicator (KPI) S2 (integrated supportive supervision) |
| 4 | Patient exit interviews | KPI S1 (integrated, people-centered services) |
| 5 | Community health worker (CHW) interviews | KPI S5 (systems readiness for CHWs), RSSH/PP HRH-3 (CHW remuneration) |
| 5A | CHW record review | RSSH/PP HRH-4 (CHW quality of care) (for “RECO site” CHWs) |
| 5B | CHW record review | RSSH/PP HRH-4 (CHW quality of care) (for “RECO PL” CHWs) |
| 6 | Record review of ANC clients | KPI S3 (HTM integrated services offered to pregnant women) |
| 7 | Record review of ART clients | KPI S1 (integrated, people-centered services) |
| 8 | Record review of TB clients | RSSH/PP HRH-6 (effective services) |

**QUESTIONNAIRE INTRODUCTION**

Source: The following is adapted from WHO (2023) Harmonized Health Facility Assessment, Module 3: Quality of Care.

The following questionnaire is typically structured into five columns: Column 1: Mod, Column 2: No., Column 3: Question, Column 4: Response, and Column 5: Skip.

- Column 1 - Mod: The letter in Column 1 shows the HHFA module to which the question belongs: A for Availability, R for Readiness, M for Management and finance, or Q for Quality of care. The letters PRE (Patient-Reported Experiences) means that the question came from WHO’s 2023 draft “*Patient-reported experiences in primary care: Patient Questionnaire*.” The letters TBS (TB Screening) mean that the question came from WHO's 2016 ANC guidelines: Recommendation B.1.8 on page 52 of "*WHO recommendations on antenatal care for a positive pregnancy experience.*" The letters tHFA means that the question was developed by the GF t-HFA team; these questions have a letter designation that reflects the section of the questionnaire (e.g., “A” for survey section 1, “B” for survey section 2, etc.) and a question number (e.g., A001, A002, B001, B002, etc.).
- Column 2 – No: Column 2 contains the number of the HHFA question. There may be a single number per question, or a main number with sub-questions below it, e.g., 2401 (main question), 2401_01 (sub-question). Some numbers correspond to an instruction.
- Column 3 - QUESTION: Column 3 contains the question that is read to the respondent by the interviewer. It may also contain additional clarifying information (in non-capitalized font) that the interviewer reads to the respondent. This column may also include instructions (in CAPITALS) to the interviewer. These capitalized instructions are not read to the respondent.
- Column 4 - RESPONSE: Column 4 contains the response options. Different types of response options are used for different types of questions, e.g., pre-coded responses where one or more options are selected, fields requiring entry of a number or text, or combinations of these.
- Column 5 - SKIP: This column contains arrows that instruct the interviewer to skip to a specific question, to the end of a questionnaire section, or to other instructions, if necessary.

The questionnaire also contains text in red font that often include the term “COUNTRY ADAPT”. These sentences highlight questions that may need adaptation to the country context before the survey is finalized for country implementation.

**QUESTIONNAIRE**

| **Mod** | **No.** | **QUESTION** | | | | **RESPONSE** | | | | **SKIP** |  |
| --- | --- | --- | --- | --- | --- | --- | --- | --- | --- | --- | --- |
|  |  | INSTRUCTIONS ON HOW TO PREPARE FOR A VISIT TO A HEALTH FACILITY | | | | | | | |  |  |
|  |  | INSTRUCTIONS FOR THE SURVEY TEAM UPON ARRIVING AT THE HEALTH FACILITY | | | | | | | |  |  |
|  |  | **T-HFA SECTION 1. FACILITY IDENTIFIERS AND CONSENT** | | | | | | | |  |  |
|  |  | [COUNTRY ADAPT NUMBERING FOR REGIONS, DISTRICTS AND FACILITIES BASED ON THE SYSTEM USED IN THE COUNTRY OR THE SYSTEM AGREED UPON FOR THE SURVEY] | | | | | | | |  |  |
| Q | 100 | Facility code | | | | –– –– –– –– –– –– | | | |  |  |
| Q | 102 | Name of facility | | | | ____________________________________ | | | |  |  |
| Q | 100a | Facility sampling category | | | | RANDOM OR PANEL (Auto pulled) | | | |  |  |
| Q | 105 | Name of region/province | | | | NAME OF REGION/PROVINCE:  ______________________________________  REGION/PROVINCE CODE –– –– | | | |  |  |
| Q | 106 | Name of district  [COUNTRY ADAPT NUMBERING FOR REGIONS, DISTRICTS AND FACILITIES BASED ON THE SYSTEM USED IN THE COUNTRY OR THE SYSTEM AGREED UPON FOR THE SURVEY] | | | | NAME OF DISTRICT:  ______________________________________  DISTRICT CODE –– –– | | | |  |  |
| Q |  | 1.1.2. GEOGRAPHIC COORDINATES  RECORD THE GPS READING ACCORDING TO THE INSTRUCTIONS  SET DEFAULT SETTINGS FOR GPS:  1. SET COORDINATE SYSTEM TO LATITUDE/LONGITUDE  2. SET COORDINATE FORMAT TO DECIMAL DEGREES  3. SET DATUM TO WGS84  MOVE TO MAIN ENTRANCE OF THE BUILDING. STAND WITHIN 30 M OF MAIN ENTRANCE WITH VIEW OF SKY: 4. TURN GPS MACHINE ON AND WAIT UNTIL SATELLITE PAGE CHANGES TO "POSITION"  5. WRITE ALTITUDE  6. PRESS "MARK"  7. HIGHLIGHT "AVERAGE" AND PRESS "ENTER"  8. HIGHLIGHT "WAYPOINT NUMBER" AND PRESS "ENTER"  9. ENTER FACILITY CODE  10. WAIT 5 MINUTES  11. HIGHLIGHT "SAVE" AND PRESS "ENTER"  12. PAGE TO MAIN MENU, HIGHLIGHT "WAYPOINT LIST" AND PRESS "ENTER"  13. HIGHLIGHT YOUR WAYPOINT  14. COPY INFORMATION FROM WAYPOINT LIST PAGE ON THE FORM BELOW.  BE SURE TO COPY THE WAYPOINT NAME FROM THE WAYPOINT LIST PAGE TO VERIFY THAT YOU ARE ENTERING THE CORRECT WAYPOINT INFORMATION ON THE DATA FORM. | | | | | | | |  |  |
| Q | 108 | Waypoint name  (facility number) | | | | ___ ___ ___ ___ ___ ___ ___ | | | |  |  |
| Q | 109 | Altitude (m) | | | | ___ ___ ___ ___ | | | |  |  |
| Q | 110 | Latitude | | | | N/S……………………(a) ___  DEGREES………..…(b) ___ ___  DECIMAL…….…….(c) ___ ___ ___ ___ ___ | | | |  |  |
| Q | 111 | Longitude | | | | E/W……………………(a) ___  DEGREES………..…(b) ___ ___  DECIMAL…….…….(c) ___ ___ ___ ___ ___ | | | |  |  |
| tHFA | A004 | DID ANY OF THE FOLLOWING OCCUR WHILE THE SURVEY TEAM WAS ATTEMPTING TO LOCATE THE FACILITY AND BEGIN THE T-HFA VISIT?   - FACILITY VISIT WAS POSTPONED - FACILITY WAS CLOSED - FACILITY WAS DESTROYED - FACILITY WAS NOT FOUND - OTHER SITUATION THAT PREVENTED COMPLETION OF THE SURVEY VISIT | | | | | | | | IF ANY OF THESE 5 SITUATIONS OCCURRED, THEN SKIP TO Q107 AT THE END OF THE QUES-TIONNAIRE |  |
|  |  | IF THE T-HFA VISIT BEGINS BUT THE SURVEY PROCESS IS INTERRUPTED BEFORE IT CAN BE COMPLETED, SKIP TO Q107 AT THE END OF THE QUESTIONNAIRE. ENTER TODAY’S DATE, AND SELECT RESULT CODE 1 FOR “INTERVIEW STARTED BUT NOT COMPLETED”. MAKE A PLAN TO RETURN TO THE FACILITY TO COMPLETE THE T-HFA VISIT. | | | | | | | |  |  |
| Q |  | 1.1.3. CONSENT OF PERSON IN CHARGE OF THE FACILITY ON THE DAY OF THE VISIT | | | | | | | |  |  |
| Q |  | The [survey manager and survey implementer] in close collaboration with the [other relevant entities] are working to collect information about the availability of key health services in different facilities. This information will be collected in selected primary health care and secondary referral facilities across the country. The survey is part of the [government’s] ongoing efforts to understand what services are being offered and where they are being offered. The present study will be conducted across the country. Most facilities included in the survey were selected randomly from a list of all facilities in the country. As the in-charge of this facility, we are asking you to help us to collect the information from the persons who are most knowledgeable about the services. For any questions we ask, if there is another person who is in a better position to provide details, please feel free to refer us to that person. We will want to speak with persons familiar with the various services so that we can correctly identify the components of these services that are offered in this facility. We anticipate that the time required from an individual respondent to complete data collection from a service site may take from 5 to 10 minutes, depending on how busy each separate site is. Your participation in this survey is voluntary and at no cost to you as an individual. You may choose not to participate at all or to stop at any time before the end of the survey. You may also choose not to answer any question that you are not comfortable with. The information on service availability will be shared with the Ministry of Health (MOH) and other relevant stakeholders who support the MOH, to provide information for planning purposes. No names of any respondents will be shared. In case you have any question(s) about this survey at any time, please feel free to contact any of the following people:  [LIST NAMES AND PHONE NUMBERS OF SURVEY MANAGEMENT PERSONS WHO CAN BE CONTACTED]  At this point do you have any questions about the study? Do I have your agreement to proceed? | | | | | | | |  |  |
|  |  | Signature of team leader indicating informed consent was read and agreed by the person in-charge/acting in-charge (sign on the line above) | | |  | Signature of facility staff authorizing data collection and position of the person providing authorization (sign on the line above) | | | |  |  |
| tHFA | A003 | DESIGNATION OF THE PERSON WHO GAVE CONSENT | | | | __________________________________ | | | |  |  |
| Q | 112 | Consent given by facility contact? | | | | YES ................................................................. 1  NO .................................................................. 2 | | | | ➔ END SURVEY |  |
| Q |  | 1.1.4. FACILITY CHARACTERISTICS | | | | | | | |  |  |
| Q | 113 | Type of facility  [COUNTRY ADAPT LIST AND CATEGORIES PRIOR TO IMPLEMENTATION] | | | | NATIONAL REFERRAL HOSPITAL .................... 1  REGIONAL/PROVINCIAL REFERRAL HOSPITAL 2  DISTRICT HOSPITAL ........................................ 3  OTHER GENERAL HOSPITAL ........................... 4  SPECIALTY HOSPITAL ..................................... 5  COMPREHENSIVE HEALTH CENTRE/  POLY CLINIC ................................................... 6  HEALTH CENTRE ............................................ 7  CLINIC/DISPENSARY ...................................... 8  HEALTH POST ................................................ 9  MATERNAL/CHILD HEALTH CLINIC .............. 10  OTHER (Q113A) ___________________ .... 96  (SPECIFY) | | | |  |  |
| Q | 116 | Which of the responses best describes the ownership for this facility?  [COUNTRY ADAPT LIST AND CATEGORIES PRIOR TO IMPLEMENTATION] | | | | *GOVERNMENT/PUBLIC:*  MINISTRY OF HEALTH ................................ 1  LOCAL GOVERNMENT ................................ 2  *GOVERNMENT (INSTITUTIONAL):*  MILITARY/POLICE/NATIONAL GUARD …..... 3  UNIVERSITY ................................................... 4  NGO/NOT-FOR-PROFIT ................................. 5  MISSION/FAITH-BASED ................................. 6  PRIVATE-FOR-PROFIT .................................... 7  OTHER (Q116A) ___________________ .... 96  (SPECIFY) | | | |  |  |
| Q | 117 | RECORD FACILITY LOCATION: URBAN OR RURAL OR PERIURBAN (FROM SURVEY LIST)  NOTE THAT PERIURBAN MEANS AN AREA IMMEDIATELY ADJACENT TO A CITY OR URBAN AREA WITH A MIX OF URBAN AND RURAL CHARACTERISTICS. | | | | URBAN ........................................................... 1  RURAL ............................................................ 2  PERIURBAN .................................................... 3 | | | |  |  |
| Q | 118 | Service levels available | | | | OUTPATIENT ONLY ........................................ 1  INPATIENT ONLY ........................................... 2  BOTH OUT AND INPATIENT ........................... 3 | | | |  |  |
| tHFA | A001 | Which term best describes the level of care provided by this facility? The choices are primary, secondary, and tertiary. Here is what these terms mean. **Primary** is the first place people usually go for health care services. Examples are health posts and health centres. **Secondary** means general hospitals that provide basic inpatient medical and surgical services. Examples are district hospitals. **Tertiary** means referral hospitals that offer highly specialized and complex medical care. Examples are national referral hospitals and academic or teaching hospitals. | | | | PRIMARY…………………………………………………….. 1  SECONDARY……………………………………………….. 2  TERTIARY……………………………………………………. 3  DON’T KNOW ……………………………………………. 8 | | | |  |  |
| tHFA | A002 | What is the population of the catchment area for this facility? THE SURVEYOR SHOULD NOT MAKE ESTIMATIONS—JUST ENTER WHAT THE IN-CHARGE STATES. | | | | ___ ___ ___ ___ ___ ___ ___ ___ ___  FOR DON’T KNOW, ENTER 9 | | | |  |  |
|  |  | **END OF T-HFA SECTION 1** | | | | | | | |  |  |
|  | | | | | | | | | | | |
|  |  | **T-HFA SECTION 2. AVAILABILITY OF SERVICES, GUIDELINES, EQUIPMENT, AND STAFF; USE OF SERVICES; AND**  **COMMUNITY-LED MONITORING**  Sources: WHO (2023) HHFA Module 2: Service readiness, and questions developed by the GF t-HFA Team.  Questions in this section measure GF indicators HSG-1.1, RSSH/PP RCS-1, RSSH/PP LAB-5, CSS-3, RSSH/PP HRH-6, and  RSSH/PP HRH-1. | | | | | | | | |  |
| tHFA | B059 | FIND THE FACILITY STAFF PERSON WHO IS KNOWLEDGEABLE ABOUT THE TYPES OF SERVICES PROVIDED AT THE FACILITY AND THE INTERACTIONS BETWEEN CLIENTS AND THE FACILITY. RECORD THE DESIGNATION OF THE PERSON. | | | | ____________________________________ | | | |  |  |
| tHFA | B035 | How much do you agree with the following statement? You can answer with strongly agree, agree, neither agree nor disagree, disagree, or strongly disagree.  Clients that use this facility provide feedback on the quality of services at this facility. | | | | STRONGLY AGREE ……………………………………….. 1  AGREE …………………………………………………………. 2  NEITHER AGREE NOR DISAGREE ………………….. 3  DISAGREE …………………………………………………….. 4  STRONGLY DISAGREE …………………………………… 5  DON’T KNOW ………………………………………………. 8 | | | |  |  |
| R | 1400 | Does this facility offer diagnosis and/or treatment of malaria? | | | | YES ................................................................... 1  NO ................................................................... 2 | | | | ➔ 1810 |  |
| R | i1401 | ASK TO BE SHOWN THE LOCATION IN THE FACILITY WHERE OUTPATIENT MALARIA SERVICES ARE PROVIDED. FIND THE PERSON MOST KNOWLEDGEABLE ABOUT MALARIA SERVICES IN THE FACILITY. INTRODUCE YOURSELF, EXPLAIN THE PURPOSE OF THE SURVEY AND ASK THE FOLLOWING QUESTIONS. | | | | | | | |  |  |
| tHFA | B060 | RECORD THE DESIGNATION OF THE PERSON THAT YOU FOUND TO ANSWER QUESTIONS ABOUT MALARIA SERVICES. | | | | ____________________________________ | | | |  |  |
| R | 1406 | Are national guidelines for the diagnosis and/or treatment of malaria available in this service site today?  [COUNTRY ADAPT – NAME OF DOCUMENT AND VERSION]  IF YES, ASK: May I see the guidelines? | | | | YES, OBSERVED ............................................. 1  YES, REPORTED, NOT SEEN ........................... 2  NO/NOT AVAILABLE ...................................... 3 | | | |  |  |
| R | 1810 | Does this facility offer antenatal care (ANC) services? | | | | YES ….…………..………………………………………………1  NO ….……………………………………………………………2 | | | | ➔ 2300 |  |
| R | i1811 | ASK TO BE SHOWN THE LOCATION IN THE FACILITY WHERE ANTENATAL CARE SERVICES ARE PROVIDED. FIND THE PERSON MOST KNOWLEDGEABLE ABOUT ANTENATAL CARE SERVICES IN THE FACILITY. INTRODUCE YOURSELF, EXPLAIN THE PURPOSE OF THE SURVEY AND ASK THE FOLLOWING QUESTIONS. | | | | | | | |  |  |
| tHFA | B061 | RECORD THE DESIGNATION OF THE PERSON THAT YOU FOUND TO ANSWER QUESTIONS ABOUT ANTENATAL SERVICES. | | | | ____________________________________ | | | |  |  |
|  |  | I would like to know if the following guidelines are available in this service site today. Please show me the document on: | | | | | | | |  |  |
| R | 1817 _01 | National ANC guidelines  [COUNTRY ADAPT – NAME OF DOCUMENT AND VERSION] | | | | YES, OBSERVED ............................................. 1  YES, REPORTED, NOT SEEN ........................... 2  NO/NOT AVAILABLE ...................................... 3 | | | |  |  |
| R | 2300 | Does this facility offer HIV testing services? | | | | YES ….…………..………………………………………………1  NO ….……………………………………………………………2 | | | | ➔ 2312 |  |
| R | i2304 | ASK TO BE SHOWN THE LOCATION IN THE FACILITY WHERE HIV TESTING SERVICES ARE MOST OFTEN PROVIDED. FIND THE PERSON MOST KNOWLEDGEABLE ABOUT HIV TESTING SERVICES IN THE FACILITY. INTRODUCE YOURSELF, EXPLAIN THE PURPOSE OF THE SURVEY AND ASK THE FOLLOWING QUESTIONS. | | | | | | | |  |  |
| tHFA | B062 | RECORD THE DESIGNATION OF THE PERSON THAT YOU FOUND TO ANSWER QUESTIONS ABOUT HIV TESTING SERVICES. | | | | ____________________________________ | | | |  |  |
| R | i2307 | I would like to know if the following guidelines are available in this service site today. Please show me the document on: | | | | | | | |  |  |
| R | 2308 _01 | National guidelines for HIV counselling and testing  [COUNTRY ADAPT – NAME OF DOCUMENT AND VERSION] | | | | YES, OBSERVED .............................................. 1  YES, REPORTED, NOT SEEN ............................ 2  NO/NOT AVAILABLE ...................................... 3 | | | |  |  |
| tHFA | B039 | Does the facility implement the WHO recommended 3 test HIV algorithm?  [COUNTRY ADAPT - ADD AN IMAGE OF THE 3-TEST HIV ALGORITHM] | | | | YES …………………………………………………………….. 1    NO, AND THERE ARE **NO**  REFORMS UNDERWAY TO  ADOPT A NEW ALGORITHM ……………………….. 2    NO, BUT THERE **ARE**  REFORMS UNDERWAY TO  ADOPT A NEW ALGORITHM ……………………….. 3    UNKNOWN ………………………………………………… 8 | | | |  |  |
| tHFA | B042 | Does this facility offer HIV CD4 testing services? | | | | YES ….…………..………………………………………………1  NO ….……………………………………………………………2  DON’T KNOW …………………………………………….. 8 | | | |  |  |
| tHFA | B043 | Does this facility offer HIV viral load monitoring services? | | | | YES ….…………..………………………………………………1  NO ….……………………………………………………………2  DON’T KNOW …………………………………………….. 8 | | | |  |  |
| tHFA | B044 | Does this facility offer HIV Early Infant Diagnostic (EID) testing services? | | | | YES ….…………..………………………………………………1  NO ….……………………………………………………………2  DON’T KNOW …………………………………………….. 8 | | | |  |  |
| R | 2312 | Does this facility provide or prescribe ART or provide follow-up services for any ART patients? | | | | YES ….…………..………………………………………………1  NO ….……………………………………………………………2 | | | | ➔ 2400 |  |
| R | i2313 | ASK TO BE SHOWN THE LOCATION IN THE FACILITY WHERE HIV TREATMENT, CARE AND SUPPORT SERVICES ARE PROVIDED. FIND THE PERSON MOST KNOWLEDGEABLE ABOUT HIV TREATMENT, CARE AND SUPPORT SERVICES IN THE FACILITY. INTRODUCE YOURSELF, EXPLAIN THE PURPOSE OF THE SURVEY AND ASK THE FOLLOWING QUESTIONS. | | | | | | | |  |  |
| tHFA | B063 | RECORD THE DESIGNATION OF THE PERSON THAT YOU FOUND TO ANSWER QUESTIONS ABOUT HIV TREATMENT, CARE, AND SUPPORT SERVICES. | | | | ____________________________________ | | | |  |  |
| R | i2315A | I would like to know if the following guidelines are available in this service site today. Please show me the document on: | | | | | | | |  |  |
| R | 2315B _01 | National ART guidelines  [COUNTRY ADAPT – NAME OF DOCUMENT AND VERSION] | | | | YES, OBSERVED ............................................. 1  YES, REPORTED, NOT SEEN ........................... 2  NO/NOT AVAILABLE ...................................... 3 | | | |  |  |
| R | 2400 | Does this facility provide any services for tuberculosis? This includes case detection, diagnosis, prescribing treatment, patient clinical follow-up, patient follow-up for treatment adherence, and/or periodic resupply of individual patient medicines. | | | | YES ….…………..………………………………………………1  NO ….……………………………………………………………2 | | | | ➔ 1323 |  |
| R | i2401 | First, I want to know about any TB testing and diagnosis. I would like to first speak with the most knowledgeable person in the facility about routine practices related to TB testing and diagnosis.  INTRODUCE YOURSELF, EXPLAIN THE PURPOSE OF THE SURVEY AND ASK THE FOLLOWING QUESTIONS. | | | | | | | |  |  |
| tHFA | B064 | RECORD THE DESIGNATION OF THE PERSON THAT YOU FOUND TO ANSWER QUESTIONS ABOUT TB TESTING AND DIAGNOSIS. | | | | ____________________________________ | | | |  |  |
| R | i2418 | I would like to know if the following guidelines are available in this service site today. | | | | | | | |  |  |
| R | 2419 | For each guideline that I ask about, please show it to me.  THESE MAY BE IN ONE GUIDELINE OR IN DIFFERENT DOCUMENTS. | | | |  | | | |  |  |
| R | 01 | National guidelines for diagnosis and management of TB in adults  [COUNTRY ADAPT – NAME OF DOCUMENT AND VERSION] | | | | YES, OBSERVED ............................................. 1  YES, REPORTED, NOT SEEN ........................... 2  NO/NOT AVAILABLE ...................................... 3 | | | |  |  |
| R | 03 | National guidelines for diagnosis and management of TB in children  [COUNTRY ADAPT – NAME OF DOCUMENT AND VERSION] | | | | YES, OBSERVED ............................................. 1  YES, REPORTED, NOT SEEN ........................... 2  NO/NOT AVAILABLE ...................................... 3 | | | |  |  |
|  |  | ASK TO BE SHOWN THE LOCATION IN THE FACILITY WHERE OUTPATIENT SERVICES ARE PROVIDED. FIND THE PERSON MOST KNOWLEDGEABLE ABOUT OXYGEN DELIVERY. INTRODUCE YOURSELF, EXPLAIN THE PURPOSE OF THE SURVEY AND ASK THE FOLLOWING QUESTIONS. | | | | | | | |  |  |
| tHFA | B065 | RECORD THE DESIGNATION OF THE PERSON THAT YOU FOUND TO ANSWER QUESTIONS ABOUT OXYGEN DELIVERY. | | | | ____________________________________ | | | |  |  |
| R | 1323 | Now I would like to know about the availability of oxygen for patients in the general outpatient service area or unit. Does this unit ever provide oxygen to patients? | | | | YES ….…………..………………………………………………1  NO ….……………………………………………………………2 | | | | ➔ 2900 |  |
| R | 1324 | Is there any oxygen currently available in this unit? | | | | YES ….…………..………………………………………………1  NO ….……………………………………………………………2 | | | |  |  |
| R | 1325 | For each item that I ask about, please show me the item and, when relevant, tell me if it is functioning or not. | (A) AVAILABLE | | | | (B) FUNCTIONAL | | |  |  |
|  |  |  | OBSERVED | REPORTED, NOT SEEN | | NO | YES | NO | DON’T KNOW |  |  |
| R | 01 | Centrally piped oxygen supply | 1 ➔ B | 2 ➔ B | | 3 ➔ 02 | 1 | 2 | 8 |  |  |
| R | 02 | Oxygen concentrator | 1 ➔ B | 2 ➔ B | | 3 ➔ 03 | 1 | 2 | 8 |  |  |
| R | 03 | Oxygen tank or cylinder with attached pressure gauge, pressure regulator | 1 ➔ B | 2 ➔ B | | 3 ➔ 04 | 1 | 2 | 8 |  |  |
| R | 04 | Flowmeter for oxygen source, with gradations in mL | 1 ➔ B | 2 ➔ B | | 3 ➔ 05 | 1 | 2 | 8 |  |  |
| R | 05 | Humidifier | 1 ➔ B | 2 ➔ B | | 3 ➔ 06 | 1 | 2 | 8 |  |  |
| R | 06 | Oxygen delivery apparatus (key connecting tubes and mask or nasal prongs) | 1 ➔ B | 2 ➔ B | | 3 ➔ 1322_09 | 1 | 2 | 8 |  |  |
| R | 1322  _09 | Pulse oximeter | 1 ➔ B | 2 ➔ B | | 3 ➔ 1326 | 1 | 2 | 8 |  |  |
| R | 1326 | Can oxygen be brought to this unit from a different unit or facility location if needed? | | | | YES ….…………..………………………………………………1  NO ….……………………………………………………………2 | | | |  |  |
| tHFA | 2900 | Does this facility conduct any diagnostic testing of specimens using either laboratory equipment or rapid diagnostic tests with a dedicated laboratory in the facility? This includes tests performed in a laboratory or in a service site at this facility, as well as sending a specimen outside for testing and receiving the results back. | | | | YES, with laboratory in the facility………………………………………………………………1  YES, without laboratory in the facility…………..2  NO LABORATORY TESTS PERFORMED………..…3 | | | | ➔ B036 |  |
| R_C | i2901 | ASK TO BE SHOWN THE MAIN LABORATORY IN THE FACILITY OR THE LOCATION IN THE FACILITY WHERE MOST TESTING IS DONE. FIND THE PERSON MOST KNOWLEDGEABLE ABOUT LABORATORY TESTS CONDUCTED BY THIS FACILITY. INTRODUCE YOURSELF, EXPLAIN THE PURPOSE OF THE SURVEY AND ASK THE FOLLOWING QUESTIONS.  I am interested in learning about any diagnostic tests conducted by this facility or about tests where the facility collects specimens that are sent elsewhere for testing where the results are returned to this facility for use. The questions I ask may apply to a special laboratory service area, or sometimes may refer to service sites where diagnostic tests are conducted or where specimens are collected and sent outside the facility for testing. | | | | | | | |  |  |
| tHFA | B066 | RECORD THE DESIGNATION OF THE PERSON THAT YOU FOUND TO ANSWER QUESTIONS ABOUT LABORATORY TESTS. | | | | ____________________________________ | | | |  |  |
| tHFA | B001 | Does this health facility comply with the national guidelines for essential diagnostics list or test menu? Please show me the document. | | | | YES, COMPLIES AND DOCUMENT  OBSERVED ............................................. 1  YES, COMPLIES BUT DOCUMENT  NOT SEEN ........................... 2  NO, DOES NOT COMPLY ................................ 3 | | | |  |  |
|  |  |  | | | | | | | |  |  |
|  |  | TEST NAME | | | | ASK THE LABORATORY STAFF PERSON IF THE TEST IS CONDUCTED (Includes tests for which specimens are sent outside and results are received back) | | | |  |  |
| tHFA | TEST1 | Antibodies to HIV 1/2 (anti‑HIV Ab) | | | | YES ................................................................. 1  NO .................................................................. 2  DON’T KNOW …………………………………………….. 8 | | | |  |  |
| tHFA | TEST2 | Qualitative (EID) or quantitative (viral load) HIV nucleic acid test (NAT) | | | | YES ................................................................. 1  NO .................................................................. 2  DON’T KNOW …………………………………………….. 8 | | | |  |  |
| tHFA | TEST3 | CD4 cell enumeration | | | | YES ................................................................. 1  NO .................................................................. 2  DON’T KNOW …………………………………………….. 8 | | | |  |  |
| tHFA | TEST4 | Influenza A and B antigen | | | | YES ................................................................. 1  NO .................................................................. 2  DON’T KNOW …………………………………………….. 8 | | | |  |  |
| tHFA | TEST5 | Malaria antigen RDT or Malaria parasite light microscopy (Plasmodium spp. antigens; species specific (e.g. HRP2) and/or pan species-specific (e.g. pan-pLDH)) | | | | YES ................................................................. 1  NO .................................................................. 2  DON’T KNOW …………………………………………….. 8 | | | |  |  |
| tHFA | TEST6 | Antibodies to Treponema pallidum OR Combined antibodies to T. pallidum and HIV 1/2 | | | | YES ................................................................. 1  NO .................................................................. 2  DON’T KNOW …………………………………………….. 8 | | | |  |  |
| tHFA | TEST7 | Lipoarabinomannan (LAM) antigen | | | | YES ................................................................. 1  NO .................................................................. 2  DON’T KNOW …………………………………………….. 8 | | | |  |  |
| tHFA | TEST8 | Staining procedures | | | | YES ................................................................. 1  NO .................................................................. 2  DON’T KNOW …………………………………………….. 8 | | | |  |  |
| tHFA | TEST9 | Culture OR Culture and Genus and species identification of bacteria and fungi | | | | YES ................................................................. 1  NO .................................................................. 2  DON’T KNOW …………………………………………….. 8 | | | |  |  |
| tHFA | TEST10 | Blood culture | | | | YES ................................................................. 1  NO .................................................................. 2  DON’T KNOW …………………………………………….. 8 | | | |  |  |
| tHFA | TEST11 | Antimicrobial susceptibility testing (AST) | | | | YES ................................................................. 1  NO .................................................................. 2  DON’T KNOW …………………………………………….. 8 | | | |  |  |
| tHFA | TEST12 | Liver profile/Liver Function test  (Alanine aminotransferase (ALT) + Aspartate aminotransferase (AST)+Albumin + Alkaline phosphatase (ALP) +Gamma-glutamyl transferase (GGT) +Globulin+ Total protein+Total bilirubin+Direct bilirubin and indirect bilirubin) | | | | YES ................................................................. 1  NO .................................................................. 2  DON’T KNOW …………………………………………….. 8 | | | |  |  |
| tHFA | TEST13 | Renal function test  Albumin + blood urea nitrogen (BUN) + total calcium + ionized calcium + creatinine + electrolytes (sodium, potassium, chloride, bicarbonate, magnesium, and phosphate) | | | | Check YES if albumin is present and ignore all the other components (e.g., BUN, total calcium, etc.) of the question.  YES ................................................................. 1  NO .................................................................. 2  DON’T KNOW …………………………………………….. 8 | | | |  |  |
| tHFA | TEST14 | Blood gas | | | | YES ................................................................. 1  NO .................................................................. 2  DON’T KNOW …………………………………………….. 8 | | | |  |  |
| tHFA | TEST15 | C-reactive protein (CRP) | | | | YES ................................................................. 1  NO .................................................................. 2  DON’T KNOW …………………………………………….. 8 | | | |  |  |
| tHFA | TEST16 | Glucose  This can be either a blood or urine test for glucose. | | | | YES ................................................................. 1  NO .................................................................. 2  DON’T KNOW …………………………………………….. 8 | | | |  |  |
| tHFA | TEST17 | Complete blood count (CBC), automated | | | | YES ................................................................. 1  NO .................................................................. 2  DON’T KNOW …………………………………………….. 8 | | | |  |  |
| tHFA | TEST18 | Prothrombin time and international normalized ratio (PT/INR) | | | | YES ................................................................. 1  NO .................................................................. 2  DON’T KNOW …………………………………………….. 8 | | | |  |  |
| tHFA | TEST19 | SARS-CoV-2 test | | | | YES ................................................................. 1  NO .................................................................. 2  DON’T KNOW …………………………………………….. 8 | | | |  |  |
| tHFA | TEST20 | Hepatitis B surface antigen (HBsAg) | | | | YES ................................................................. 1  NO .................................................................. 2  DON’T KNOW …………………………………………….. 8 | | | |  |  |
| tHFA | TEST21 | Influenza A and B diagnostic | | | | YES ................................................................. 1  NO .................................................................. 2  DON’T KNOW …………………………………………….. 8 | | | |  |  |
| tHFA | TEST22 | Qualitative dengue virus nucleic acid test, Dengue virus IgM antibody or Dengue virus antigen (NS1) | | | | YES ................................................................. 1  NO .................................................................. 2  DON’T KNOW …………………………………………….. 8 | | | |  |  |
| tHFA | TEST23 | Qualitative nucleic acid test (NAT) for Chlamydia trachomatis (CT) and Neisseria gonorrhoeae (NG) infections | | | | YES ................................................................. 1  NO .................................................................. 2  DON’T KNOW …………………………………………….. 8 | | | |  |  |
| tHFA | TEST24 | Mycobacterium tuberculosis bacteria | | | | YES ................................................................. 1  NO .................................................................. 2  DON’T KNOW …………………………………………….. 8 | | | |  |  |
| tHFA | TEST25 | M. tuberculosis DNA | | | | YES ................................................................. 1  NO .................................................................. 2  DON’T KNOW …………………………………………….. 8 | | | |  |  |
| tHFA | TEST26 | Drug susceptibility testing of M. tuberculosis culture | | | | YES ................................................................. 1  NO .................................................................. 2  DON’T KNOW …………………………………………….. 8 | | | |  |  |
| tHFA | TEST27 | Measles nucleic acid test or Measle serology | | | | YES ................................................................. 1  NO .................................................................. 2  DON’T KNOW …………………………………………….. 8 | | | |  |  |
| tHFA | TEST28 | Vibrio cholerae antigen | | | | YES ................................................................. 1  NO .................................................................. 2  DON’T KNOW …………………………………………….. 8 | | | |  |  |
| tHFA | B011 | Does the laboratory use an integrated sample transport system for referral of samples? | | | | YES………………………………………………………………1  NO, IT IS A DISEASE-SPECIFIC SAMPLE  TRANSPORT SYSTEM…….…………………………….2  NO, IT IS AN AD-HOC TRANSPORT SYSTEM… 3  NO, A TRANSPORT SYSTEM IS UNNECESSARY  BECAUSE THE FACILITY DOES NOT SEND  SPECIMENS OUT FOR TESTING ……………….. 4  DON’T KNOW ……………………………….…………… 8 | | | |  |  |
| tHFA | B012 | Does the facility have an electronic Laboratory Information System (LIS)? A Laboratory Information System implies a computer-based registry and software program that records and manages information on patient samples, type and number of tests performed, tracks orders and results related to lab tests, and/or has other functionalities relating to lab data management. | | | | YES ….…………..………………………………………………1  NO ….……………………………………………………………2  DON’T KNOW ………………………………………………8 | | | |  |  |
| tHFA | B013 | Does the laboratory have an electronic test ordering and results return capability? | | | | YES ….…………..………………………………………………1  NO ….……………………………………………………………2  DON’T KNOW ………………………………………………8 | | | |  |  |
| tHFA | B014 | Does the laboratory participate in an External Quality Assurance (EQA) program for HIV serology testing? | | | | YES, INTERNATIONAL PROVIDER …………………1  YES, NATIONAL PROVIDER …………………………. 2  YES, INTERNATIONAL **AND**  NATIONAL PROVIDER ……………………………... 3  NO …………………………………………………………….. 4  DON’T KNOW ………………………………………………8 | | | |  |  |
| tHFA | B040 | Does the laboratory participate in an External Quality Assurance (EQA) program for HIV viral load monitoring? | | | | YES, INTERNATIONAL PROVIDER …………………1  YES, NATIONAL PROVIDER …………………………. 2  YES, INTERNATIONAL **AND**  NATIONAL PROVIDER ……………………………... 3  NO …………………………………………………………….. 4  DON’T KNOW ………………………………………………8 | | | |  |  |
| tHFA | B015 | Does the laboratory participate in an External Quality Assurance (EQA) program for TB sputum microscopy? | | | | YES, INTERNATIONAL PROVIDER …………………1  YES, NATIONAL PROVIDER …………………………. 2  YES, INTERNATIONAL **AND**  NATIONAL PROVIDER ……………………………... 3  NO …………………………………………………………….. 4  DON’T KNOW ………………………………………………8 | | | |  |  |
| tHFA | B041 | Does the laboratory participate in an External Quality Assurance (EQA) program for TB molecular tests? | | | | YES, INTERNATIONAL PROVIDER …………………1  YES, NATIONAL PROVIDER …………………………. 2  YES, INTERNATIONAL **AND**  NATIONAL PROVIDER ……………………………... 3  NO …………………………………………………………….. 4  DON’T KNOW ………………………………………………8 | | | |  |  |
| tHFA | B016 | Does the laboratory participate in an External Quality Assurance (EQA) program for malaria testing, including microscopy? | | | | YES, INTERNATIONAL PROVIDER …………………1  YES, NATIONAL PROVIDER …………………………. 2  YES, INTERNATIONAL **AND**  NATIONAL PROVIDER ……………………………... 3  NO …………………………………………………………….. 4  DON’T KNOW ………………………………………………8 | | | |  |  |
| tHFA | B017 | Does the laboratory participate in an External Quality Assurance (EQA) program for any other test? | | | | YES, INTERNATIONAL PROVIDER …………………1  YES, NATIONAL PROVIDER …………………………. 2  YES, INTERNATIONAL **AND**  NATIONAL PROVIDER ……………………………... 3  NO …………………………………………………………….. 4  DON’T KNOW ………………………………………………8 | | | |  |  |
| tHFA | B018 | Have you or any laboratory staff received biosafety training in the past 2 years? | | | | YES, ALL STAFF................................................ 1 YES, SOME BUT NOT ALL STAFF………………….. 2 NO.................................................................. 3  DON’T KNOW ………………………………………………8 | | | |  |  |
| tHFA | B019 | Is molecular equipment at this facility used to test for one or more diseases? | | | | YES…………………………………………………………….. 1  NO MOLECULAR TEST AVAILABLE…….………… 2  DON’T KNOW …………………………………….……… 8 | | | | ➔ B036  ➔ B036 |  |
| tHFA | B020 | If yes, which diseases can be tested using molecular equipment at this facility today? In other words, for each of the following diseases, is the equipment functional, and are there reagents and staff to perform the test at the facility today? | | | | (A) HIV - Early infant diagnosis (EID)  YES ….…………..………………………………………………1  NO ….……………………………………………………………2  DON’T KNOW ………………………………………………8  (B) HIV - Viral load (VL) monitoring  YES ….…………..………………………………………………1  NO ….……………………………………………………………2  DON’T KNOW ………………………………………………8  (C) TB  YES ….…………..………………………………………………1  NO ….……………………………………………………………2  DON’T KNOW ………………………………………………8  (D) COVID-19  YES ….…………..………………………………………………1  NO ….……………………………………………………………2  DON’T KNOW ………………………………………………8  (E) Human Papilloma Virus (HPV)  YES ….…………..………………………………………………1  NO ….……………………………………………………………2  DON’T KNOW ………………………………………………8  (F) Hepatitis C Virus (HCV)  YES ….…………..………………………………………………1  NO ….……………………………………………………………2  DON’T KNOW ………………………………………………8  (G) Hepatitis B Virus (HBV)  YES ….…………..………………………………………………1  NO ….……………………………………………………………2  DON’T KNOW ………………………………………………8  (H) Neisseria gonorrhea/Chlamydia trachomatis  YES ….…………..………………………………………………1  NO ….……………………………………………………………2  DON’T KNOW ………………………………………………8  (J1) Other (specify): _____________________  (J2) Response for the specified test (above)  YES ….…………..………………………………………………1  NO ….……………………………………………………………2  DON’T KNOW ………………………………………………8 | | | |  |  |
| tHFA | B036 | Which method is primarily used to dispose of infectious and sharps healthcare waste? DO NOT READ CHOICES. SELECT ONLY ONE CHOICE. INTERVIEWER MIGHT NEED TO PROBE (FOR EXAMPLE, IF THE RESPONDENT STATES THAT THE FACILITY BURNS WASTE IN A PIT, THE INTERVIEWER MUST ASK IF THE BURN PIT IS FENCED OR SECURED). | | | | PROCESS USING A NON-BURNING TREATMENT TECHNOLOGY (E.G AUTOCLAVE, MICROWAVE, CHEMICAL, FRICTIONAL) ……… 1  BURN IN INCINERATOR DESIGNED FOR POLLUTION ABATEMENT. POLLUTION ABATEMENT MEANS REDUCED POLLUTION, FOR EXAMPLE AN INCINERATOR THAT  BURNS AT A HIGH TEMPERATURE. …………….. 2  BURN IN AN INCINERATOR WITHOUT POLLUTION ABATEMENT …………………………… 3  OPEN BURNING IN A FENCED/SECURED WASTE PIT …………………………………………………. 4  OPEN BURNING IN A  NON-SECURED WASTE PIT …………………………. 5  DUMP WITHOUT BURNING ………………………… 6  REMOVE OFFSITE TO LICENSED  TREATMENT FACILITY ………………………………… 7  REMOVE OFFSITE TO UNLICENSED  LOCATION OR DUMP …………………………………. 8  REMOVE OFFSITE BUT DO NOT KNOW DETAILS OF HOW WASTE IS TREATED  OR DISPOSED …………………………………………….. 9  OTHER ……………………………………………………… 10 | | | | ➔ B021 (CLM)      ➔ B021 (CLM)    ➔ B021 (CLM) |  |
| tHFA | B037 | Is the primary method of waste disposal functional?  SKIP THIS QUESTION IF WASTE IS REMOVED OFFSITE | | | | ALWAYS …………………………………………………….. 1  MOST OF THE TIME …………………………………… 2  SOMETIMES ………………………………………………. 3  NEVER ……………………………………………………….. 4  DON’T KNOW ………………………………………………8 | | | |  |  |
| tHFA | B038 | Is the primary method of waste disposal of a sufficient capacity for the waste that is generated at this facility?  SKIP THIS QUESTION IF WASTE IS REMOVED OFFSITE | | | | YES ….…………..………………………………………………1  NO ….……………………………………………………………2  DON’T KNOW ………………………………………………8 | | | |  |  |
|  |  | FIND THE STAFF PERSON AT THE FACILITY THAT IS MOST KNOWLEDGEABLE ABOUT COMMUNITY-LED MONITORING OR COMMUNITY-LEVEL HEALTH ACTIVITIES. INTRODUCE YOURSELF, EXPLAIN THE PURPOSE OF THE SURVEY AND ASK THE FOLLOWING QUESTIONS.  I would like to know if the community that this facility serves has a community-led monitoring mechanism in place. The term “community-led monitoring” means an independent accountability mechanism that is designed, led, and implemented by local community organizations that work closely with clients and vulnerable populations. Through community-led monitoring, clients and other community members use structured data collection and analysis to produce recommendations for improved accessibility, acceptability, affordability, quality, and impact of health programs and services.  With that background in mind, I would now like to ask some specific questions. | | | | | | | |  |  |
| tHFA | B067 | RECORD THE DESIGNATION OF THE PERSON THAT YOU FOUND TO ANSWER QUESTIONS ABOUT COMMUNITY-LED MONITORING OR COMMUNITY-LEVEL HEALTH ACTIVITIES. | | | | ____________________________________ | | | |  |  |
|  |  | How much do you agree with the following statements? You can answer with strongly agree, agree, neither agree nor disagree, disagree, or strongly disagree. | | | |  | | | |  |  |
| tHFA | B021 | The community that this facility serves has trained community data collectors that routinely collect information on the availability, accessibility, acceptability, and quality of HIV, TB, or malaria services. | | | | STRONGLY AGREE ………………………….………….. 1  AGREE ……………………………………………….………. 2  NEITHER AGREE NOR DISAGREE ……….……….. 3  DISAGREE ………………………………………………….. 4  STRONGLY DISAGREE …………………….…………… 5 DON’T KNOW ………………………………………………8 | | | | ➔ B068  ➔ B068  ➔ B068  ➔ B068 |  |
|  |  | IF STRONGLY AGREE OR AGREE, ASK: | | | |  | | | |  |  |
| tHFA | B022 | The community provides feedback to this facility on (at least) a quarterly basis. | | | | STRONGLY AGREE ………………………….………….. 1  AGREE ……………………………………………….………. 2  NEITHER AGREE NOR DISAGREE ……….……….. 3  DISAGREE ………………………………………………….. 4  STRONGLY DISAGREE …………………….…………… 5 DON’T KNOW ………………………………………………8 | | | |  |  |
| tHFA | B045 | This facility routinely analyzes and uses the community-led monitoring data for quality improvement. | | | | STRONGLY AGREE ………………………….………….. 1  AGREE ……………………………………………….………. 2  NEITHER AGREE NOR DISAGREE ……….……….. 3  DISAGREE ………………………………………………….. 4  STRONGLY DISAGREE …………………….…………… 5 DON’T KNOW ………………………………………………8 | | | |  |  |
| tHFA | B023 | Health care staff at this facility are aware of the community-led monitoring mechanism. | | | | STRONGLY AGREE ………………………….………….. 1  AGREE ……………………………………………….………. 2  NEITHER AGREE NOR DISAGREE ……….……….. 3  DISAGREE ………………………………………………….. 4  STRONGLY DISAGREE …………………….…………… 5 DON’T KNOW ………………………………………………8 | | | | ➔ B068  ➔ B068  ➔ B068  ➔ B068 |  |
|  |  | IF STRONGLY AGREE OR AGREE, ASK: | | | |  | | | |  |  |
| tHFA | B024 | Health care staff inform the clients at this facility of the existence of the community-led monitoring mechanism and promote its use. | | | | STRONGLY AGREE ………………………….………….. 1  AGREE ……………………………………………….………. 2  NEITHER AGREE NOR DISAGREE ……….……….. 3  DISAGREE ………………………………………………….. 4  STRONGLY DISAGREE …………………….…………… 5 DON’T KNOW ………………………………………………8 | | | |  |  |
| tHFA | B068 | FIND THE PERSON THAT IS MOST KNOWLEDGEABLE ABOUT STAFFING AND STAFF WORK SCHEDULES. INTRODUCE YOURSELF, EXPLAIN THE PURPOSE OF THE SURVEY AND ASK THE FOLLOWING QUESTIONS. | | | | | | | |  |  |
|  |  | RECORD THE DESIGNATION OF THE PERSON THAT YOU FOUND TO ANSWER QUESTIONS ABOUT STAFFING AND STAFF WORK SCHEDULES. | | | | ____________________________________ | | | |  |  |
| tHFA | B025 | How many staff that provide clinical services should be present at the facility today at this time? In other words, how many clinical providers are scheduled to be working at the facility today at this time? | | | | NUMBER OF PROVIDERS ____ ____ ____  ENTER “999” FOR DON’T KNOW | | | |  |  |
| tHFA | B026 | Of the clinical providers that should be present at the facility today at this time (from the previous question), how many are actually present at the facility at this time? | | | | NUMBER OF PROVIDERS ____ ____ ____  ENTER “999” FOR DON’T KNOW | | | |  |  |
| tHFA | B046 | What is the total number of funded full-time posts at this facility? That includes filled and unfilled posts. | | | | NUMBER OF FULL-TIME POSTS ____ ____ ____  ENTER “999” FOR DON’T KNOW | | | |  |  |
| tHFA | B047 | How many funded full-time posts do you have at this facility that have not been filled for at least six months? | | | | NUMBER OF FULL-TIME POSTS ____ ____ ____  ENTER “999” FOR DON’T KNOW | | | |  |  |
|  |  | I would now like to ask you these same two questions about specific health worker types. | | | | | | | |  |  |
| tHFA | B048 | What is the number of funded full-time posts at this facility for **physicians**? That includes filled and unfilled posts. | | | | NUMBER OF FULL-TIME POSTS ____ ____ ____  ENTER “999” FOR DON’T KNOW | | | |  |  |
| tHFA | B049 | How many funded full-time posts do you have at this facility for **physicians** that have not been filled for at least six months? | | | | NUMBER OF FULL-TIME POSTS ____ ____ ____  ENTER “999” FOR DON’T KNOW | | | |  |  |
| tHFA | B050 | What is the number of funded full-time posts at this facility for **nurses and midwives**? That includes filled and unfilled posts. | | | | NUMBER OF FULL-TIME POSTS ____ ____ ____  ENTER “999” FOR DON’T KNOW | | | |  |  |
| tHFA | B051 | How many funded full-time posts do you have at this facility for **nurses and midwives** that have not been filled for at least six months? | | | | NUMBER OF FULL-TIME POSTS ____ ____ ____  ENTER “999” FOR DON’T KNOW | | | |  |  |
| tHFA | B052 | What is the number of funded full-time posts at this facility for **lab technicians**? That includes filled and unfilled posts. | | | | NUMBER OF FULL-TIME POSTS ____ ____ ____  ENTER “999” FOR DON’T KNOW | | | |  |  |
| tHFA | B053 | How many funded full-time posts do you have at this facility for **lab technicians** that have not been filled for at least six months? | | | | NUMBER OF FULL-TIME POSTS ____ ____ ____  ENTER “999” FOR DON’T KNOW | | | |  |  |
| tHFA | B054 | What is the number of funded full-time posts at this facility for **pharmacists**? That includes filled and unfilled posts. | | | | NUMBER OF FULL-TIME POSTS ____ ____ ____  ENTER “999” FOR DON’T KNOW | | | |  |  |
| tHFA | B055 | How many funded full-time posts do you have at this facility for **pharmacists** that have not been filled for at least six months? | | | | NUMBER OF FULL-TIME POSTS ____ ____ ____  ENTER “999” FOR DON’T KNOW | | | |  |  |
| tHFA | B056 | What is the number of funded full-time posts at this facility for **community health workers**? That includes filled and unfilled posts. | | | | NUMBER OF FULL-TIME POSTS ____ ____ ____  ENTER “999” FOR DON’T KNOW | | | |  |  |
| tHFA | B057 | How many funded full-time posts do you have at this facility for **community health workers** that have not been filled for at least six months? | | | | NUMBER OF FULL-TIME POSTS ____ ____ ____  ENTER “999” FOR DON’T KNOW | | | |  |  |
|  |  | I have some additional questions about staff at the facility and their non-clinical functions. If there is another person who can answer these questions, please let me know. | | | | | | | |  |  |
| tHFA | B069 | RECORD THE DESIGNATION OF THE PERSON THAT YOU FOUND TO ANSWER QUESTIONS ABOUT STAFF AT THE FACILITY AND THEIR NON-CLINICAL FUNCTIONS. | | | | ____________________________________ | | | |  |  |
| tHFA | B027 | What percentage of your staff have been fully vaccinated against COVID-19 per the country guidelines? | | | | 80% OR MORE ............………………..……………. 1  60 – 79% …………………………………………………… 2  40 – 59% …………………………………………………… 3  20 – 39% ………………………………………………….. 4  LESS THAN 20% ………………………………………… 5  DON’T KNOW …………………………………………… 8 | | | |  |  |
| tHFA | B029 | At the facility level, is there a routine process for reviewing data on facility services, outcomes, or patient feedback? | | | | YES ................................................................. 1  NO .................................................................. 2  DON’T KNOW …………………………………………….. 8 | | | |  |  |
| tHFA | B030 | Does this facility routinely carry out quality assurance activities for any service areas? By this I mean some formal review system or comparison of work or systems to a standard. | | | | YES ................................................................. 1  NO .................................................................. 2  DON’T KNOW …………………………………………….. 8 | | | |  |  |
| tHFA | B031 | Which of the following choices best describes how many clinical staff and cleaners at this facility have received training on infection prevention and control guidelines and standard operating procedures since they began working at this facility?  READ THE FIRST FOUR RESPONSES. | | | | ALL STAFF …………………………………………………… 1  MAJORITY OF STAFF …………………………………... 2  SOME STAFF …………………………………………..….. 3  NO STAFF ……………………….………………………..… 4  DON’T KNOW ………….…………………………………. 8 | | | |  |  |
| R | 2000 | Does this facility offer any preventive and/or curative care services for children under 5? | | | | YES ….…………..………………………………………………1  NO ….……………………………………………………………2 | | | | ➔ 2100 |  |
| R | i2001 | ASK TO BE SHOWN THE LOCATION IN THE FACILITY WHERE CHILD PREVENTIVE AND CURATIVE CARE SERVICES ARE PROVIDED. FIND THE PERSON MOST KNOWLEDGEABLE ABOUT CHILD PREVENTIVE AND CURATIVE CARE SERVICES IN THE FACILITY. INTRODUCE YOURSELF, EXPLAIN THE PURPOSE OF THE SURVEY AND ASK THE FOLLOWING QUESTIONS. | | | | | | | |  |  |
| tHFA | B070 | RECORD THE DESIGNATION OF THE PERSON THAT YOU FOUND TO ANSWER QUESTIONS ABOUT CHILD PREVENTIVE AND CURATIVE CARE SERVICES. | | | | ____________________________________ | | | |  |  |
| R | i2004 | I would like to know if a document for child under 5 services is available in this service site today. Please show me the document on: | | | | | | | |  |  |
| R | 2005 _01 | National IMCI guidelines for diagnosis and management of childhood illnesses  [COUNTRY ADAPT – NAME OF DOCUMENT AND VERSION] | | | | YES, OBSERVED ............................................. 1  YES, REPORTED, NOT SEEN ........................... 2  NO/NOT AVAILABLE ...................................... 3 | | | |  |  |
| R | 2100 | Does this facility offer any immunization services, for adults or children? | | | | YES ….…………..………………………………………………1  NO ….……………………………………………………………2 | | | | ➔ B034 |  |
| R | i2103 | ASK TO BE SHOWN THE LOCATION IN THE FACILITY WHERE IMMUNIZATION SERVICES ARE PROVIDED. FIND THE PERSON MOST KNOWLEDGEABLE ABOUT IMMUNIZATION SERVICES IN THE FACILITY. INTRODUCE YOURSELF, EXPLAIN THE PURPOSE OF THE SURVEY AND ASK THE FOLLOWING QUESTIONS. | | | | | | | |  |  |
| tHFA | B071 | RECORD THE DESIGNATION OF THE PERSON THAT YOU FOUND TO ANSWER QUESTIONS ABOUT IMMUNIZATION SERVICES. | | | | ____________________________________ | | | |  |  |
| R | i2118 | I would like to know if a document for infant or child immunization is available in this service site today. Please show it to me. | | | | | | | |  |  |
| R | 2119 _01 | National guidelines for routine child immunization [COUNTRY ADAPT – NAME OF DOCUMENT AND VERSION] | | | | YES, OBSERVED ............................................. 1  YES, REPORTED, NOT SEEN ........................... 2  NO/NOT AVAILABLE ...................................... 3 | | | |  |  |
|  |  | ASK TO BE SHOWN IMMUNIZATION TALLEY SHEETS (OR ANOTHER INFORMATION SOURCE) THAT COULD BE USED TO DETERMINED THE NUMBER OF DOSES OF DPT VACCINE DELIVERED TO INFANTS (CHILDREN LESS THAN 12 MONTHS OLD) OVER THE PAST YEAR. REVIEW THE TALLEY SHEETS FOR DPT1 AND DPT3 VACCINE DOSES ADMINISTERED TO INFANTS IN THE LAST 4 COMPLETED CALENDAR QUARTERS. FOR EXAMPLE, IF THE SURVEY VISIT WAS ON AUGUST 1, 2023, THEN REVIEW THE DPT TALLEY SHEETS FOR JULY 2022 TO JUNE 2023 (I.E., THE LAST 4 COMPLETED CALENDAR QUARTERS ARE: JULY–SEPTEMBER 2022, OCTOBER–DECEMBER 2022, JANUARY–MARCH 2023, AND APRIL–JUNE 2023). | | | | | | | |  |  |
| tHFA | B032 | ENTER THE TOTAL NUMBER OF DPT1 DOSES ADMINISTERED AT THE FACILITY TO INFANTS (CHILDREN LESS THAN 12 MONTHS OLD) DURING THE LAST 4 COMPLETED CALENDAR QUARTERS. | | | | NUMBER OF DPT1 DOSES ____ ____ ____  ENTER “0” IF NO DPT1 DOSES WERE GIVEN.  ENTER “999” (I.E., DATA MISSING) IF DPT1 RECORDS CANNOT BE FOUND. | | | |  |  |
| tHFA | B033 | ENTER THE TOTAL NUMBER OF DPT3 DOSES ADMINISTERED AT THE FACILITY TO INFANTS (CHILDREN LESS THAN 12 MONTHS OLD) DURING THE LAST 4 COMPLETED CALENDAR QUARTERS. | | | | NUMBER OF DPT3 DOSES ____ ____ ____  ENTER “0” IF NO DPT3 DOSES WERE GIVEN.  ENTER “999” (I.E., DATA MISSING) IF DPT3 RECORDS CANNOT BE FOUND. | | | |  |  |
|  |  | AT THE END OF THE WORKING DAY (FOR EXAMPLE 17:00), FIND THE OUTPATIENT REGISTERS. | | | | | | | |  |  |
| tHFA | B034 | How many outpatients were seen at the facility yesterday?  ENTER THE DATE CORRESPONDING TO THE CASELOAD COUNT. | | | | NUMBER OF OUTPATIENTS ____ ____ ____  ENTER “0” IF NO OUTPATIENTS WERE SEEN  DATE (DD/MM/YYYY) ___/___/_____ | | | |  |  |
| tHFA | B058 | How many outpatients were seen at the facility during each of the past 5 weekdays (i.e., Monday–Friday)? ENTER “0” IF NO OUTPATIENTS WERE SEEN. | | | | DATE NUMBER  (DD/MM/YYYY) OF OUTPATIENTS  A) ___/___/______ ____ ____ ____  B) ___/___/______ ____ ____ ____  C) ___/___/______ ____ ____ ____  D) ___/___/______ ____ ____ ____  E) ___/___/______ ____ ____ ____ | | | |  |  |
|  |  | **END OF T-HFA SECTION 2** | | | | | | | |  |  |

| **Mod** | **No.** | **QUESTION** | | | | | **RESPONSE** | | | | | | | | **SKIP** |
| --- | --- | --- | --- | --- | --- | --- | --- | --- | --- | --- | --- | --- | --- | --- | --- |
|  |  | **T-HFA SECTION 3. Supervision**  Source: WHO 2022 HHFA Module 4: Management and finance (Core + Additional questionnaire), page 7.  Questions in this section are used to measure GF indicator KPI S2. | | | | | | | | | | | | |  |
|  |  | FIND THE PERSON MOST KNOWLEDGEABLE ABOUT EXTERNAL SUPERVISION RECEIVED BY THE HEALTH FACILITY. | | | | | | | | | | | | |  |
| tHFA | C012 | RECORD THE DESIGNATION OF THE PERSON THAT YOU FOUND TO ANSWER QUESTIONS ABOUT EXTERNAL SUPERVISION RECEIVED BY THE HEALTH FACILITY. | | | | | ____________________________________ | | | | | | | |  |
| M | 610 | Does this facility receive any external supervision, such as from district, regional or national offices? | | | | | YES ................................................................. 1  NO .................................................................. 2 | | | | | | | | ➔ End of section 3 |
| M | 611 | When was the last time a supervisor from outside this facility came here on a supervisory visit?  DO NOT INCLUDE VISITS WHERE GUESTS WERE BROUGHT OR THAT WERE FOR SUPPLIES ONLY. | | | | | WITHIN THE PAST 1 MONTH ........................... 1  2–3 MONTHS AGO .......................................... 2  4–12 MONTHS AGO ......................................... 3  MORE THAN 12 MONTHS AGO ....................... 4  DON’T KNOW .................................................. 8 | | | | | | | |  |
| tHFA |  | I will now ask some questions about which activities the supervisor did during the last external supervisory visit. I will name a type of health service. For that health service, please tell me if the supervisor did any of the following activities. USE THE CODE “2” IF THE INTERVIEWEE RESPONDS “NO” OR “DON’T KNOW.” | | | | | | | | | | | | |  |
| tHFA |  | START WITH HIV: ASK ABOUT EACH OF THE 6 ACTIVITIES FOR HIV. THEN TB: ASK ABOUT THE 6 ACTIVITIES, AND SO ON. | A) HIV | | B) TB | | C) Malaria | | D) ANC | | E) Immuni-zations | | F) IMCI or child health | |  |
|  |  |  | Yes | No or don’t know | Yes | No or don’t know | Yes | No or don’t know | Yes | No or don’t know | Yes | No or don’t know | Yes | No or don’t know |  |
| tHFA | C001 | Review data (for example, data from patient or lab registers, commodity stock records, community data, or summary statistics) | 1 | 2 | 1 | 2 | 1 | 2 | 1 | 2 | 1 | 2 | 1 | 2 | If any C001 responses are “Yes”, then C007 should be “Yes” and C007 can be skipped. |
| tHFA | C002 | Observe clinical consultations or lab functions | 1 | 2 | 1 | 2 | 1 | 2 | 1 | 2 | 1 | 2 | 1 | 2 |  |
| tHFA | C003 | Give feedback to health workers | 1 | 2 | 1 | 2 | 1 | 2 | 1 | 2 | 1 | 2 | 1 | 2 |  |
| tHFA | C004 | Answer health workers’ questions | 1 | 2 | 1 | 2 | 1 | 2 | 1 | 2 | 1 | 2 | 1 | 2 |  |
| tHFA | C005 | Provide on-the-spot training for health workers (for example, training on a new guideline, or how to perform a clinical task) | 1 | 2 | 1 | 2 | 1 | 2 | 1 | 2 | 1 | 2 | 1 | 2 |  |
| tHFA | C006 | Discuss health activities that take place at the community level (for example, health education, household visits, or treatment of ill patients by community health workers) | 1 | 2 | 1 | 2 | 1 | 2 | 1 | 2 | 1 | 2 | 1 | 2 | If any C006 responses are “Yes”, then C011 should be “Yes” and C011 can be skipped. |
| tHFA | C007 | During the last external supervisory visit, were summary statistics related to program performance and quality presented and discussed? Summary statistics could be on any health area from any of the following sources: patient or lab registers, commodity stock records, community data or community activities, or other facility data. The supervisor did not have to give the presentation; the supervisor only needed to participate in the discussion. | | | | | YES ................................................................. 1  NO .................................................................. 2  DON’T KNOW …………………………………………….. 8 | | | | | | | |  |
| tHFA | C008 | During the last external supervisory visit, did the supervisor facilitate group problem-solving? Problem-solving means the supervisor was part of a formal or informal meeting or discussion in which a specific problem was identified and possible solutions to the problem were discussed. Group problem-solving means that the meeting or discussion involved at least two facility staff members. | | | | | YES ................................................................. 1  NO .................................................................. 2  DON’T KNOW …………………………………………….. 8 | | | | | | | | ➔ C010  ➔ C010 |
| tHFA | C009 | Did the group problem-solving activity include a review of any kind of health data, such as: patient or lab registers, commodity stock records, community data or community activities, or summary statistics? | | | | | YES ................................................................. 1  NO .................................................................. 2  DON’T KNOW …………………………………………….. 8 | | | | | | | |  |
| tHFA | C010 | Are there community health workers that work with this health facility or that report to this facility or that get support from this facility? | | | | | YES ................................................................. 1  NO .................................................................. 2  DON’T KNOW …………………………………………….. 8 | | | | | | | | ➔ End of section  ➔ End of section |
| tHFA | C011 | During the last external supervisory visit, did the supervisor review and discuss data on community-level activities provided by community health workers? This could include a simple discussion about which community activities had occurred in the preceding several months that involved community health workers. | | | | | YES ................................................................. 1  NO .................................................................. 2  DON’T KNOW …………………………………………….. 8 | | | | | | | |  |
|  |  | **END OF T-HFA SECTION 3** | | | | | | | | | | | | |  |

| **Mod** | **No.** | **QUESTION** | | **RESPONSE** | | **SKIP** |
| --- | --- | --- | --- | --- | --- | --- |
|  |  | **T-HFA SECTION 4. PATIENT EXIT INTERVIEWS**  Source: WHO (2023). Patient-reported experiences in primary care: Patient questionnaire. Draft.  Questions in this section are used to measure GF indicator KPI S1. | | | |  |
|  |  | ASK WHERE PATIENTS TYPICALLY LEAVE THE HEALTH FACILITY AT THE END OF THEIR VISIT TO THE FACILITY. GO THERE. SELECT THE FIRST PERSON LEAVING THE FACILITY, INTRODUCE YOURSELF, AND BEGIN THE SURVEY PROCESS (SEE STEPS BELOW). WHEN THE SURVEY PROCESS IS DONE (WHICH MIGHT OR MIGHT NOT RESULT IN A COMPLETED PATIENT EXIT INTERVIEW), SELECT THE VERY NEXT PERSON LEAVING THE FACILITY (I.E., DO NOT WAIT FOR A PERSON THAT APPEARS TO BE A PATIENT OR THAT APPEARS TO BE EASY TO INTERVIEW), INTRODUCE YOURSELF, AND BEGIN THE SURVEY PROCESS, AND SO ON. CONTINUE THIS PROCESS UNTIL FIVE PATIENT EXIT INTERVIEWS HAVE BEEN COMPLETED (I.E., FIVE PATIENTS THAT WERE ELIGIBLE **AND** GAVE CONSENT **AND** ANSWERED AT LEAST ONE QUESTION ON PATIENT EXPERIENCE OF CARE). | | | |  |
|  |  | ASSESS ELIGIBILITY OF EACH PERSON THAT YOU SELECT WHO IS LEAVING THE FACILITY. | | | |  |
|  |  | Hello, my name is [surveyor], and I am surveyor. Did you come to this health facility to seek health care? | | YES ................................................................. 1  NO .................................................................. 2 | | ➔ Thank the person and select the very next person leaving the facility |
|  |  | Did you have a consultation with a health worker during your visit to the health facility today? | | YES ................................................................. 1  NO .................................................................. 2 | | ➔ Thank the person and select the very next person leaving the facility |
|  |  | CONSENT | | | |  |
|  |  | The [survey manager and survey implementer] in close collaboration with the [other relevant entities] are working to collect information from patients about health services. The survey is part of the [government’s] ongoing efforts to understand the quality of health services. I would now like to ask you some questions about your visit to the health facility today. This may take from 10 to 15 minutes. I will not record your name, and your responses will be kept confidential. Your participation and your responses will not affect the care you might receive at this health facility in the future. Your participation in this survey is voluntary. You may choose not to participate at all, and you may stop at any time before the end of the survey. You may choose not to answer any question that you are not comfortable with. In case you have any questions about this survey at any time, please feel free to contact any of the following people:  [LIST NAMES AND PHONE NUMBERS OF SURVEY MANAGEMENT PERSONS WHO CAN BE CONTACTED]  At this point do you have any questions about the study? Do I have your agreement to proceed? | | | |  |
|  |  | Signature of surveyor indicating informed consent was read and agreed by the patient or patient’s caregiver, if the patient is a child (sign on the line above) |  | | Signature of patient (or patient’s caregiver, if the patient is a child) authorizing data collection (sign on the line above) |  |
|  |  | Consent given by patient or patient’s caregiver, if the patient is a child? | | YES ................................................................. 1  NO .................................................................. 2 | | ➔ Thank the very person and select the next person leaving the facility |
| PRE | Q132 | During your visit to the health facility today, did your primary care professionals speak to you in a way you could understand?  READ THE FIRST FIVE RESPONSES. | | NEVER .............................................................. 1  RARELY ............................................................. 2  SOMETIMES ..................................................... 3  OFTEN .............................................................. 4  ALWAYS ........................................................... 5  DON’T KNOW .................................................. 8 | |  |
| PRE | Q122 | During your visit to the health facility today, did your primary care professionals treat you with respect?  READ THE FIRST FIVE RESPONSES. | | NEVER .............................................................. 1  RARELY ............................................................. 2  SOMETIMES ..................................................... 3  OFTEN .............................................................. 4  ALWAYS ........................................................... 5  DON’T KNOW .................................................. 8 | |  |
| tHFA | D006 | Thinking about your visit today, did you feel that any of the workers at the health facility shouted at you, scolded you, insulted you, threatened you, or talked to you rudely? | | YES ................................................................... 1  NO ................................................................... 2  DON’T KNOW ………………………………………………. 8 | |  |
| PRE | Q125 | During your visit to the health facility today, did you feel like you were involved in making decisions about your care as much as you wanted?  READ THE FIRST FIVE RESPONSES. | | NEVER .............................................................. 1  RARELY ............................................................. 2  SOMETIMES ..................................................... 3  OFTEN .............................................................. 4  ALWAYS ........................................................... 5  DON’T KNOW .................................................. 8 | |  |
| PRE | Q119 | During your visit to the health facility today, did you believe your primary care professionals would keep your information confidential?  READ THE FIRST FIVE RESPONSES. | | NEVER .............................................................. 1  RARELY ............................................................. 2  SOMETIMES ..................................................... 3  OFTEN .............................................................. 4  ALWAYS ........................................................... 5  DON’T KNOW .................................................. 8 | |  |
| PRE | Q129 | During your visit to the health facility today, did your primary care professionals discuss with you the care and support you receive from family members, friends, and others?  READ THE FIRST FIVE RESPONSES. | | NEVER .............................................................. 1  RARELY ............................................................. 2  SOMETIMES ..................................................... 3  OFTEN .............................................................. 4  ALWAYS ........................................................... 5  DON’T KNOW .................................................. 8 | |  |
| tHFA | D001 | What is your age (or the child’s age if the patient is a child)? | | PATIENT’S AGE ____ ____ ____ YEARS  ENTER “999” FOR DON’T KNOW | |  |
| tHFA | D002 | Record the patient’s sex | | MALE …………………………………………………………… 1  FEMALE ……………………………………………………….. 2  DON’T KNOW ………………………………………………. 8 | |  |
| tHFA | D003 | During your visit to the health facility today, did your primary care professionals tell you what illness you/your child has? | | YES ................................................................. 1  NO .................................................................. 2  DON’T KNOW …………………………………………….. 8 | |  |
| tHFA | D004 | During your visit to the health facility today, did your primary care professionals tell you about any illness signs or symptoms for which you must immediately come or bring the child back to a health facility? | | YES ................................................................. 1  NO .................................................................. 2  DON’T KNOW …………………………………………….. 8 | | ➔ D011  ➔ D011 |
| tHFA | D005 | IF YES, ASK: Can you tell me one of these signs or symptoms? | | FEVER ............................................................... 1  BREATHING PROBLEMS.................................... 2  BECOMES SICKER ………………………………………… 3  BLOOD IN STOOL …………………………………………. 4  UNABLE TO EAT OR DRINK ..……………………..….. 5  CONVULSION ..……………………………………………… 6  OTHER …………………………………………………………. 7  DON’T KNOW ………………………………………………. 8 | |  |
| tHFA | D011 | What are the key challenges/issues that you face while seeking care at this facility?  Do not read response options. Multiple options allowed.  IF THE CLIENT RESPONDS THAT THERE WERE NO PROBLEMS, THEN SELECT CHOICE “OTHER”, AND SPECIFY “NO PROBLEMS”. | | A) TOO EXPENSIVE  B) TOO FAR  C) LONG WAITING TIME  D) FACILITY OFTEN CLOSED  E) HEALTH WORKERS NOT AVAILABLE  F) HEALTH WORKERS NOT RESPECTFUL  G) LACK OF TRANSPORT TO REACH FACILITY  H) POOR QUALITY OF CARE  J) OTHERS: _____________________________  J1) SPECIFY  K) DON’T KNOW | |  |
|  |  | THANK THE PATIENT. IF FEWER THAN FIVE PATIENT EXIT INTERVIEWS HAVE BEEN COMPLETED, THEN SELECT THE VERY NEXT PERSON LEAVING THE FACILITY. | | | |  |
|  |  | **END OF T-HFA SECTION 4** | | | |  |

| **Mod** | **No.** | **QUESTION** | | **RESPONSE** | | **SKIP** |
| --- | --- | --- | --- | --- | --- | --- |
|  |  | **T-HFA SECTION 5. COMMUNITY HEALTH WORKER INTERVIEWS**  Questions in this section are used to measure GF indicator KPI S5 and RSSH/PP HRH-3. | | | |  |
| tHFA | E031 | Are there community health workers providing health services in the catchment area of this facility? | | YES ................................................................... 1  NO .................................................................... 2 | | ➔ End of section 5 |
| tHFA | CHW_1 | Are any of the randomly selected community health workers present at the facility for the interview and give consent? | | YES ................................................................... 1  NO .................................................................... 2 | | ➔ End of section 5 |
| tHFA | CHW_2 | How many community health workers Present at the facility for the interview? | | …………………………………NUMBER OF COMMUNITY HEALTH WORKERS PRESENT | |  |
|  |  | TO SELECT CHWS, THE SURVEY TEAM WILL CONTACT THE FACILITY AT LEAST ONE WEEK BEFORE THE FACILITY VISIT AND DO THE FOLLOWING: 1) ASK HOW MANY “RECO site” CHWS ARE ASSOCIATED WITH THE FACILITY, AND RECORD THEIR NAMES ON AN ENUMERATED LIST; 2) RANDOMLY SELECT 5 RECO site CHWS FROM THE LIST; AND 3) ASK THE FACILITY STAFF TO INVITE THE 5 RANDOMLY SELECTED CHWS TO COME THE FACILITY ON THE DAY OF THE HFA VISIT TO BE INTERVIEWED. ASK THE INVITED CHWS TO BRING ANY RECORDS THEY KEEP ON THE QUALITY OF CARE THAT THEY PROVIDE TO CLIENTS WITH HIV, TB, OR MALARIA (FOR EXAMPLE, CLIENT REGISTER BOOKS). IF CHWS ARE EMPLOYED BY A CIVIL SOCIETY ORGANIZATION (CSO) OR OTHER ORGANIZATION (SUCH AS SANRU), THEN THE SURVEY TEAM WILL CONTACT THAT CSO OR OTHER ORGANIZATION AND PERFORM THE ABOVE THREE STEPS.  IF, ON THE DAY OF THE HFA VISIT, THERE IS AT LEAST 1 CHW THAT GIVES CONSENT TO PARTICIPATE IN THE SURVEY, THEN COLLECT THE INFORMATION FROM THE CHW(S) VIA AN INTERVIEW. IF THERE ARE NO CHWS THAT ARE PRESENT AND THAT GIVE CONSENT, THEN COLLECT THE INFORMATION FROM THE FACILITY IN-CHARGE, ANOTHER FACILITY STAFF MEMBER THAT INTERACTS WITH THE CHWS, OR (IF CHWS ARE EMPLOYED BY A CSO, SUCH AS SANRU) A CSO STAFF PERSON WHO INTERACTS WITH CHWS. SURVEYORS WILL RECORD THE DATA SOURCE—THAT IS, INTERVIEW WITH CHW(S) OR INTERVIEW WITH A NON-CHW (SUCH AS, A FACILITY IN-CHARGE OR CSO STAFF PERSON). | | | |  |
|  |  | ASK TO MEET WITH THE COMMUNITY HEALTH WORKERS.  ***Note on the questionnaire structure. This section of the questionnaire (below) shows one set of questions for each CHW interview. When the t-HFA questionnaire (either paper or electronic version) is created for field use, these questions will be repeated five times, for the goal of interviewing five CHWs per facility.*** | | | |  |
| tHFA | E001 | ASSESS ELIGIBILITY OF CHW.  Hello, my name is [surveyor], and I am surveyor. Are you a RECO site community health worker that works in communities around this health facility? | | YES ................................................................. 1  NO .................................................................. 2 | | ➔ Thank the CHW and select the next CHW |
|  |  | CONSENT | | | |  |
|  |  | The [survey manager and survey implementer] in close collaboration with the [other relevant entities] are working to collect information from community health workers about health services. The survey is part of the [government’s] ongoing efforts to understand the quality of health services. I would now like to ask you questions about your work. This may take from 10 to 15 minutes. I will not record your name, and your responses will be kept confidential. Your participation in this survey is voluntary. You may choose not to participate at all or to stop at any time before the end of the survey. You may also choose not to answer any question that you are not comfortable with. In case you have any question(s) about this survey at any time, please feel free to contact any of the following people:  [LIST NAMES AND PHONE NUMBERS OF SURVEY MANAGEMENT PERSONS WHO CAN BE CONTACTED]  At this point do you have any questions about the study? Do I have your agreement to proceed? | | | |  |
|  |  | Signature of surveyor indicating informed consent was read and agreed by the CHW (sign on the line above) |  | | Signature of CHW authorizing data collection (sign on the line above) |  |
| tHFA | E002 | Consent given by the CHW? | | YES ................................................................. 1  NO .................................................................. 2 | | ➔ Thank CHW and select the next CHW |
|  |  | IF AT LEAST ONE CHW HAS GIVEN CONSENT, THEN ASK ALL CONSENTED CHWS THE FOLLOWING QUESTIONS. IF NO CHW HAS GIVEN CONSENT, THEN ASK THE QUESTIONS TO THE FACILITY STAFF PERSON WHO IS MOST FAMILIAR WITH CHWS. IF CHWS ARE EMPLOYED BY A CSO, THEN ADMINISTER THE QUESTIONS TO A CSO STAFF PERSON WHO IS FAMILIAR WITH CHWS. | | | |  |
| tHFA | E033 | What is your gender? | | MALE …………………………………………………………… 1  FEMALE ………………………………………………….……. 2  OTHER …………………………………………………………. 3  CHW DOES NOT RESPOND …………………………… 4 | |  |
| tHFA | E003 | I would now like to ask you about your work responsibilities. For each of the following health areas, tell me if you have work responsibilities. | | A) HIV  YES .................................................................. 1  NO .................................................................. 2  DON’T KNOW OR NOT SURE ……………………….. 8    B) Tuberculosis/TB  YES .................................................................. 1  NO .................................................................. 2  DON’T KNOW OR NOT SURE ……………………….. 8    C) Malaria  YES .................................................................. 1  NO .................................................................. 2  DON’T KNOW OR NOT SURE ……………………….. 8    D) Nutrition  YES .................................................................. 1  NO .................................................................. 2  DON’T KNOW OR NOT SURE ……………………….. 8    E) Maternal, newborn, or child health  YES .................................................................. 1  NO .................................................................. 2  DON’T KNOW OR NOT SURE ……………………….. 8 | |  |
|  |  | I would now like to ask you about supervision visits that you received. Please think about supervision visits during the months of X, Y, and Z. THE MONTHS X, Y, AND Z REFER TO THE LAST COMPLETED CALENDAR QUARTER. FOR EXAMPLE, A CHW INTERVIEW CONDUCTED ON AUGUST 1 WOULD ASK ABOUT SUPERVISION DURING THE PRECEDING APRIL–JUNE QUARTER.  ***Note on the questionnaire structure. For questions E005, E007, and E009, only ask about health areas that are the CHW’s responsibilities, according to responses to question E003.*** | | | |  |
| tHFA | E004 | Did you receive a supervision visit last [month X]?  IN THE ABOVE EXAMPLE, THIS WOULD BE “LAST APRIL”. | | YES .................................................................. 1  NO .................................................................. 2  DON’T KNOW OR NOT SURE ……………………….. 8 | | ➔ E006  ➔ E006 |
| tHFA | E005 | IF YES, ASK:  Did that supervision visit include activities or discussions about any of the following health areas? | | A) HIV  YES .................................................................. 1  NO .................................................................. 2  DON’T KNOW OR NOT SURE ……………………….. 8  B) Tuberculosis/TB  YES .................................................................. 1  NO .................................................................. 2  DON’T KNOW OR NOT SURE ……………………….. 8  C) Malaria  YES .................................................................. 1  NO .................................................................. 2  DON’T KNOW OR NOT SURE ……………………….. 8  D) Nutrition  YES .................................................................. 1  NO .................................................................. 2  DON’T KNOW OR NOT SURE ……………………….. 8  E) Maternal, newborn, or child health  YES .................................................................. 1  NO .................................................................. 2  DON’T KNOW OR NOT SURE ……………………….. 8 | |  |
| tHFA | E006 | Did you receive a supervision visit last [month Y]?  IN THE ABOVE EXAMPLE, THIS WOULD BE “LAST MAY”. | | YES .................................................................. 1  NO .................................................................. 2  DON’T KNOW OR NOT SURE ……………………….. 8 | | ➔ E008  ➔ E008 |
| tHFA | E007 | IF YES, ASK:  Did that supervision visit include activities or discussions about any of the following health areas? | | A) HIV  YES .................................................................. 1  NO .................................................................. 2  DON’T KNOW OR NOT SURE ……………………….. 8  B) Tuberculosis/TB  YES .................................................................. 1  NO .................................................................. 2  DON’T KNOW OR NOT SURE ……………………….. 8  C) Malaria  YES .................................................................. 1  NO .................................................................. 2  DON’T KNOW OR NOT SURE ……………………….. 8  D) Nutrition  YES .................................................................. 1  NO .................................................................. 2  DON’T KNOW OR NOT SURE ……………………….. 8  E) Maternal, newborn, or child health  YES .................................................................. 1  NO .................................................................. 2  DON’T KNOW OR NOT SURE ……………………….. 8 | |  |
| tHFA | E008 | Did you receive a supervision visit last [month Z]?  IN THE ABOVE EXAMPLE, THIS WOULD BE “LAST JUNE”. | | YES .................................................................. 1  NO .................................................................. 2  DON’T KNOW OR NOT SURE ……………………….. 8 | | ➔ E010  ➔ E010 |
| tHFA | E009 | IF YES, ASK:  Did that supervision visit include activities or discussions about any of the following health areas? | | A) HIV  YES .................................................................. 1  NO .................................................................. 2  DON’T KNOW OR NOT SURE ……………………….. 8  B) Tuberculosis/TB  YES .................................................................. 1  NO .................................................................. 2  DON’T KNOW OR NOT SURE ……………………….. 8  C) Malaria  YES .................................................................. 1  NO .................................................................. 2  DON’T KNOW OR NOT SURE ……………………….. 8  D) Nutrition  YES .................................................................. 1  NO .................................................................. 2  DON’T KNOW OR NOT SURE ……………………….. 8  E) Maternal, newborn, or child health  YES .................................................................. 1  NO .................................................................. 2  DON’T KNOW OR NOT SURE ……………………….. 8 | |  |
| tHFA | E010 | I would now like to ask you about your RECO site community health worker contract. A contract is a written agreement about your work as a RECO site community health worker that has been signed by you and your employer. Do you have a contract? | | YES .................................................................. 1  NO .................................................................. 2  DON’T KNOW OR NOT SURE ……………………….. 8 | | ➔ E024  ➔ E024 |
|  |  | Does your contract include the following: | |  | |  |
| tHFA | E011 | A description of your work responsibilities? | | YES .................................................................. 1  NO .................................................................. 2  DON’T KNOW OR NOT SURE ……………………….. 8 | |  |
| tHFA | E012 | The expected number of hours that you must work each day, week, or month? | | YES .................................................................. 1  NO .................................................................. 2  DON’T KNOW OR NOT SURE ……………………….. 8 | |  |
| tHFA | E013 | A level of financial remuneration that is at least the national minimum wage? | | YES .................................................................. 1  NO .................................................................. 2  DON’T KNOW OR NOT SURE ……………………….. 8 | |  |
| tHFA | E014 | A description of how often you will be paid (for example, monthly)? | | YES .................................................................. 1  NO .................................................................. 2  DON’T KNOW OR NOT SURE ……………………….. 8 | |  |
| tHFA | E015 | Rest days, or days that you do not need to work (for example, weekends)? | | YES .................................................................. 1  NO .................................................................. 2  DON’T KNOW OR NOT SURE ……………………….. 8 | |  |
| tHFA | E016 | Annual leave or vacation days (for example, two weeks per year)? | | YES .................................................................. 1  NO .................................................................. 2  DON’T KNOW OR NOT SURE ……………………….. 8 | |  |
| tHFA | E017 | Paid sick leave? | | YES .................................................................. 1  NO .................................................................. 2  DON’T KNOW OR NOT SURE ……………………….. 8 | |  |
| tHFA | E018 | Holidays? | | YES .................................................................. 1  NO .................................................................. 2  DON’T KNOW OR NOT SURE ……………………….. 8 | |  |
| tHFA | E019 | Health insurance? | | YES .................................................................. 1  NO .................................................................. 2  DON’T KNOW OR NOT SURE ……………………….. 8 | |  |
| tHFA | E020 | Do you receive payment for your work as a RECO site community health worker? | | YES .................................................................. 1  NO .................................................................. 2  DON’T KNOW OR NOT SURE ……………………….. 8 | | ➔ E024  ➔ E024 |
|  |  | I would now like to ask about your pay or salary. Please think about your payments during the months of X, Y, and Z. THE MONTHS X, Y, AND Z REFER TO THE LAST COMPLETED CALENDAR QUARTER. FOR EXAMPLE, A CHW INTERVIEW CONDUCTED ON AUGUST 1 WOULD ASK ABOUT SUPERVISION DURING THE PRECEDING APRIL–JUNE QUARTER. | | | |  |
| tHFA | E021 | For all 3 of the months, were you paid the amount stated in your contract? | | YES .................................................................. 1  NO .................................................................. 2  DON’T KNOW OR NOT SURE ……………………….. 8 | |  |
| tHFA | E022 | For all 3 of the months, were you paid according to the frequency stated in your contract (for example, each month)? | | YES .................................................................. 1  NO .................................................................. 2  DON’T KNOW OR NOT SURE ……………………….. 8 | |  |
| tHFA | E023 | For all 3 of the months, were you paid on time? | | YES .................................................................. 1  NO .................................................................. 2  DON’T KNOW OR NOT SURE ……………………….. 8 | |  |
| tHFA | E032 | How much are you paid for your work as a community health worker? PROMPT CHW FOR SPECIFIC QUESTION ELEMENTS. | | A) Amount __________________  B) Currency _________________  C) Time period. CHW receives payment each:  Day …………………………………………………………. 1  Week ………………………………………………………. 2  Month …………………………………………………….. 3  Quarter (3 months) …………………………………. 4  Other ………………………………………………………. 5  CHW does not receive regular payment ….. 6  CHW refuses to respond ………………………….. 7  D) Specify “other”: __________________ | |  |
|  |  | I would now like to ask about the things that you need to do your work as a RECO site community health worker. Please think about the months of X, Y, and Z. THE MONTHS X, Y, AND Z REFER TO THE LAST COMPLETED CALENDAR QUARTER. FOR EXAMPLE, A CHW INTERVIEW CONDUCTED ON AUGUST 1 WOULD ASK ABOUT SUPERVISION DURING THE PRECEDING APRIL–JUNE QUARTER. | | | |  |
| tHFA | E024 | What is the most important commodity that you need for your work as a RECO site community health worker? A commodity is an item that gets used up (for example, medicines, diagnostic tests, or condoms). CHOOSE ONE RESPONSE. | | Medicines …………………………………………………… 1  Diagnostic tests ………………………………………….. 2  Condoms ……………………………………………………. 3  Other …………………………………………………………. 4 | |  |
| tHFA | E025 | Regarding the commodity that you just mentioned, did you always have the item in-stock during the months [X, Y, and Z]? | | YES .................................................................. 1  NO .................................................................. 2  DON’T KNOW OR NOT SURE ……………………….. 8 | |  |
| tHFA | E026 | What is the most important piece of equipment that you need for your work as a RECO site community health worker? Examples of equipment are a backpack or box to carry things, a thermometer, or a scale to weigh children. CHOOSE ONE RESPONSE. | | Backpack …………………………………………………….. 1  Raingear ……………………………………………….…….. 2  Boots ………………………………………………………….. 3  Jacket …………………………………………………………. 4  Scale ………………………………………………………….. 5  Timer or stopwatch ……………………………………. 6  Security box (e.g., to store medicines) ……….. 7  Bicycle ……………………………………………………..… 8  Writing utensil …………………………………………… 9  Phone ……………………………………………………….. 10  Other ………………………………………………………… 11 | |  |
| tHFA | E027 | Regarding the item that you just mentioned, did you have the item and was it always functioning properly during the months [X, Y, and Z]? | | YES .................................................................. 1  NO .................................................................. 2  DON’T KNOW OR NOT SURE ……………………….. 8 | |  |
| tHFA | E028 | What is the most important job aid or form that you need for your work as a RECO site community health worker? Examples of job aids or forms are a patient register, a monthly reporting form, a counseling flip chart, or a guideline that explains how to do your job. CHOOSE ONE RESPONSE. | | Patient register ……………………………………………. 1  Monthly reporting form ………………………………. 2  Counseling flip chart ……………………………………. 3  Guideline that explains how to do your job …. 4  Other ………………………………………………………….. 5 | |  |
| tHFA | E029 | Regarding the item that you just mentioned, did you have the item during the months [X, Y, and Z]? | | YES .................................................................. 1  NO .................................................................. 2  DON’T KNOW OR NOT SURE ……………………….. 8 | |  |
| tHFA | E030 | SELECT THE RESPONSE THAT DESCRIBES WHO ANSWERED THE ABOVE CHW QUESTIONS | | A CHW ............................................................. 1  A NON-CHW (E.G., A CSO STAFF PERSON) ...... 2 | |  |
|  |  | THANK THE CHW. IF FEWER THAN FIVE CHW INTERVIEWS HAVE BEEN COMPLETED, THEN INTERVIEW THE NEXT CHW. | | | |  |
|  |  | **END OF T-HFA SECTION 5** | | | |  |

| **Mod** | **No.** | **QUESTION** | | | **RESPONSE** | | | | **SKIP** |
| --- | --- | --- | --- | --- | --- | --- | --- | --- | --- |
|  |  | **T-HFA SECTION 5A. RECORD REVIEW FOR “RELAIS COMMUNAUTAIRE SITE” (RECO SITE)**  **(FOR DRC ONLY)**  Questions in this section are used to measure GF indicator RSSH/PP HRH-4. | | | | | | |  |
|  |  | FOR THIS T-HFA SECTION, THE GOAL IS TO COLLECT DATA FROM CLIENT REGISTER BOOKS OF 5 “RELAIS COMMUNAUTAIRE SITE” (RECO SITE) CHWS THAT ARE ASSOCIATED WITH THE FACILITY VISITED TODAY. THESE ARE THE SAME RECO SITE CHWS THAT WERE INTERVIEWED FOR QUESTIONS IN SECTION 5, ABOVE. | | | | | | |  |
| tHFA | EA03 | HOW MANY RECO SITE COMMUNITY HEALTH WORKERS ARE PRESENT AT THE FACILITY FOR THE INTERVIEW AND RECORD REVIEW AND HAVE GIVEN THEIR CONSENT? | | | ……………………… NUMBER OF RECO SITE COMMUNITY HEALTH WORKERS WHO ARE PRESENT AND GAVE THEIR CONSENT | | | | 🡺 If zero RECO Site CHWs, then skip to Section 5B |
|  |  | ***Note on the questionnaire structure. This section of the questionnaire (below) shows one set of questions for each CHW record review. When the t-HFA questionnaire (either paper or electronic version) is created for field use, these questions will be repeated five times, for the goal of collecting data from the records of five CHWs per facility.*** | | | | | | |  |
|  |  |  | | |  | | | |  |
|  |  | **RECORD REVIEW FOR “RELAIS COMMUNAUTAIRE SITE” (RECO SITE) CHWS**  **ASK THE FOLLOWING QUESTIONS TO EACH OF THE (UP TO) 5 RECO SITE CHWS.** | | | | | | |  |
| tHFA | EA05 | ASK TO SEE THE RECO SITE CHW’S CLIENT REGISTER BOOK. SEE PAGE 25 OF THE T-HFA ANNEX 1 FOR AN IMAGE OF THE CORRECT REGISTER. SELECT THE RESPONSE THAT INDICATES WHETHER OR NOT THE CHW BROUGHT THE CLIENT REGISTER BOOK. | | | YES (CHW BROUGHT REGISTER BOOK) ........... 1  NO (CHW DID NOT BRING REGISTER BOOK) ... 2 | | | | 🡺 Last question for that RECO site CHW. Ask the next RECO site CHW. After the last RECO site CHW, skip to Section 5B. |
|  |  | **RECORD REVIEW FOR RECO SITE CHWS: TUBERCULOSIS** | | | | | | |  |
|  |  | **INSTRUCTIONS FOR SELECTING CLIENT RECORDS**  **ELIGIBILITY CRITERIA:** THE CLIENT HAD A HOUSEHOLD EXPOSURE TO A TB CASE PATIENT — I.E., COLUMN 16 OF THE RECO SITE CHW CLIENT REGISTER BOOK (“*NOTION DE CONTAGE TB*”) HAS A CHECK MARK. IF COLUMN 16 IS BLANK OR HAS AN “X” OR HAS ANYTHING WRITTEN THAT IS NOT A CHECK MARK, THEN THE CLIENT RECORD IS INELIGIBLE. IF YOU ARE UNSURE ABOUT WHAT IS WRITTEN IN THE REGISTER, ASK THE RECO SITE CHW.  **SAMPLE SELECTION:**  THE RECORD REVIEW REQUIRES TWO STEPS: (1) IDENTIFYING A SAMPLE OF ELIGIBLE CLIENT RECORDS, AND (2) ASSESSING THE ELIGIBLE RECORDS.  ***1. IDENTIFYING A SAMPLE OF ELIGIBLE CLIENT RECORDS:***  EXAMINE THE RECO SITE CHW’S CLIENT REGISTER BOOK AND FIND THE MOST RECENT COMPLETE MONTH WHERE CLIENTS CAN BE IDENTIFIED. COUNTING BACKWARDS FROM THE END OF THE MOST RECENT COMPLETE MONTH, IDENTIFY 10 CLIENTS WHO MEET THE ELIGIBILITY CRITERIA FOR THIS RECORD REVIEW. CONTINUE COUNTING ALL ELIGIBLE CLIENTS IN EACH PRECEDING MONTH TO A MAXIMUM OF 6 FULL MONTHS, IF NEEDED, TO IDENTIFY 10 CLIENTS MEETING THE ELIGIBILITY CRITERIA. DO NOT EXCEED SIX MONTHS EVEN IF 10 CLIENTS HAVE NOT BEEN IDENTIFIED. COMPLETE A CLIENT SAMPLING LIST (SEE ANNEX 3 “RECORD REVIEW SAMPLING TOOL”) BY RECORDING IDENTIFYING INFORMATION FOR THE ELIGIBLE CLIENTS. THE GOAL IS TO IDENTIFY 10 ELIGIBLE CLIENTS.  **IF 10 ELIGIBLE CLIENTS HAVE BEEN IDENTIFIED:** THE COMPLETED CLIENT SAMPLING LIST HAS A SAMPLING NUMBER BETWEEN 1 AND 10 FOR EACH CLIENT. TO SELECT THE 5 CLIENTS WHOSE INFORMATION WILL BE ENTERED INTO THE T-HFA QUESTIONNAIRE, TOSS A COIN. ONE SIDE WILL MEAN YOU SELECT CLIENTS WITH SAMPLING NUMBERS 1, 3, 5, 7, AND 9; THE OTHER SIDE WILL MEAN YOU SELECT CLIENTS 2, 4, 6, 8, AND 10.  **IF FEWER THAN 10 ELIGIBLE CLIENTS HAVE BEEN IDENTIFIED:** TO SELECT THE 5 CLIENTS WHOSE INFORMATION WILL BE ENTERED INTO THE T-HFA QUESTIONNAIRE, FOLLOW THE INSTRUCTIONS IN THE RECORD REVIEW SAMPLING TOOL (ANNEX 3).  ***2. ASSESSING THE ELIGIBLE RECORDS:***  EXAMINE THE RECO SITE CHW’S CLIENT REGISTER BOOK FOR EACH OF THE 5 CLIENTS SELECTED FOR THE SAMPLE AND ANSWER THE QUESTIONS BELOW. | | | | | | |  |
|  |  | FOR THE NEXT 2 QUESTIONS, EXAMINE THE RECO SITE CHW’S CLIENT REGISTER BOOK. EACH QUESTION CORRESPONDS TO A DIFFERENT COLUMN. EACH COLUMN CAN BE IDENTIFIED IN TWO WAYS. FIRST, BY A NUMBER, WHICH IS NOT SHOWN IN THE CLIENT REGISTER BOOK, BUT CAN BE DETERMINED BY COUNTING COLUMNS FROM THE LEFT SIDE OF THE REGISTER PAGE (E.G., COLUMN 16 IS THE SIXTEENTH COLUMN FROM THE LEFT). SECOND, BY A COLUMN HEADING, WHICH IS IN FRENCH IN THE REGISTER BOOK (AN ENGLISH TRANSLATION IS PROVIDED BELOW). FOR EACH CLIENT, SELECT A RESPONSE THAT INDICATES WHAT IS WRITTEN IN THE COLUMN FOR THE CLIENT. IF YOU ARE UNSURE ABOUT WHAT IS WRITTEN IN THE REGISTER, ASK THE RECO SITE CHW. ONLY SELECT THE “UNSURE” CHOICE IF IT IS GENUINELY IMPOSSIBLE TO INTERPRET WHAT IS WRITTEN IN THE REGISTER BOOK. | | | | | | |  |
|  |  |  | CLIENT 1 | CLIENT 2 | | CLIENT 3 | CLIENT 4 | CLIENT 5 | |
| tHFA | EA06 | COLUMN 16. “Notion de Contage TB” (i.e., household contact with a TB case patient). Was the response in column 16 a check mark? (i.e., the client had a household contact with a TB case patient) | YES ............. 1  NO ............. 2  UNSURE ….. 8 | YES ............. 1  NO ............. 2  UNSURE ….. 8 | | YES ............. 1  NO ............. 2  UNSURE ….. 8 | YES ............. 1  NO ............. 2  UNSURE ….. 8 | YES ............. 1  NO ............. 2  UNSURE ….. 8 | |
| tHFA | EA07 | COLUMN 66. "OBSERVATIONS.  O = orienté, OR = Orientation recours, G = Guerris” etc. (i.e., Disposition of the client. Referred, Cured, etc.)  Was the response in column 66 an “O” or “Orienté”? (i.e., CHW referred the client to a facility) | YES ............. 1  NO ............. 2  UNSURE ….. 8 | YES ............. 1  NO ............. 2  UNSURE ….. 8 | | YES ............. 1  NO ............. 2  UNSURE ….. 8 | YES ............. 1  NO ............. 2  UNSURE ….. 8 | YES ............. 1  NO ............. 2  UNSURE ….. 8 | |
|  |  |  | | |  | | | |  |
|  |  | **CHW RECORD REVIEW FOR RECO SITE CHWS: MALARIA** | | | | | | |  |
|  |  | **INSTRUCTIONS FOR SELECTING CLIENT RECORDS**  **ELIGIBILITY CRITERIA:** THE CLIENT HAD A FEBRILE ILLNESS — I.E., COLUMN 11 OF THE CLIENT REGISTER (“FIÈVRE (JRS)”, WHICH IS FRENCH FOR “FEVER (DAYS)”) HAS A NUMBER (WHICH MEANS THE NUMBER OF DAYS THAT THE CLIENT HAD FEVER). IF COLUMN 11 IS BLANK OR HAS AN ”X” OR HAS ANYTHING WRITTEN THAT IS NOT A NUMBER, THEN THE CLIENT RECORD IS INELIGIBLE.  **SAMPLE SELECTION:**  THE RECORD REVIEW REQUIRES TWO STEPS: (1) IDENTIFYING A SAMPLE OF ELIGIBLE CLIENT RECORDS, AND (2) ASSESSING THE ELIGIBLE RECORDS.  ***1. IDENTIFYING A SAMPLE OF ELIGIBLE CLIENT RECORDS:***  EXAMINE THE RECO SITE CHW’S CLIENT REGISTER BOOK AND FIND THE MOST RECENT COMPLETE MONTH WHERE CLIENTS CAN BE IDENTIFIED. COUNTING BACKWARDS FROM THE END OF THE MOST RECENT COMPLETE MONTH, IDENTIFY 10 CLIENTS WHO MEET THE ELIGIBILITY CRITERIA FOR THIS RECORD REVIEW. CONTINUE COUNTING ALL ELIGIBLE CLIENTS IN EACH PRECEDING MONTH TO A MAXIMUM OF 6 FULL MONTHS, IF NEEDED, TO IDENTIFY 10 CLIENTS MEETING THE ELIGIBILITY CRITERIA. DO NOT EXCEED SIX MONTHS EVEN IF 10 CLIENTS HAVE NOT BEEN IDENTIFIED. COMPLETE A CLIENT SAMPLING LIST (SEE ANNEX 3 “RECORD REVIEW SAMPLING TOOL”) BY RECORDING IDENTIFYING INFORMATION FOR THE ELIGIBLE CLIENTS. THE GOAL IS TO IDENTIFY 10 ELIGIBLE CLIENTS.  **IF 10 ELIGIBLE CLIENTS HAVE BEEN IDENTIFIED:** THE COMPLETED CLIENT SAMPLING LIST HAS A SAMPLING NUMBER BETWEEN 1 AND 10 FOR EACH CLIENT. TO SELECT THE 5 CLIENTS WHOSE INFORMATION WILL BE ENTERED INTO THE T-HFA QUESTIONNAIRE, TOSS A COIN. ONE SIDE WILL MEAN YOU SELECT CLIENTS WITH SAMPLING NUMBERS 1, 3, 5, 7, AND 9; THE OTHER SIDE WILL MEAN YOU SELECT CLIENTS 2, 4, 6, 8, AND 10.  **IF FEWER THAN 10 ELIGIBLE CLIENTS HAVE BEEN IDENTIFIED:** TO SELECT THE 5 CLIENTS WHOSE INFORMATION WILL BE ENTERED INTO THE T-HFA QUESTIONNAIRE, FOLLOW THE INSTRUCTIONS IN THE RECORD REVIEW SAMPLING TOOL (ANNEX 3).  ***2. ASSESSING THE ELIGIBLE RECORDS:***  EXAMINE THE RECO SITE CHW’S CLIENT REGISTER BOOK FOR EACH OF THE 5 CLIENTS SELECTED FOR THE SAMPLE AND ANSWER THE QUESTIONS BELOW. | | | | | | |  |
|  |  | FOR THE NEXT 18 QUESTIONS, EXAMINE THE RECO SITE CHW’S CLIENT REGISTER BOOK. EACH QUESTION CORRESPONDS TO A DIFFERENT COLUMN. EACH COLUMN CAN BE IDENTIFIED IN TWO WAYS. FIRST, BY A NUMBER, WHICH IS NOT SHOWN IN THE CLIENT REGISTER BOOK, BUT CAN BE DETERMINED BY COUNTING COLUMNS FROM THE LEFT SIDE OF THE REGISTER PAGE (E.G., COLUMN 9 IS THE NINTH COLUMN FROM THE LEFT). SECOND, BY A COLUMN HEADING, WHICH IS IN FRENCH IN THE REGISTER BOOK (AN ENGLISH TRANSLATION IS PROVIDED BELOW). FOR EACH CLIENT, SELECT A RESPONSE THAT INDICATES WHAT IS WRITTEN IN THE COLUMN FOR THE CLIENT. FOR EXAMPLE, IN MANY COLUMNS, THE CHW WILL RECORD EITHER A NUMBER OR AN “X”. THE “OTHER” CHOICE MEANS THAT WHAT IS WRITTEN IN THE COLUMN IS NONE OF THE PRECEDING CHOICES. FOR EXAMPLE, FOR COLUMN 9 (“COUGH/COLD”), THE PRIMARY CHOICES ARE A NUMBER OR AN “X”. IF THE CHW HAS WRITTEN (FOR EXAMPLE) A WORD THAT IS OBVIOUSLY NOT A NUMBER OR “COUGH” OR “COLD”, THEN SELECT THE “OTHER” CHOICE. IF YOU ARE UNSURE ABOUT WHAT IS WRITTEN IN THE REGISTER, ASK THE RECO SITE CHW. ONLY SELECT THE “UNSURE” CHOICE IF IT IS GENUINELY IMPOSSIBLE TO INTERPRET WHAT IS WRITTEN IN THE REGISTER BOOK. | | | | | | |  |
|  |  |  | CLIENT 1 | CLIENT 2 | | CLIENT 3 | CLIENT 4 | CLIENT 5 | |
| tHFA | EA08 | COLUMN 9. “Toux/Rhume (jrs)” (i.e., “Cough/Cold (days)”) | Any number … 1  “X” ………………. 2  Other …………… 3  Unsure ………… 4 | Any number … 1  “X” ………………. 2  Other …………… 3  Unsure ………… 4 | | Any number … 1  “X” ………………. 2  Other …………… 3  Unsure ………… 4 | Any number … 1  “X” ………………. 2  Other …………… 3  Unsure ………… 4 | Any number … 1  “X” ………………. 2  Other …………… 3  Unsure ………… 4 | |
| tHFA | EA09 | COLUMN 10. “Diarhée (jrs)”  (i.e., “Diarrhea (days)”) | Any number … 1  “X” ………………. 2  Other …………… 3  Unsure ………… 4 | Any number … 1  “X” ………………. 2  Other …………… 3  Unsure ………… 4 | | Any number … 1  “X” ………………. 2  Other …………… 3  Unsure ………… 4 | Any number … 1  “X” ………………. 2  Other …………… 3  Unsure ………… 4 | Any number … 1  “X” ………………. 2  Other …………… 3  Unsure ………… 4 | |
| tHFA | EA10 | COLUMN 11. “Fièvre (jrs)”  (i.e., “Fever (days)”) | Any number … 1  “X” ………………. 2  Other …………… 3  Unsure ………… 4 | Any number … 1  “X” ………………. 2  Other …………… 3  Unsure ………… 4 | | Any number … 1  “X” ………………. 2  Other …………… 3  Unsure ………… 4 | Any number … 1  “X” ………………. 2  Other …………… 3  Unsure ………… 4 | Any number … 1  “X” ………………. 2  Other …………… 3  Unsure ………… 4 | |
| tHFA | EA11 | COLUMN 17. "Signes d'orientation (Voir Carte de signe d'orientation pour < 5 ans ou ≥ 5 ans et plus)" (i.e., “Referral signs”)  FOR THIS QUESTION, ASK THE RECO SITE CHW IF THE CLIENT HAD ANY SIGNS THAT INDICATED THAT THE CLIENT NEEDED TO BE REFERRED TO A FACILITY  (I.E., RESPONSE “1”). | Any number or letter-number combination that indicated that the client needed to be referred to a facility  (e.g., “A.1”) ..… 1  “X” ………………. 2  Other …………… 3  Unsure ………… 4 | Any number or letter-number combination that indicated that the client needed to be referred to a facility  (e.g., “A.1”) ..… 1  “X” ………………. 2  Other …………… 3  Unsure ………… 4 | | Any number or letter-number combination that indicated that the client needed to be referred to a facility  (e.g., “A.1”) ..… 1  “X” ………………. 2  Other …………… 3  Unsure ………… 4 | Any number or letter-number combination that indicated that the client needed to be referred to a facility  (e.g., “A.1”) ..… 1  “X” ………………. 2  Other …………… 3  Unsure ………… 4 | Any number or letter-number combination that indicated that the client needed to be referred to a facility  (e.g., “A.1”) ..… 1  “X” ………………. 2  Other …………… 3  Unsure ………… 4 | |
| tHFA | EA12 | COLUMN 18. “Comptage mvt resp” (i.e., “Respiratory rate”) | Any number … 1  “X” ………………. 2  Other …………… 3  Unsure ………… 4 | Any number … 1  “X” ………………. 2  Other …………… 3  Unsure ………… 4 | | Any number … 1  “X” ………………. 2  Other …………… 3  Unsure ………… 4 | Any number … 1  “X” ………………. 2  Other …………… 3  Unsure ………… 4 | Any number … 1  “X” ………………. 2  Other …………… 3  Unsure ………… 4 | |
| tHFA | EA13 | COLUMN 19. “Tirage sous costal” (i.e., “chest indrawing”) | Check mark .… 1  “X” ………………. 2  Other …………… 3  Unsure ………… 4 | Check mark .… 1  “X” ………………. 2  Other …………… 3  Unsure ………… 4 | | Check mark .… 1  “X” ………………. 2  Other …………… 3  Unsure ………… 4 | Check mark .… 1  “X” ………………. 2  Other …………… 3  Unsure ………… 4 | Check mark .… 1  “X” ………………. 2  Other …………… 3  Unsure ………… 4 | |
| tHFA | EA14 | COLUMN 20. “Toux/Rhume”  (i.e., “Cough/Cold”) | Check mark .… 1  “X” ………………. 2  Other …………… 3  Unsure ………… 4 | Check mark .… 1  “X” ………………. 2  Other …………… 3  Unsure ………… 4 | | Check mark .… 1  “X” ………………. 2  Other …………… 3  Unsure ………… 4 | Check mark .… 1  “X” ………………. 2  Other …………… 3  Unsure ………… 4 | Check mark .… 1  “X” ………………. 2  Other …………… 3  Unsure ………… 4 | |
| tHFA | EA15 | COLUMN 21. “Pneumonie”  (i.e., “Pneumonia”) | Check mark .… 1  “X” ………………. 2  Other …………… 3  Unsure ………… 4 | Check mark .… 1  “X” ………………. 2  Other …………… 3  Unsure ………… 4 | | Check mark .… 1  “X” ………………. 2  Other …………… 3  Unsure ………… 4 | Check mark .… 1  “X” ………………. 2  Other …………… 3  Unsure ………… 4 | Check mark .… 1  “X” ………………. 2  Other …………… 3  Unsure ………… 4 | |
| tHFA | EA16 | COLUMN 23. “TDR réalisé”  (i.e., “Malaria rapid diagnostic test (RDT) performed”) | Check mark .… 1  “X” ………………. 2  Other …………… 3  Unsure ………… 4 | Check mark .… 1  “X” ………………. 2  Other …………… 3  Unsure ………… 4 | | Check mark .… 1  “X” ………………. 2  Other …………… 3  Unsure ………… 4 | Check mark .… 1  “X” ………………. 2  Other …………… 3  Unsure ………… 4 | Check mark .… 1  “X” ………………. 2  Other …………… 3  Unsure ………… 4 | |
| tHFA | EA17 | COLUMN 24. “Paludism”  (i.e., “Malaria”) | Check mark .… 1  “X” ………………. 2  Other …………… 3  Unsure ………… 4 | Check mark .… 1  “X” ………………. 2  Other …………… 3  Unsure ………… 4 | | Check mark .… 1  “X” ………………. 2  Other …………… 3  Unsure ………… 4 | Check mark .… 1  “X” ………………. 2  Other …………… 3  Unsure ………… 4 | Check mark .… 1  “X” ………………. 2  Other …………… 3  Unsure ………… 4 | |
| tHFA | EA18 | COLUMN 25. “Fièvre”  (i.e., “non-malaria febrile illness”) | Check mark .… 1  “X” ………………. 2  Other …………… 3  Unsure ………… 4 | Check mark .… 1  “X” ………………. 2  Other …………… 3  Unsure ………… 4 | | Check mark .… 1  “X” ………………. 2  Other …………… 3  Unsure ………… 4 | Check mark .… 1  “X” ………………. 2  Other …………… 3  Unsure ………… 4 | Check mark .… 1  “X” ………………. 2  Other …………… 3  Unsure ………… 4 | |
| tHFA | EA19 | COLUMN 26. “TDR non disponible. Cas suspect de paludisme”  (i.e., “RDT not available. Suspected malaria case”) | Check mark .… 1  “X” ………………. 2  Other …………… 3  Unsure ………… 4 | Check mark .… 1  “X” ………………. 2  Other …………… 3  Unsure ………… 4 | | Check mark .… 1  “X” ………………. 2  Other …………… 3  Unsure ………… 4 | Check mark .… 1  “X” ………………. 2  Other …………… 3  Unsure ………… 4 | Check mark .… 1  “X” ………………. 2  Other …………… 3  Unsure ………… 4 | |
|  |  | FOR QUESTIONS EA20 TO EA24, ASK THE RECO SITE CHW IF THE CLIENT HAD BEEN GIVEN THE MEDICINE INDICATED IN THE QUESTION (WHICH CORRESPONDS TO RESPONSE CHOICE “1”). | | | | | |  | |
| tHFA | EA20 | COLUMN 31. “Amoxicilline”  (i.e., “Amoxicillin”)  ASK THE RECO SITE CHW IF THE CLIENT HAD BEEN GIVEN THE MEDICINE (I.E., RESPONSE “1”). | Any number, the letter “R”, or  word indicating that the client was given the medicine …...… 1  “X” ………………. 2  Other …………… 3  Unsure ………… 4 | Any number, the letter “R”, or  word indicating that the client was given the medicine …...… 1  “X” ………………. 2  Other …………… 3  Unsure ………… 4 | | Any number, the letter “R”, or  word indicating that the client was given the medicine …...… 1  “X” ………………. 2  Other …………… 3  Unsure ………… 4 | Any number, the letter “R”, or  word indicating that the client was given the medicine …...… 1  “X” ………………. 2  Other …………… 3  Unsure ………… 4 | Any number, the letter “R”, or  word indicating that the client was given the medicine …...… 1  “X” ………………. 2  Other …………… 3  Unsure ………… 4 | |
| tHFA | EA21 | COLUMN 36. "Art-AQ nouriss. 25/67,5mg" (i.e., “Artemether-Amodiaquine 25/67.5 mg for young children”)  ASK THE RECO SITE CHW IF THE CLIENT HAD BEEN GIVEN THE MEDICINE (I.E., RESPONSE “1”). | Any number, the letter “R”, or  word indicating that the client was given the medicine …...… 1  “X” ………………. 2  Other …………… 3  Unsure ………… 4 | Any number, the letter “R”, or  word indicating that the client was given the medicine …...… 1  “X” ………………. 2  Other …………… 3  Unsure ………… 4 | | Any number, the letter “R”, or  word indicating that the client was given the medicine …...… 1  “X” ………………. 2  Other …………… 3  Unsure ………… 4 | Any number, the letter “R”, or  word indicating that the client was given the medicine …...… 1  “X” ………………. 2  Other …………… 3  Unsure ………… 4 | Any number, the letter “R”, or  word indicating that the client was given the medicine …...… 1  “X” ………………. 2  Other …………… 3  Unsure ………… 4 | |
| tHFA | EA22 | COLUMN 37. "Art-AQ enft. 50/135mg" (i.e., “Artemether-Amodiaquine 50/135 mg for children”)  ASK THE RECO SITE CHW IF THE CLIENT HAD BEEN GIVEN THE MEDICINE (I.E., RESPONSE “1”). | Any number, the letter “R”, or  word indicating that the client was given the medicine …...… 1  “X” ………………. 2  Other …………… 3  Unsure ………… 4 | Any number, the letter “R”, or  word indicating that the client was given the medicine …...… 1  “X” ………………. 2  Other …………… 3  Unsure ………… 4 | | Any number, the letter “R”, or  word indicating that the client was given the medicine …...… 1  “X” ………………. 2  Other …………… 3  Unsure ………… 4 | Any number, the letter “R”, or  word indicating that the client was given the medicine …...… 1  “X” ………………. 2  Other …………… 3  Unsure ………… 4 | Any number, the letter “R”, or  word indicating that the client was given the medicine …...… 1  “X” ………………. 2  Other …………… 3  Unsure ………… 4 | |
| tHFA | EA23 | COLUMN 38. "Art-AQ 6 ans et adlt 100/270mg" (i.e., “Artemether-Amodiaquine 100/270 mg for children aged 6 years and older and adults”)  ASK THE RECO SITE CHW IF THE CLIENT HAD BEEN GIVEN THE MEDICINE (I.E., RESPONSE “1”). | Any number, the letter “R”, or  word indicating that the client was given the medicine …...… 1  “X” ………………. 2  Other …………… 3  Unsure ………… 4 | Any number, the letter “R”, or  word indicating that the client was given the medicine …...… 1  “X” ………………. 2  Other …………… 3  Unsure ………… 4 | | Any number, the letter “R”, or  word indicating that the client was given the medicine …...… 1  “X” ………………. 2  Other …………… 3  Unsure ………… 4 | Any number, the letter “R”, or  word indicating that the client was given the medicine …...… 1  “X” ………………. 2  Other …………… 3  Unsure ………… 4 | Any number, the letter “R”, or  word indicating that the client was given the medicine …...… 1  “X” ………………. 2  Other …………… 3  Unsure ………… 4 | |
| tHFA | EA24 | COLUMN 39. “Artémether-Lumefantrine” (i.e., “Artemether-Lumefantrin”)  ASK THE RECO SITE CHW IF THE CLIENT HAD BEEN GIVEN THE MEDICINE (I.E., RESPONSE “1”). | Any number, the letter “R”, or  word indicating that the client was given the medicine …...… 1  “X” ………………. 2  Other …………… 3  Unsure ………… 4 | Any number, the letter “R”, or  word indicating that the client was given the medicine …...… 1  “X” ………………. 2  Other …………… 3  Unsure ………… 4 | | Any number, the letter “R”, or  word indicating that the client was given the medicine …...… 1  “X” ………………. 2  Other …………… 3  Unsure ………… 4 | Any number, the letter “R”, or  word indicating that the client was given the medicine …...… 1  “X” ………………. 2  Other …………… 3  Unsure ………… 4 | Any number, the letter “R”, or  word indicating that the client was given the medicine …...… 1  “X” ………………. 2  Other …………… 3  Unsure ………… 4 | |
| tHFA | EA25 | COLUMN 66. "OBSERVATIONS.  O = orienté, OR = Orientation recours, G = Guerris” etc.  Was the response in column 66 an “O” or “Orienté”? (i.e., CHW referred the client to a facility) | YES ............. 1  NO ............. 2  UNSURE ….. 8 | YES ............. 1  NO ............. 2  UNSURE ….. 8 | | YES ............. 1  NO ............. 2  UNSURE ….. 8 | YES ............. 1  NO ............. 2  UNSURE ….. 8 | YES ............. 1  NO ............. 2  UNSURE ….. 8 | |
|  |  |  | | |  | | | |  |
|  |  | THANK THE RECO SITE CHW. PERFORM THE RECORD REVIEW WITH THE NEXT RECO SITE CHW, AND CONTINUE UNTIL ALL CONSENTED RECO SITE CHWS AT THE FACILITY HAVE BEEN SEEN. | | | | | | |  |
|  |  |  | | |  | | | |  |
|  |  | **END OF T-HFA SECTION 5A** | | | | | | |  |

| **Mod** | **No.** | **QUESTION** | **RESPONSE** | | | | **SKIP** |
| --- | --- | --- | --- | --- | --- | --- | --- |
|  |  | **T-HFA SECTION 5B. RECORD REVIEW FOR “RELAIS COMMUNAUTAIRE PROMOTIONNEL” (RECO PL)**  **(FOR DRC ONLY)**  Questions in this section are used to measure GF indicator RSSH/PP HRH-4. | | | | |  |
|  |  |  |  | | | |  |
|  |  | **PERFORM THE FOLLOWING RECORD REVIEW FOR THE (UP TO) 5 RECO PL CHWS.** | | | | |  |
| tHFA | EB01 | FIND SOMEONE AT THE FACILITY WHO IS KNOWLEDGEABLE ABOUT THE TYPES OF CHWS THAT ARE ASSOCIATED WITH THE FACILITY. ASK THIS QUESTION:  ARE THERE RELAIS COMMUNAUTAIRE PROMOTIONNEL ASSOCIATED WITH THIS FACILITY? | YES .................................................................. 1  NO .................................................................. 2  DON’T KNOW OR UNSURE ………………………….. 8 | | | | 🡺 END  🡺 END |
| tHFA | EB02 | ASK TO BE SHOWN THE RECO PL CHW MONTHLY REPORTING FORMS FROM THE PAST 3 COMPLETED MONTHS AND RESPOND TO THE FOLLOWING QUESTION.  HOW MANY RECO PL CHWS HAVE AT LEAST ONE MONTHLY REPORTING FORM FROM THE PAST 3 COMPLETED MONTHS AT THE FACILITY THAT YOU ARE ABLE TO DIRECTLY OBSERVE? IF THERE ARE FORMS FROM ONLY 1 OR 2 MONTHS, THAT IS ACCEPTABLE (USE WHICHEVER FORMS ARE AVAILABLE). | …………………………… NUMBER OF RECO PL CHWS | | | | 🡺 IF ZERO RECO PL CHWS, THEN END |
|  |  | IF THERE ARE MONTHLY REPORTING FORMS FROM MORE THAN 5 RECO PL CHWS, THEN USE SAMPLING METHODS DESCRIBED ABOVE TO RANDOMLY SELECT 5 RECO PL CHWS.  ***Note on the questionnaire structure. This section of the questionnaire (below) shows one set of questions for each CHW record review. When the t-HFA questionnaire (either paper or electronic version) is created for field use, these questions will be repeated five times, for the goal of collecting data from the records of five CHWs per facility.*** | | | | |  |
|  |  |  | | FORM FROM LAST MONTH | FORM FROM 2 MONTHS AGO | FORM FROM 3 MONTHS AGO | |
| tHFA | EB03 | RECORD THE NUMBER OF HOUSEHOLDS VISITED DURING THE MONTH (“VAD/ Visite des menages réalisées”). IN THE 2024 FORM, THIS IS DATA ELEMENT NO. 19 ON PAGE 3. IN THE 2023 FORM, THIS IS DATA ELEMENT NO. 15 ON PAGE 2. | | …………… NUMBER OF HOUSEHOLDS VISITED | …………… NUMBER OF HOUSEHOLDS VISITED | …………… NUMBER OF HOUSEHOLDS VISITED | |
| tHFA | EB04 | RECORD THE NUMBER OF HOUSEHOLDS WITH A LONG-LASTING INSECTICIDE-TREATED NET ("Ménages qui disposent la MIILD"). IN THE 2024 FORM, THIS IS DATA ELEMENT 29 ON PAGE 3. IN THE 2023 FORM, THIS IS DATA ELEMENT 20 ON PAGE 2. | | …………… NUMBER OF HOUSEHOLDS | …………… NUMBER OF HOUSEHOLDS | …………… NUMBER OF HOUSEHOLDS | |
| tHFA | EB05 | RECORD THE NUMBER OF HOUSEHOLDS WITH CHILDREN UNDER 5 YEARS OLD WHO SLEPT UNDER A LONG-LASTING INSECTICIDE-TREATED NET ("Ménages ayant des enfants de moins de 5 ans qui dorment sous la MIILD"). IN THE 2024 FORM, THIS IS DATA ELEMENT 30 ON PAGE 3. IN THE 2023 FORM, THIS IS DATA ELEMENT 21 ON PAGE 2. | | …………… NUMBER OF HOUSEHOLDS | …………… NUMBER OF HOUSEHOLDS | …………… NUMBER OF HOUSEHOLDS | |
| tHFA | EB06 | RECORD THE NUMBER OF WOMEN WHO DELIVER AT HOME ("Femmes ayant accouché à domicile"). IN THE 2024 FORM, THIS IS DATA ELEMENT 58 ON PAGE 5. IN THE 2023 FORM, THIS IS DATA ELEMENT 36 ON PAGE 2. | | …………… NUMBER OF WOMEN | …………… NUMBER OF WOMEN | …………… NUMBER OF WOMEN | |
| tHFA | EB07 | RECORD THE NUMBER OF WOMEN WHO DELIVER AT HOME THAT WERE REFERRED TO A HEALTH CENTER ("Femmes ayant accouché à domicile et orientées vers le CS"). IN THE 2024 FORM, THIS IS DATA ELEMENT 59 ON PAGE 5. IN THE 2023 FORM, THIS IS DATA ELEMENT 37 ON PAGE 2. | | …………… NUMBER OF WOMEN | …………… NUMBER OF WOMEN | …………… NUMBER OF WOMEN | |
| tHFA | EB08 | RECORD THE NUMBER OF 0–6 MONTHS OLD ("Enfants de 0 à 6 mois"). IN THE 2024 FORM, THIS IS DATA ELEMENT 4 ON PAGE 2. IN THE 2023 FORM, THIS IS DATA ELEMENT 3 ON PAGE 2. | | …………… NUMBER OF INFANTS | …………… NUMBER OF INFANTS | …………… NUMBER OF INFANTS | |
| tHFA | EB09 | RECORD THE NUMBER OF 0–6 MONTHS OLD THAT ARE EXCLUSIVELY BREASTFED ("Enfants de 0 à 6 mois allaités exclusivement au sein"). IN THE 2024 FORM, THIS IS DATA ELEMENT 36 ON PAGE 3. IN THE 2023 FORM, THIS IS DATA ELEMENT 27 ON PAGE 2. | | …………… NUMBER OF INFANTS | …………… NUMBER OF INFANTS | …………… NUMBER OF INFANTS | |
|  |  |  |  | | | |  |
|  |  | **END OF T-HFA SECTION 5B** | | | | |  |

| **Mod** | **No.** | **QUESTION** | | | | | **RESPONSE** | | | | | **SKIP** |
| --- | --- | --- | --- | --- | --- | --- | --- | --- | --- | --- | --- | --- |
|  |  | **T-HFA SECTION 6. RECORD REVIEW OF ANTENATAL CARE (ANC) CLIENTS**  Source: WHO (2022) HHFA Module 3: Quality of care.  Questions in this section are used to measure GF indicators KPI S1 and KPI S3. | | | | | | | | | |  |
| Q | 13000 | Are antenatal care services offered in this facility? | | | | | YES .................................................................................................................... 1  NO ..................................................................................................................... 2 | | | | | ➔ End of section 6 |
| Q | 13001 | **ELIGIBILITY CRITERIA:** CLIENT ATTENDED ANC AND WAS AT LEAST 32 OR MORE WEEKS PREGNANT DURING MOST RECENT VISIT **OR** COUNTRY SPECIFIC ELIGIBILITY CRITERIA NOT BASED ON GESTATIONAL AGE [COUNTRY ADAPT ELIGIBILITY CRITERIA]  **SAMPLE SELECTION:**  THE RECORD REVIEW REQUIRES TWO STEPS: (1) IDENTIFYING A SAMPLE OF ELIGIBLE CLIENT RECORDS, AND (2) OBTAINING AND ASSESSING THE ELIGIBLE RECORDS.  ***1. IDENTIFYING A SAMPLE OF ELIGIBLE CLIENT RECORDS:***  THIS STEP REQUIRES ACCESS TO A LIST OF CLIENTS THAT HAVE USED THE FACILITY FOR THE CONDITION ASSESSED IN THE RECORD REVIEW. THE LIST MAY BE FOUND IN A REGISTER, APPOINTMENT BOOK, APPOINTMENT CARD BOX, DATABASE OR OTHER LIST. THIS LIST WILL BE THE SOURCE DOCUMENT FOR THE SAMPLE.  ASK TO BE GIVEN THE SOURCE DOCUMENT/REGISTER AND IDENTIFY THE MOST RECENT COMPLETE MONTH WHERE CLIENTS CAN BE IDENTIFIED. COUNTING BACKWARDS FROM THE END OF THE MOST RECENT COMPLETE MONTH, IDENTIFY 10 CLIENTS WHO MEET THE ELIGIBILITY CRITERIA FOR THIS RECORD REVIEW. CONTINUE COUNTING ALL ELIGIBLE CLIENTS IN EACH PRECEDING MONTH TO A MAXIMUM OF 6 FULL MONTHS, IF NEEDED, TO IDENTIFY 10 CLIENTS MEETING THE ELIGIBILITY CRITERIA. DO NOT EXCEED SIX MONTHS EVEN IF 10 CLIENTS HAVE NOT BEEN IDENTIFIED. COMPLETE A CLIENT SAMPLING LIST (SEE ANNEX 3 “RECORD REVIEW SAMPLING TOOL”) BY RECORDING IDENTIFYING INFORMATION FOR THE ELIGIBLE CLIENTS. THE GOAL IS TO IDENTIFY 10 ELIGIBLE CLIENTS.  **IF 10 ELIGIBLE CLIENTS HAVE BEEN IDENTIFIED:** THE COMPLETED CLIENT SAMPLING LIST HAS A SAMPLING NUMBER BETWEEN 1 AND 10 FOR EACH CLIENT. TO SELECT THE 5 CLIENTS WHOSE INFORMATION WILL BE ENTERED INTO THE T-HFA QUESTIONNAIRE, TOSS A COIN. ONE SIDE WILL MEAN YOU SELECT CLIENTS WITH SAMPLING NUMBERS 1, 3, 5, 7, AND 9; THE OTHER SIDE WILL MEAN YOU SELECT CLIENTS 2, 4, 6, 8, AND 10.  **IF FEWER THAN 10 ELIGIBLE CLIENTS HAVE BEEN IDENTIFIED:** TO SELECT THE 5 CLIENTS WHOSE INFORMATION WILL BE ENTERED INTO THE T-HFA QUESTIONNAIRE, FOLLOW THE INSTRUCTIONS IN THE RECORD REVIEW SAMPLING TOOL (ANNEX 3).  ***2. OBTAINING AND ASSESSING THE ELIGIBLE RECORDS:***  USING THE IDENTIFYING INFORMATION FOR EACH OF THE 5 CLIENTS SELECTED FOR THE SAMPLE, ASK TO SEE THE INDIVIDUAL RECORD FOR THESE CLIENTS. A “RECORD” REFERS TO A DOCUMENT THAT CONTAINS INFORMATION ON INDIVIDUAL CLIENT CARE. THIS INFORMATION MAY BE CONTAINED IN A REGISTER, INDIVIDUAL CLIENT CARD/FILE/CHART, ETC., BUT MUST BE RECORDED AT THE CLIENT LEVEL AND PROVIDE SPECIFICS ON THE CONTENT OF CARE DELIVERED TO THE INDIVIDUAL. IN SOME CASES, THE INFORMATION WILL BE AVAILABLE IN A REGISTER AND INDIVIDUAL CARDS/CHARTS WILL NOT NEED TO BE LOCATED.  IF THE INDIVIDUAL CLIENT CARD/FILE/CHART FOR A SELECTED INDIVIDUAL CANNOT BE LOCATED, THE INDIVIDUAL MAY BE REPLACED WITH THE NEXT ELIGIBLE PERSON ON THE CLIENT SAMPLING LIST WHO HAS NOT ALREADY BEEN SELECTED. FOR EXAMPLE, CONSIDER THE SCENARIO IN WHICH YOU HAVE IDENTIFIED 10 ELIGIBLE CLIENTS, AND YOU HAVE FLIPPED A COIN AND SELECTED CLIENTS 1, 3, 5, 7, AND 9. IF THE RECORD FOR CLIENT 3 CANNOT BE LOCATED, THEN REPLACE CLIENT 3 WITH CLIENT 4. REMEMBER THAT THE GOAL IS TO SELECT 5 CLIENTS AND ENTER THEIR INFORMATION INTO THE T-HFA QUESTIONNAIRE.  [COUNTRY ADAPT STARTING MONTH AND DIRECTION OF COUNTING ACCORDING TO REGISTER FORMAT IN USE] | | | | | | | | | |  |
| Q | 13001  _01 | NUMBER OF DIFFERENT MONTHS FROM WHICH THE SAMPLE WAS SELECTED | | | | | ___ NUMBER OF MONTHS | | | | |  |
| Q | 13001  _02 | NUMBER OF ELIGIBLE CLIENTS IDENTIFIED | | | | | ___ ___ NUMBER OF ELIGIBLE CLIENTS    NO ELIGIBLE CLIENTS IDENTIFIED ............................................... 00 | | | | | ➔ 13012 |
| tHFA | 13001 _02b | NUMBER OF ELIGIBLE CLIENTS SAMPLED | | | | | ___ ___ NUMBER OF ELIGIBLE CLIENTS SAMPLED | | | | |  |
| Q | 13001  _03 | NUMBER OF ORIGINALLY SELECTED SAMPLE CLIENTS REPLACED DUE TO MISSING INDIVIDUAL CLIENT RECORDS  **NOTE: RECORD AFTER COMPLETION OF RECORD REVIEW** | | | | | ___ NUMBER REPLACED  NONE ............................................................................................ 0 | | | | |  |
| Q | 13001  _04 | SOURCE DATA FOR SAMPLE SELECTION  IF MORE THAN ONE SOURCE IS USED TO IDENTIFY THE SAMPLE, CIRCLE (ENTER RESPONSES FOR) ALL THAT APPLY  **NOTE:** SOURCE DOCUMENT REFERS TO REGISTERS AND RECORDS THAT CONTAIN INFORMATION ON CLIENTS WHO HAVE RECEIVED ANTENATAL CARE SERVICES AND CAN SERVE AS A SAMPLING LIST FOR THE RECORD REVIEW. | | | | | ANTENATAL CARE REGISTER .............................................................................. A  INDIVIDUAL CLIENT ANC/MNCH/PMTCT CARDS/CHARTS/RECORDS ................ B  PMTCT REGISTER ................................................................................................C  OUTPATIENT DEPARTMENT (OPD) REGISTER .................................................... D  LABORATORY REGISTER ..................................................................................... E  PHARMACY REGISTER ........................................................................................ F  LABOUR AND DELIVERY REGISTER ..................................................................... G  ITN REGISTER ..................................................................................................... H  OTHER (13001_05A) ______________________________________________ X  (SPECIFY) | | | | |  |
|  |  |  | | | | |  | | | | |  |
| Q | 13007 | **IPT SERVICES FOR MALARIA DOCUMENTED AT ANY VISIT** | | | | | | | | | | |
|  |  |  | | CLIENT  1 | | CLIENT  2 | | | CLIENT  3 | CLIENT  4 | CLIENT  5 | |
| tHFA | RR_ANC_Begin | ENUMERATOR, DO YOU HAVE ANC CLIENT RECORDS WITH YOU FOR THE RECORD REVIEW? | | YES ……………………… 1  NO ……………………… 2 | | YES ……………………… 1  NO ……………………… 2 | | | YES ……………………… 1  NO ……………………… 2 | YES ……………………… 1  NO ……………………… 2 | YES ……………………… 1  NO ……………………… 2 | |
| tHFA | 13000_Client_ID | Client ID from the record register | | ………………………………… | | ………………………………… | | | ………………………………… | ………………………………… | ………………………………… | |
| tHFA | 13000_Client_Name | Client Name from the record register | | ………………………………… | | ………………………………… | | | ………………………………… | ………………………………… | ………………………………… | |
| Q | 13007 _03 | Is there documentation at any visit that the client received 1 dose of IPT? | | YES …………….……..…. 1  NO ……………………….. 2 | | YES …………….……..…. 1  NO ……………………….. 2 | | | YES …………….……..…. 1  NO ……………………….. 2 | YES …………….……..…. 1  NO ……………………….. 2 | YES …………….……..…. 1  NO ……………………….. 2 | |
|  |  |  | |  | |  | | |  |  |  | |
| Q |  | **HIV SERVICES** | | | | | | | | | | |
| Q | 13009 | Are any of the below recorded for any visit? | | | | | | | | | | |
| Q | 13009  _01 | Was the client on ART prior to attending ANC? | | YES …………….……..…. 1  NO ……………………….. 2 | | YES …………….……..…. 1  NO ……………………….. 2 | | | YES …………….……..…. 1  NO ……………………….. 2 | YES …………….……..…. 1  NO ……………………….. 2 | YES …………….……..…. 1  NO ……………………….. 2 | |
| tHFA | F003 | Is there documentation at any visit that the client received an HIV test during ANC? | | YES …………….……..…. 1  NO ……………………….. 2 | | YES …………….……..…. 1  NO ……………………….. 2 | | | YES …………….……..…. 1  NO ……………………….. 2 | YES …………….……..…. 1  NO ……………………….. 2 | YES …………….……..…. 1  NO ……………………….. 2 | |
| tHFA | F001 | Is there documentation that the client is HIV positive (diagnosed either during or prior to her pregnancy)? | | YES …………….……..…. 1  NO ……………………….. 2 | | YES …………….……..…. 1  NO ……………………….. 2 | | | YES …………….……..…. 1  NO ……………………….. 2 | YES …………….……..…. 1  NO ……………………….. 2 | YES …………….……..…. 1  NO ……………………….. 2 | |
| tHFA | F004 | For HIV-positive women, is there documentation that the woman is on ART during pregnancy or breastfeeding? | | YES …………….……..…. 1  NO ……………………….. 2 | | YES …………….……..…. 1  NO ……………………….. 2 | | | YES …………….……..…. 1  NO ……………………….. 2 | YES …………….……..…. 1  NO ……………………….. 2 | YES …………….……..…. 1  NO ……………………….. 2 | |
|  |  |  | |  | |  | | |  |  |  | |
| tHFA |  | **TB SERVICES (SCREENING) DOCUMENTED AT ANY ANC VISIT** | | | | | | | | | | |
| TBS | F002 | Is there documentation at any ANC visit that the client was screened for TB with questions about 4 symptoms (i.e., documentation of the **presence or absence** of: cough, fever, night sweats, and weight loss)? Information on the presence or absence of the 4 symptoms must be asked at the same visit. If the number of symptoms recorded varies by ANC visit for a given client, find the ANC visit with information on the greatest number of symptoms (present or absent) recorded. For example, if fever (marked as either present or absent) is the only one of the 4 symptoms recorded at the first ANC visit, but fever and cough (marked as either present or absent) is recorded at the second ANC visit, then enter the information for the second ANC visit. | | A) Is cough recorded as either present or absent?  YES …………….………… 1  NOT RECORDED …… 2 | | A) Is cough recorded as either present or absent?  YES …………….………… 1  NOT RECORDED …… 2 | | | A) Is cough recorded as either present or absent?  YES …………….…………. 1  NOT RECORDED ……. 2 | A) Is cough recorded as either present or absent?  YES …………….…………. 1  NOT RECORDED ……. 2 | A) Is cough recorded as either present or absent?  YES …………….………… 1  NOT RECORDED ……. 2 | |
|  |  |  |  | B) Is fever recorded as either present or absent?  YES …………….………… 1  NOT RECORDED …… 2 | | B) Is fever recorded as either present or absent?  YES …………….………… 1  NOT RECORDED …… 2 | | | B) Is fever recorded as either present or absent?  YES …………….…………. 1  NOT RECORDED ……. 2 | B) Is fever recorded as either present or absent?  YES …………….…………. 1  NOT RECORDED ……. 2 | B) Is fever recorded as either present or absent?  YES …………….………… 1  NOT RECORDED ……. 2 | |
|  |  |  |  | C) Are night sweats recorded as either present or absent?  YES …………….………… 1  NOT RECORDED …… 2 | | C) Are night sweats recorded as either present or absent?  YES …………….………… 1  NOT RECORDED …… 2 | | | C) Are night sweats recorded as either present or absent?  YES …………….…………. 1  NOT RECORDED ……. 2 | C) Are night sweats recorded as either present or absent?  YES …………….…………. 1  NOT RECORDED ……. 2 | C) Are night sweats recorded as either present or absent?  YES …………….………… 1  NOT RECORDED ……. 2 | |
|  |  |  |  | D) Is weight loss recorded as either present or absent?  YES …………….………… 1  NOT RECORDED …… 2 | | D) Is weight loss recorded as either present or absent?  YES …………….………… 1  NOT RECORDED …… 2 | | | D) Is weight loss recorded as either present or absent?  YES …………….…………. 1  NOT RECORDED ……. 2 | D) Is weight loss recorded as either present or absent?  YES …………….…………. 1  NOT RECORDED ……. 2 | D) Is weight loss recorded as either present or absent?  YES …………….………… 1  NOT RECORDED ……. 2 | |
| Q | 13004 | **GENERAL ANC SERVICES. PLEASE ANSWER THE FOLLOWING QUESTIONS FOR THE MOST RECENT ANC VISIT** | | | | | | | | | | |
| Q | 13004  _01 | What was the recorded gestational age (in weeks) at the most recent ANC visit? | ___ ___ WEEKS  NOT RECORDED .... 98 | | ___ ___ WEEKS  NOT RECORDED .... 98 | | | ___ ___ WEEKS  NOT RECORDED .... 98 | | ___ ___ WEEKS  NOT RECORDED .... 98 | ___ ___ WEEKS  NOT RECORDED .... 98 | |
| Q | 13004  _07 | Which ANC visit (number) does the most recent visit represent? | ___ ___ VISITS  NOT RECORDED .... 98 | | ___ ___ VISITS  NOT RECORDED .... 98 | | | ___ ___ VISITS  NOT RECORDED .... 98 | | ___ ___ VISITS  NOT RECORDED .... 98 | ___ ___ VISITS  NOT RECORDED .... 98 | |
| Q | 13004  _08 | Is the client’s blood pressure documented for the most recent visit? | YES …………….………… 1  NO ……………………….. 2 | | YES …………….………… 1  NO ……………………….. 2 | | | YES …………….…………. 1  NO ……………………….. 2 | | YES …………….…………. 1  NO ……………………….. 2 | YES …………….………… 1  NO ……………………….. 2 | |
| Q | 13004  _10 | Is it documented that the client was provided or prescribed iron and folic acid during the most recent visit? | YES …………….………… 1  NO ……………………….. 2 | | YES …………….………… 1  NO ……………………….. 2 | | | YES …………….…………. 1  NO ……………………….. 2 | | YES …………….…………. 1  NO ……………………….. 2 | YES …………….………… 1  NO ……………………….. 2 | |
| Q | 13004  _11 | Is there documentation that the client received counseling on pregnancy danger signs at the most recent visit? | YES …………….………… 1  NO ……………………….. 2 | | YES …………….………… 1  NO ……………………….. 2 | | | YES …………….…………. 1  NO ……………………….. 2 | | YES …………….…………. 1  NO ……………………….. 2 | YES …………….………… 1  NO ……………………….. 2 | |
| tHFA |  | **GENERAL ANC SERVICES. PLEASE ANSWER THE FOLLOWING QUESTIONS FOR ANY ANC VISIT** | | | | | | | | | | |
| Q | 13005  _01 | Is a syphilis blood test result documented at any ANC visit? | YES …………….………… 1  NO ……………………….. 2 | | YES …………….………… 1  NO ……………………….. 2 | | | YES …………….…………. 1  NO ……………………….. 2 | | YES …………….…………. 1  NO ……………………….. 2 | YES …………….………… 1  NO ……………………….. 2 | |
| Q | 13005  _02 | Is there documentation that the client received any medicine for the treatment of intestinal worms (e.g., albendazole, mebendazole)? | YES …………….………… 1  NO ……………………….. 2 | | YES …………….………… 1  NO ……………………….. 2 | | | YES …………….…………. 1  NO ……………………….. 2 | | YES …………….…………. 1  NO ……………………….. 2 | YES …………….………… 1  NO ……………………….. 2 | |
| Q | 13005  _05 | Is any hemoglobin or hematocrit result documented for any ANC visit during this pregnancy? | YES …………….………… 1  NO ……………………….. 2 | | YES …………….………… 1  NO ……………………….. 2 | | | YES …………….…………. 1  NO ……………………….. 2 | | YES …………….…………. 1  NO ……………………….. 2 | YES …………….………… 1  NO ……………………….. 2 | |
| Q | 13012 | ANY ISSUES OR EXPLANATIONS TO DOCUMENT RELATED TO THE RECORD REVIEW | | | YES ...................................................................................... 1  (13012A) _____________________________________________________________  (IF YES, SPECIFY)  NO ....................................................................................... 2 | | | | | | |  |
|  |  | **END OF T-HFA SECTION 6** | | | | | | | | | |  |

| **Mod** | **No.** | **QUESTION** | | | **RESPONSE** | | | | | **SKIP** |
| --- | --- | --- | --- | --- | --- | --- | --- | --- | --- | --- |
|  |  | **T-HFA SECTION 7. ANTIRETROVIRAL THERAPY (ART) RECORD REVIEW**  Source: WHO (2022) HHFA Module 3: Quality of care. Questions in this section are used to measure GF indicators KPI S1. | | | | | | | |  |
| Q | 13400 | Does this facility offer ART services? | | | YES .................................................................................................................... 1  NO ..................................................................................................................... 2 | | | | | ➔ End of section 7 |
| Q | 13401 | **ELIGIBILITY CRITERIA:**  CURRENT ART CLIENT (15 OR MORE YEARS OF AGE) ON NATIONAL FIRST LINE ART REGIMEN THAT HAS COMPLETED AT LEAST 6 MONTHS OF ART  [COUNTRY ADAPT ELIGIBILITY CRITERIA. If there is a desire to conduct the ART record review for additional age groups (i.e., under 5 years, 5-14 years), please duplicate the ART record review and implement the ART record review procedures separately for each age group of interest.]    **SAMPLE SELECTION:**  THE RECORD REVIEW REQUIRES TWO STEPS: (1) IDENTIFYING A SAMPLE OF ELIGIBLE CLIENT RECORDS, AND (2) OBTAINING AND ASSESSING THE ELIGIBLE RECORDS.    ***1. IDENTIFYING A SAMPLE OF ELIGIBLE CLIENT RECORDS:***  THIS STEP REQUIRES ACCESS TO A LIST OF CLIENTS THAT HAVE USED THE FACILITY FOR THE CONDITION ASSESSED IN THE RECORD REVIEW. THE LIST MAY BE FOUND IN A REGISTER, APPOINTMENT BOOK, APPOINTMENT CARD BOX, DATABASE OR OTHER LIST. THIS LIST WILL BE THE SOURCE DOCUMENT FOR THE SAMPLE.    ASK TO BE GIVEN THE SOURCE DOCUMENT/REGISTER AND IDENTIFY THE CURRENT MONTH IN THE PRECEEDING YEAR WHERE CLIENTS CAN BE IDENTIFIED. COUNTING FORWARDS FROM THE BEGINNING OF THE CURRENT MONTH IN THE PRECEEDING YEAR, IDENTIFY 10 CLIENTS WHO MEET THE ELIGIBILITY CRITERIA FOR THIS RECORD REVIEW. CONTINUE COUNTING ALL ELIGIBLE CLIENTS TO A MAXIMUM OF 6 FULL MONTHS, IF NEEDED, TO IDENTIFY 10 CLIENTS MEETING THE ELIGIBILITY CRITERIA. DO NOT EXCEED SIX MONTHS EVEN IF 10 CLIENTS HAVE NOT BEEN IDENTIFIED. COMPLETE A CLIENT SAMPLING LIST (SEE ANNEX 3 “RECORD REVIEW SAMPLING TOOL”) BY RECORDING IDENTIFYING INFORMATION FOR THE ELIGIBLE CLIENTS. THE GOAL IS TO IDENTIFY 10 ELIGIBLE CLIENTS.  **IF 10 ELIGIBLE CLIENTS HAVE BEEN IDENTIFIED:** THE COMPLETED CLIENT SAMPLING LIST HAS A SAMPLING NUMBER BETWEEN 1 AND 10 FOR EACH CLIENT. TO SELECT THE 5 CLIENTS WHOSE INFORMATION WILL BE ENTERED INTO THE T-HFA QUESTIONNAIRE, TOSS A COIN. ONE SIDE WILL MEAN YOU SELECT CLIENTS WITH SAMPLING NUMBERS 1, 3, 5, 7, AND 9; THE OTHER SIDE WILL MEAN YOU SELECT CLIENTS 2, 4, 6, 8, AND 10.  **IF FEWER THAN 10 ELIGIBLE CLIENTS HAVE BEEN IDENTIFIED:** TO SELECT THE 5 CLIENTS WHOSE INFORMATION WILL BE ENTERED INTO THE T-HFA QUESTIONNAIRE, FOLLOW THE INSTRUCTIONS IN THE RECORD REVIEW SAMPLING TOOL (ANNEX 3).  ***2. OBTAINING AND ASSESSING THE ELIGIBLE RECORDS:***  USING THE IDENTIFYING INFORMATION FOR EACH OF THE 5 CLIENTS SELECTED FOR THE SAMPLE, ASK TO SEE THE INDIVIDUAL RECORD FOR THESE CLIENTS. A “RECORD” REFERS TO A DOCUMENT THAT CONTAINS INFORMATION ON INDIVIDUAL CLIENT CARE. THIS INFORMATION MAY BE CONTAINED IN A REGISTER, INDIVIDUAL CLIENT CARD/FILE/CHART, ETC., BUT MUST BE RECORDED AT THE CLIENT LEVEL AND PROVIDE SPECIFICS ON THE CONTENT OF CARE DELIVERED TO THE INDIVIDUAL. IN SOME CASES, THE INFORMATION WILL BE AVAILABLE IN A REGISTER AND INDIVIDUAL CARDS/CHARTS WILL NOT NEED TO BE LOCATED.  IF THE INDIVIDUAL CLIENT CARD/FILE/CHART FOR A SELECTED INDIVIDUAL CANNOT BE LOCATED, THE INDIVIDUAL MAY BE REPLACED WITH THE NEXT ELIGIBLE PERSON ON THE CLIENT SAMPLING LIST WHO HAS NOT ALREADY BEEN SELECTED. FOR EXAMPLE, CONSIDER THE SCENARIO IN WHICH YOU HAVE IDENTIFIED 10 ELIGIBLE CLIENTS, AND YOU HAVE FLIPPED A COIN AND SELECTED CLIENTS 1, 3, 5, 7, AND 9. IF THE RECORD FOR CLIENT 3 CANNOT BE LOCATED, THEN REPLACE CLIENT 3 WITH CLIENT 4. REMEMBER THAT THE GOAL IS TO SELECT 5 CLIENTS AND ENTER THEIR INFORMATION INTO THE T-HFA QUESTIONNAIRE.  [COUNTRY ADAPT STARTING MONTH AND DIRECTION OF COUNTING ACCORDING TO REGISTER FORMAT IN USE] | | | | | | | |  |
| Q | 13401 _01 | NUMBER OF DIFFERENT MONTHS FROM WHICH THE SAMPLE WAS SELECTED | | | ___ NUMBER OF MONTHS | | | | |  |
| Q | 13401 _02 | NUMBER OF ELIGIBLE CLIENTS IDENTIFIED | | | ___ ___ NUMBER OF ELIGIBLE CLIENTS    NO ELIGIBLE CLIENTS IDENTIFIED ............................................... 00 | | | | | ➔ 13411 |
| tHFA | 13401 _02b | NUMBER OF ELIGIBLE CLIENTS SAMPLED | | | ___ ___ NUMBER OF ELIGIBLE CLIENTS SAMPLED | | | | |  |
| Q | 13401 _03 | NUMBER OF ORIGINALLY SELECTED SAMPLE CLIENTS REPLACED DUE TO MISSING INDIVIDUAL CLIENT RECORDS  **NOTE: RECORD AFTER COMPLETION OF RECORD REVIEW** | | | ___ NUMBER REPLACED  NONE ............................................................................................ 0 | | | | |  |
| Q | 13401 _04 | SOURCE DATA FOR SAMPLE SELECTION  IF MORE THAN ONE SOURCE IS USED TO IDENTIFY THE SAMPLE, CIRCLE ALL THAT APPLY.  **NOTE:** SOURCE DOCUMENT REFERS TO REGISTERS AND RECORDS THAT CONTAIN INFORMATION ON CLIENTS WHO HAVE RECEIVED ART SERVICES AND CAN SERVE AS A SAMPLING LIST FOR THE RECORD REVIEW. | | | HIV TESTING REGISTER ....................................................................................... A  ART REGISTER ..................................................................................................... B  ART ENROLLMENT REGISTER .............................................................................. C  ART INITIATION REGISTER .................................................................................. D  ART TREATMENT REGISTER ................................................................................ E  INDIVIDUAL ART CLIENT CARDS/CHARTS/RECORDS ........................................... F  OTHER (13401_05A) _______________________________________________ X  (SPECIFY) | | | | |  |
| Q | 13408 | **CLIENT’S MOST RECENT CLINICAL VISIT (E.G., “LONG” VISIT—NOT SIMPLY TO PICK UP DRUGS)** | | | | | | | |  |
|  |  |  | CLIENT  1 | CLIENT  2 | | | CLIENT  3 | CLIENT  4 | CLIENT  5 | |
| tHFA | RR_ART_Begin | ENUMERATOR, DO YOU HAVE ART CLIENT RECORDS WITH YOU FOR THE RECORD REVIEW? | YES ……………………… 1  NO ……………………… 2 | YES ……………………… 1  NO ……………………… 2 | | | YES ……………………… 1  NO ……………………… 2 | YES ……………………… 1  NO ……………………… 2 | YES ……………………… 1  NO ……………………… 2 | |
| tHFA | 13400_Client_ID | Client ID from the record register | ………………………………… | ………………………………… | | | ………………………………… | ………………………………… | ………………………………… | |
| tHFA | 13400_Client_Name | Client Name from the record register | ………………………………… | ………………………………… | | | ………………………………… | ………………………………… | ………………………………… | |
| Q | 13408  _01 | Is there documentation that the client was assessed for cough at the most recent visit? | YES ……………………… 1  NO ……………………… 2 | YES ……………………… 1  NO ……………………… 2 | | | YES ……………………… 1  NO ……………………… 2 | YES ……………………… 1  NO ……………………… 2 | YES ……………………… 1  NO ……………………… 2 | |
| Q | 13408 _02 | Is there a measured temperature or a comment on history of fever status documented at the most recent visit? | YES ……………………… 1  NO ……………………… 2 | YES ……………………… 1  NO ……………………… 2 | | | YES ……………………… 1  NO ……………………… 2 | YES ……………………… 1  NO ……………………… 2 | YES ……………………… 1  NO ……………………… 2 | |
| Q | 13408 _03 | Is there a measured weight or a comment on status of weight loss documented for the client at the most recent visit? | YES ……………………… 1  NO ……………………… 2 | YES ……………………… 1  NO ……………………… 2 | | | YES ……………………… 1  NO ……………………… 2 | YES ……………………… 1  NO ……………………… 2 | YES ……………………… 1  NO ……………………… 2 | |
| t-HFA | G003 | Is there documentation that the client was assessed for night sweats at the most recent visit? | YES ……………………… 1  NO ……………………… 2 | YES ……………………… 1  NO ……………………… 2 | | | YES ……………………… 1  NO ……………………… 2 | YES ……………………… 1  NO ……………………… 2 | YES ……………………… 1  NO ……………………… 2 | |
| t-HFA | G004 | Is there documentation that the client was screened for TB at the most recent visit, but the documentation did not list the four TB symptoms (cough, fever, night sweats, weight loss)? For example, was the documentation something like: "TB screening completed" or "TB screening negative"? | YES ……………………… 1  NO ……………………… 2 | YES ……………………… 1  NO ……………………… 2 | | | YES ……………………… 1  NO ……………………… 2 | YES ……………………… 1  NO ……………………… 2 | YES ……………………… 1  NO ……………………… 2 | |
| Q | 13408 _08 | What was the client’s TB status at the most recent time this was documented?  IF THE RECORD HAS “LATENT TB”, “NO TB INFECTION”, OR THERE IS NO DOCUMENTATION, THEN SKIP THE NEXT QUESTION (13408_09). | ACTIVE TB .............. 1  LATENT TB ............. 2  NO TB INFECTION… 3  NOT  DOCUMENTED .... 98 | ACTIVE TB .............. 1  LATENT TB ............. 2  NO TB INFECTION… 3  NOT  DOCUMENTED .... 98 | | | ACTIVE TB .............. 1  LATENT TB ............. 2  NO TB INFECTION… 3  NOT  DOCUMENTED .... 98 | ACTIVE TB .............. 1  LATENT TB ............. 2  NO TB INFECTION… 3  NOT  DOCUMENTED .... 98 | ACTIVE TB .............. 1  LATENT TB ............. 2  NO TB INFECTION… 3  NOT  DOCUMENTED .... 98 | |
| Q | 13408 _09 | Is there documentation that the client is currently enrolled in TB treatment? | YES ……………………… 1  NO ……………………… 2 | YES ……………………… 1  NO ……………………… 2 | | | YES ……………………… 1  NO ……………………… 2 | YES ……………………… 1  NO ……………………… 2 | YES ……………………… 1  NO ……………………… 2 | |
| t-HFA | G001 | Was the client’s blood pressure recorded at the most recent visit? | YES ……………………… 1  NO ……………………… 2 | YES ……………………… 1  NO ……………………… 2 | | | YES ……………………… 1  NO ……………………… 2 | YES ……………………… 1  NO ……………………… 2 | YES ……………………… 1  NO ……………………… 2 | |
| t-HFA | G002 | Did the client begin ART at least 12 months ago? | YES ……… 1  NO ……… 2 ➔ 13411 | YES ……… 1  NO ……… 2 ➔ 13411 | | | YES ……… 1  NO ……… 2 ➔ 13411 | YES ……… 1  NO ……… 2 ➔ 13411 | YES ……… 1  NO ……… 2 ➔ 13411 | |
| t-HFA | G003 | If yes, has the patient been on ART at two time points at least 12 months apart?  FOR EXAMPLE, IF THE SURVEY VISIT WAS ON AUGUST 1, 2023, AND THERE WAS DOCUMENTATION THAT THE PATIENT WAS ON ART IN AUGUST 2022 AND JULY 2023, THEN THE RESPONSE FOR THIS QUESTION IS YES. NOTE THAT IF THERE IS CLEAR DOCUMENTATION THAT THE PATIENT HAD STOPPED ART DURING THIS 12-MONTH TIME PERIOD, THEN THE RESPONSE TO THIS QUESTION IS NO. | YES ……………………… 1  NO ……………………… 2  UNCLEAR ……………. 8 | YES ……………………… 1  NO ……………………… 2  UNCLEAR ……………. 8 | | | YES ……………………… 1  NO ……………………… 2  UNCLEAR ……………. 8 | YES ……………………… 1  NO ……………………… 2  UNCLEAR ……………. 8 | YES ……………………… 1  NO ……………………… 2  UNCLEAR ……………. 8 | |
|  |  |  | | | | | | | |  |
| Q | 13411 | ANY ISSUES OR EXPLANATIONS TO DOCUMENT RELATED TO THE RECORD REVIEW | | | | YES ...................................................................................... 1  (13411A) __________________________________________________________  (IF YES, SPECIFY)  NO ....................................................................................... 2 | | | |  |
|  |  | **END OF T-HFA SECTION 7** | | | | | | | |  |

| **Mod** | **No.** | **QUESTION** | | | **RESPONSE** | | | | | **SKIP** |
| --- | --- | --- | --- | --- | --- | --- | --- | --- | --- | --- |
|  |  | **T-HFA SECTION 8. RECORD REVIEW FOR TUBERCULOSIS CLIENTS**  Source: WHO (2022) HHFA Module 3: Quality of care. Questions in this section are used to measure GF indicator RSSH/PP HRH-6 (effective services). | | | | | | | |  |
| Q | 13500 | Does this facility offer any tuberculosis client care services? | | | YES .................................................................................................................... 1  NO ..................................................................................................................... 2 | | | | | ➔ End of section 8 |
| Q | 13502 | **ELIGIBILITY CRITERIA:**  PULMONARY TB ADULT CLIENT (15 OR MORE YEARS OF AGE) ON NATIONAL FIRST LINE TREATMENT AND STARTED TREATMENT AT LEAST 6 MONTHS AGO. THIS MAY INCLUDE CLIENTS WHO HAVE COMPLETED THEIR FULL COURSE OF TREATMENT. EXCLUDE CLIENTS WHO DROPPED OUT PRIOR TO COMPLETING 6 MONTHS OF TREATMENT OR WHO WERE REFERRED ELSEWHERE FOR TREATMENT (E.G., DRUG RESISTANT CASES).  [COUNTRY ADAPT ELIGIBILITY CRITERIA. If there is a desire to conduct the TB record review for additional age groups (i.e., under 5 years, 5-14 years), please duplicate the TB record review and implement the TB record review procedures separately for each age group of interest.]    **SAMPLE SELECTION:**  THE RECORD REVIEW REQUIRES TWO STEPS: (1) IDENTIFYING A SAMPLE OF ELIGIBLE CLIENT RECORDS, AND (2) OBTAINING AND ASSESSING ELIGIBLE RECORDS.    **1. IDENTIFYING A SAMPLE OF ELIGIBLE CLIENT RECORDS:**  THIS STEP REQUIRES ACCESS TO A LIST OF CLIENTS THAT HAVE USED THE FACILITY FOR THE CONDITION ASSESSED IN THE RECORD REVIEW. THE LIST MAY BE FOUND IN A REGISTER, APPOINTMENT BOOK, APPOINTMENT CARD BOX, DATABASE OR OTHER LIST. THIS LIST WILL BE THE SOURCE DOCUMENT FOR THE SAMPLE.    ASK TO BE GIVEN THE SOURCE DOCUMENT/REGISTER AND IDENTIFY THE CURRENT MONTH IN THE PRECEEDING YEAR WHERE CLIENTS CAN BE IDENTIFIED. COUNTING FORWARDS FROM THE BEGINNING OF THE CURRENT MONTH IN THE PRECEEDING YEAR, IDENTIFY 10 CLIENTS WHO MEET THE ELIGIBILITY CRITERIA FOR THIS RECORD REVIEW. CONTINUE COUNTING ALL ELIGIBLE CLIENTS TO A MAXIMUM OF 6 FULL MONTHS, IF NEEDED, TO IDENTIFY CLIENTS MEETING THE ELIGIBILITY CRITERIA. DO NOT EXCEED SIX MONTHS EVEN IF 10 CLIENTS HAVE NOT BEEN IDENTIFIED. COMPLETE A CLIENT SAMPLING LIST (SEE ANNEX 3 “RECORD REVIEW SAMPLING TOOL”) BY RECORDING IDENTIFYING INFORMATION FOR THE ELIGIBLE CLIENTS. THE GOAL IS TO IDENTIFY 10 ELIGIBLE CLIENTS.  **IF 10 ELIGIBLE CLIENTS HAVE BEEN IDENTIFIED:** THE COMPLETED CLIENT SAMPLING LIST HAS A SAMPLING NUMBER BETWEEN 1 AND 10 FOR EACH CLIENT. TO SELECT THE 5 CLIENTS WHOSE INFORMATION WILL BE ENTERED INTO THE T-HFA QUESTIONNAIRE, TOSS A COIN. ONE SIDE WILL MEAN YOU SELECT CLIENTS WITH SAMPLING NUMBERS 1, 3, 5, 7, AND 9; THE OTHER SIDE WILL MEAN YOU SELECT CLIENTS 2, 4, 6, 8, AND 10.  **IF FEWER THAN 10 ELIGIBLE CLIENTS HAVE BEEN IDENTIFIED:** TO SELECT THE 5 CLIENTS WHOSE INFORMATION WILL BE ENTERED INTO THE T-HFA QUESTIONNAIRE, FOLLOW THE INSTRUCTIONS IN THE RECORD REVIEW SAMPLING TOOL (ANNEX 3).    ***2. OBTAINING AND ASSESSING THE ELIGIBLE RECORDS:***  USING THE IDENTIFYING INFORMATION FOR EACH OF THE 5 CLIENTS SELECTED FOR THE SAMPLE, ASK TO SEE THE INDIVIDUAL RECORD FOR THESE CLIENTS. A “RECORD” REFERS TO A DOCUMENT THAT CONTAINS INFORMATION ON INDIVIDUAL CLIENT CARE. THIS INFORMATION MAY BE CONTAINED IN A REGISTER, INDIVIDUAL CLIENT CARD/FILE/CHART, ETC., BUT MUST BE RECORDED AT THE CLIENT LEVEL AND PROVIDE SPECIFICS ON THE CONTENT OF CARE DELIVERED TO THE INDIVIDUAL. IN SOME CASES, THE INFORMATION WILL BE AVAILABLE IN A REGISTER AND INDIVIDUAL CARDS/CHARTS WILL NOT NEED TO BE LOCATED.  IF THE INDIVIDUAL CLIENT CARD/FILE/CHART FOR A SELECTED INDIVIDUAL CANNOT BE LOCATED, THE INDIVIDUAL MAY BE REPLACED WITH THE NEXT ELIGIBLE PERSON ON THE CLIENT SAMPLING LIST WHO HAS NOT ALREADY BEEN SELECTED. FOR EXAMPLE, CONSIDER THE SCENARIO IN WHICH YOU HAVE IDENTIFIED 10 ELIGIBLE CLIENTS, AND YOU HAVE FLIPPED A COIN AND SELECTED CLIENTS 1, 3, 5, 7, AND 9. IF THE RECORD FOR CLIENT 3 CANNOT BE LOCATED, THEN REPLACE CLIENT 3 WITH CLIENT 4. REMEMBER THAT THE GOAL IS TO SELECT 5 CLIENTS AND ENTER THEIR INFORMATION INTO THE T-HFA QUESTIONNAIRE.  [COUNTRY ADAPT STARTING MONTH AND DIRECTION OF COUNTING ACCORDING TO REGISTER FORMAT IN USE] | | | | | | | |  |
| Q | 13502 _01 | NUMBER OF DIFFERENT MONTHS FROM WHICH THE SAMPLE WAS SELECTED | | | ___ NUMBER OF MONTHS | | | | |  |
| Q | 13502 _02 | NUMBER OF ELIGIBLE CLIENTS IDENTIFIED | | | ___ ___ NUMBER OF ELIGIBLE CLIENTS    NO ELIGIBLE CLIENTS IDENTIFIED ............................................... 00 | | | | | ➔ 13513 |
| tHFA | 13502 _02b | NUMBER OF ELIGIBLE CLIENTS SAMPLED | | | ___ ___ NUMBER OF ELIGIBLE CLIENTS SAMPLED | | | | |  |
| Q | 13502 _03 | NUMBER OF ORIGINALLY SELECTED SAMPLE CLIENTS REPLACED DUE TO MISSING INDIVIDUAL CLIENT RECORDS  **NOTE: RECORD AFTER COMPLETION OF RECORD REVIEW** | | | ___ NUMBER REPLACED  NONE ............................................................................................ 0 | | | | |  |
| Q | 13502 _04 | SOURCE DATA FOR SAMPLE SELECTION  IF MORE THAN ONE SOURCE IS USED TO IDENTIFY THE SAMPLE, CIRCLE ALL THAT APPLY.  **NOTE:** SOURCE DOCUMENT REFERS TO REGISTERS AND RECORDS THAT CONTAIN INFORMATION ON CLIENTS WHO HAVE RECEIVED TUBERCULOSIS SERVICES AND CAN SERVE AS A SAMPLING LIST FOR THE RECORD REVIEW. | | | TB CASE REGISTER .............................................................................................. A  INDIVIDUAL TB CLIENT CARDS/CHARTS/RECORDS ............................................ B  LABORATORY REGISTER ..................................................................................... C  OTHER (13502_05A) ______________________________________________ X  (SPECIFY) | | | | |  |
|  |  |  | | |  | | | | |  |
| Q | 13506 | **TREATMENT** | | | | | | | |  |
|  |  |  | CLIENT  1 | CLIENT  2 | | | CLIENT  3 | CLIENT  4 | CLIENT  5 | |
| tHFA | RR_TB_Begin | ENUMERATOR, DO YOU HAVE TB CLIENT RECORDS WITH YOU FOR THE RECORD REVIEW? | YES ……………………… 1  NO ……………………… 2 | YES ……………………… 1  NO ……………………… 2 | | | YES ……………………… 1  NO ……………………… 2 | YES ……………………… 1  NO ……………………… 2 | YES ……………………… 1  NO ……………………… 2 | |
| tHFA | 13500_Client_ID | Client ID from the record register | ………………………………… | ………………………………… | | | ………………………………… | ………………………………… | ………………………………… | |
| tHFA | 13500_Client_Name | Client Name from the record register | ………………………………… | ………………………………… | | | ………………………………… | ………………………………… | ………………………………… | |
| Q | 13506  _04 | Was the client successfully treated (either documented as cured or completed treatment at the end of the treatment period)? | YES, CLIENT WAS CURED  OR COMPLETED TREATMENT .................... 1    NO, CLIENT FAILED TREATMENT ..................... 2  CLIENT STILL ON TREATMENT .................... 3  NOT DOCUMENTED ….... 98 | YES, CLIENT WAS CURED  OR COMPLETED TREATMENT .................... 1    NO, CLIENT FAILED TREATMENT ..................... 2  CLIENT STILL ON TREATMENT .................... 3  NOT DOCUMENTED ….... 98 | | | YES, CLIENT WAS CURED  OR COMPLETED  TREATMENT .................... 1    NO, CLIENT FAILED TREATMENT ..................... 2  CLIENT STILL ON TREATMENT .................... 3  NOT DOCUMENTED ….... 98 | YES, CLIENT WAS CURED  OR COMPLETED  TREATMENT .................... 1    NO, CLIENT FAILED TREATMENT ..................... 2  CLIENT STILL ON  TREATMENT .................... 3  NOT DOCUMENTED ….... 98 | YES, CLIENT WAS CURED  OR COMPLETED TREATMENT .................... 1    NO, CLIENT FAILED TREATMENT ..................... 2  CLIENT STILL ON TREATMENT .................... 3  NOT DOCUMENTED ….... 98 | |
|  |  |  |  |  | | |  |  |  | |
| Q | 13507 | **HIV TESTING** | | | | | | | | |
| Q | 13507  _01 | Was an HIV test result documented for the client? | YES ………………………………. 1  NO ……………………………….. 2 | YES ………………………………. 1  NO ……………………………….. 2 | | | YES ………………………………. 1  NO ……………………………….. 2 | YES ………………………………. 1  NO ……………………………….. 2 | YES ………………………………. 1  NO ……………………………….. 2 | |
|  |  |  |  |  | | |  |  |  | |
| Q | 13510 | **TB SYMPTOMS** | | | | | | | | |
| Q | 13510  _01 | Is a clinical assessment of changes in symptoms documented every clinical visit? | YES ………………………………. 1  NO ……………………………….. 2 | YES ………………………………. 1  NO ……………………………….. 2 | | | YES ………………………………. 1  NO ……………………………….. 2 | YES ………………………………. 1  NO ……………………………….. 2 | YES ………………………………. 1  NO ……………………………….. 2 | |
| Q | 13513 | ANY ISSUES OR EXPLANATIONS TO DOCUMENT RELATED TO THE RECORD REVIEW | | | | YES ...................................................................................... 1  (13513A) ___________________________________________________________  (IF YES, SPECIFY)  NO ....................................................................................... 2 | | | |  |
|  |  | **END OF T-HFA SECTION 8** | | | | | | | |  |

**Country Specific Questions**

| No. | Country Specific Questions |
| --- | --- |
| CS_Q1 | Question 1 |
| CS_R1 | Response for ${CS_Q1} |
| CS_Q2 | Question 2 |
| CS_R2 | Response for ${CS_Q2} |
| CS_Q3 | Question 3 |
| CS_R3 | Response for ${CS_Q3} |
| CS_Q4 | Question 4 |
| CS_R4 | Response for ${CS_Q4} |
| CS_Q5 | Question 5 |
| CS_R5 | Response for ${CS_Q5} |
| CS_Q6 | Question 6 |
| CS_R6 | Response for ${CS_Q6} |
| CS_Q7 | Question 7 |
| CS_R7 | Response for ${CS_Q7} |
| CS_Q8 | Question 8 |
| CS_R8 | Response for ${CS_Q8} |
| CS_Q9 | Question 9 |
| CS_R9 | Response for ${CS_Q9} |
| CS_Q10 | Question 10 |
| CS_R10 | Response for ${CS_Q10} |
| CS_R10 | Response for ${CS_Q10} |

| **Mod** | **No.** | **QUESTION** | **RESPONSE** | | | | | | | | | | | | |
| --- | --- | --- | --- | --- | --- | --- | --- | --- | --- | --- | --- | --- | --- | --- | --- |
|  |  |  |  | | | | | | | | | | | | |
| Q | 107 | Interview date  * RESULT CODE  1 = INTERVIEW STARTED BUT NOT COMPLETED  2 = POSTPONED  3 = FACILITY CLOSED  4 = FACILITY DESTROYED  5 = FACILITY NOT FOUND  6 = OTHER  7 = INTERVIEW COMPLETED |  | FACILITY VISIT(S) | | | | | | | | | | |  |
|  |  |  |  | Visit  No. | DATE | | | | | | | | Inter-  viewer  code | Result  code* |  |
|  |  |  |  |  | DD | | MM | | YYYY | | | |  |  |  |
|  |  |  |  | 1 |  |  |  |  |  |  |  |  |  |  |  |
|  |  |  |  | 2 |  |  |  |  |  |  |  |  |  |  |  |
|  |  |  |  | 3 |  |  |  |  |  |  |  |  |  |  |  |
|  |  |  |  |  | | | | | | | | | | |  |
|  |  |  |  | | | | | | | | | | | | |
|  |  |  | | | | | | | | | | | | | |
|  |  | **END OF T-HFA QUESTIONNAIRE** | | | | | | | | | | | | | |

**Country specific EDL list code for 30 tests**

In the questions **EDL_TEST_##** replaced with the actual tests for the country EDL list

| Code | Questions |
| --- | --- |
| EDL_TEST_01 | EDL_TEST_01 |
| EDL_RESPONSE_01 | ASK THE LABORATORY STAFF PERSON IF <font color="blue">${EDL_TEST_01}</font> IS CONDUCTED |
| EDL_TEST_02 | EDL_TEST_02 |
| EDL_RESPONSE_02 | ASK THE LABORATORY STAFF PERSON IF <font color="blue">${EDL_TEST_02}</font> IS CONDUCTED |
| EDL_TEST_03 | EDL_TEST_03 |
| EDL_RESPONSE_03 | ASK THE LABORATORY STAFF PERSON IF <font color="blue">${EDL_TEST_03}</font> IS CONDUCTED |
| EDL_TEST_04 | EDL_TEST_04 |
| EDL_RESPONSE_04 | ASK THE LABORATORY STAFF PERSON IF <font color="blue">${EDL_TEST_04}</font> IS CONDUCTED |
| EDL_TEST_05 | EDL_TEST_05 |
| EDL_RESPONSE_05 | ASK THE LABORATORY STAFF PERSON IF <font color="blue">${EDL_TEST_05}</font> IS CONDUCTED |
| EDL_TEST_06 | EDL_TEST_06 |
| EDL_RESPONSE_06 | ASK THE LABORATORY STAFF PERSON IF <font color="blue">${EDL_TEST_06}</font> IS CONDUCTED |
| EDL_TEST_07 | EDL_TEST_07 |
| EDL_RESPONSE_07 | ASK THE LABORATORY STAFF PERSON IF <font color="blue">${EDL_TEST_07}</font> IS CONDUCTED |
| EDL_TEST_08 | EDL_TEST_08 |
| EDL_RESPONSE_08 | ASK THE LABORATORY STAFF PERSON IF <font color="blue">${EDL_TEST_08}</font> IS CONDUCTED |
| EDL_TEST_09 | EDL_TEST_09 |
| EDL_RESPONSE_09 | ASK THE LABORATORY STAFF PERSON IF <font color="blue">${EDL_TEST_09}</font> IS CONDUCTED |
| EDL_TEST_10 | EDL_TEST_10 |
| EDL_RESPONSE_10 | ASK THE LABORATORY STAFF PERSON IF <font color="blue">${EDL_TEST_10}</font> IS CONDUCTED |
| EDL_TEST_11 | EDL_TEST_11 |
| EDL_RESPONSE_11 | ASK THE LABORATORY STAFF PERSON IF <font color="blue">${EDL_TEST_11}</font> IS CONDUCTED |
| EDL_TEST_12 | EDL_TEST_12 |
| Code | Questions |
| EDL_RESPONSE_12 | ASK THE LABORATORY STAFF PERSON IF <font color="blue">${EDL_TEST_12}</font> IS CONDUCTED |
| EDL_TEST_13 | EDL_TEST_13 |
| EDL_RESPONSE_13 | ASK THE LABORATORY STAFF PERSON IF <font color="blue">${EDL_TEST_13}</font> IS CONDUCTED |
| EDL_TEST_14 | EDL_TEST_14 |
| EDL_RESPONSE_14 | ASK THE LABORATORY STAFF PERSON IF <font color="blue">${EDL_TEST_14}</font> IS CONDUCTED |
| EDL_TEST_15 | EDL_TEST_15 |
| EDL_RESPONSE_15 | ASK THE LABORATORY STAFF PERSON IF <font color="blue">${EDL_TEST_15}</font> IS CONDUCTED |
| EDL_TEST_16 | EDL_TEST_16 |
| EDL_RESPONSE_16 | ASK THE LABORATORY STAFF PERSON IF <font color="blue">${EDL_TEST_16}</font> IS CONDUCTED |
| EDL_TEST_17 | EDL_TEST_17 |
| EDL_RESPONSE_17 | ASK THE LABORATORY STAFF PERSON IF <font color="blue">${EDL_TEST_17}</font> IS CONDUCTED |
| EDL_TEST_18 | EDL_TEST_18 |
| EDL_RESPONSE_18 | ASK THE LABORATORY STAFF PERSON IF <font color="blue">${EDL_TEST_18}</font> IS CONDUCTED |
| EDL_TEST_19 | EDL_TEST_19 |
| EDL_RESPONSE_19 | ASK THE LABORATORY STAFF PERSON IF <font color="blue">${EDL_TEST_19}</font> IS CONDUCTED |
| EDL_TEST_20 | EDL_TEST_20 |
| EDL_RESPONSE_20 | ASK THE LABORATORY STAFF PERSON IF <font color="blue">${EDL_TEST_20}</font> IS CONDUCTED |
| EDL_TEST_21 | EDL_TEST_21 |
| EDL_RESPONSE_21 | ASK THE LABORATORY STAFF PERSON IF <font color="blue">${EDL_TEST_21}</font> IS CONDUCTED |
| EDL_TEST_22 | EDL_TEST_22 |
| EDL_RESPONSE_22 | ASK THE LABORATORY STAFF PERSON IF <font color="blue">${EDL_TEST_22}</font> IS CONDUCTED |
| EDL_TEST_23 | EDL_TEST_23 |
| EDL_RESPONSE_23 | ASK THE LABORATORY STAFF PERSON IF <font color="blue">${EDL_TEST_23}</font> IS CONDUCTED |
| EDL_TEST_24 | EDL_TEST_24 |
| EDL_RESPONSE_24 | ASK THE LABORATORY STAFF PERSON IF <font color="blue">${EDL_TEST_24}</font> IS CONDUCTED |
| EDL_TEST_25 | EDL_TEST_25 |
| EDL_RESPONSE_25 | ASK THE LABORATORY STAFF PERSON IF <font color="blue">${EDL_TEST_25}</font> IS CONDUCTED |
| EDL_TEST_26 | EDL_TEST_26 |
| Code | Questions |
| EDL_RESPONSE_26 | ASK THE LABORATORY STAFF PERSON IF <font color="blue">${EDL_TEST_26}</font> IS CONDUCTED |
| EDL_TEST_27 | EDL_TEST_27 |
| EDL_RESPONSE_27 | ASK THE LABORATORY STAFF PERSON IF <font color="blue">${EDL_TEST_27}</font> IS CONDUCTED |
| EDL_TEST_28 | EDL_TEST_28 |
| EDL_RESPONSE_28 | ASK THE LABORATORY STAFF PERSON IF <font color="blue">${EDL_TEST_28}</font> IS CONDUCTED |
| EDL_TEST_29 | EDL_TEST_29 |
| EDL_RESPONSE_29 | ASK THE LABORATORY STAFF PERSON IF <font color="blue">${EDL_TEST_29}</font> IS CONDUCTED |
| EDL_TEST_30 | EDL_TEST_30 |
| EDL_RESPONSE_30 | ASK THE LABORATORY STAFF PERSON IF <font color="blue">${EDL_TEST_30}</font> IS CONDUCTED |
