# Supplementary material for: Design and implementation of a multi-country targeted health facility assessment to measure integrated, people-centered, quality of services
Source: PLOS Glob Public Health. 2026 Aug 19;6(8):e0005736. doi: 10.1371/journal.pgph.0005736 (PMC13489417; doi:10.1371/journal.pgph.0005736)
Supplement: Supplementary file 3 [file pgph.0005736.s003.docx]

**S1 Table: IRB Approval authority and reference number by Country**

| **Sno** | **Country Name** | **IRB Approval Authority** | **IRB Reference number** |
| --- | --- | --- | --- |
| 1 | Burkina Faso | Health Research Ethics Committee | DELIBERATION N° 2024-04-89 |
| 2 | Cameroon | National Ethical Committee For Research On Human Health | N° 2024/04/4660/CE/CNERSH/SP |
| 3 | Chad | National Bioethics Committee of Chad (CNBT) | N036/MESRSFP/SE/SG/CNBT/SG/2024 |
| 4 | Congo | Institutional Ethics Committee – FCRM | Avis nº 054/CEI/FCRM/2024 |
| 5 | Congo (Democratic Republic) | University of Kinshasa School of Public Health Ethics Committee | ESP/CE/209/2024 |
| 6 | Gambia | Ethical Approval - Not Required |  |
| 7 | Ghana | Ghana Health Service Ethics Review Committee | GHS-ERC: 024/03/24 |
| 8 | Guinea-Bissau | National Committee on Health Research Ethics (CNES) | N° Ref. 159/CNES/INASA/2024 |
| 9 | Liberia | Atlantic Center for Research and Evaluation Institutional Review Board (ACRE IRB) | FWA00032198 |
| 10 | Madagascar | Ethical Approval - Not Required |  |
| 11 | Malawi | National Health Sciences Research Committee ((NHSRC) | MED/4/36c approval number 4324 |
| 12 | Mali | Ethics Committee for Health and Life Sciences (CNESS) | Décision n° 2024 006 /MSDS-CNESS du 26 JUIN 2024 |
| 13 | Mozambique | National Bioethics Committee for Health | 427/CNBS/24 |
| 14 | Niger | National Ethics Committee for Health Research (CNERS) | DELIBERATION N°29/2024/CNERS |
| 15 | Nigeria | Ethical Approval - Not Required |  |
| 16 | Senegal | National Ethics Committee for Health Research (CNERS) | 00000070/MSAS/CNERS/SP |
| 17 | Tanzania (United Republic) | National Institute for Medical Research | NIMR/HQ/R.8a/Vol.IX/4683 |
| 18 | Uganda | Ethical Approval - Not Required |  |
