# Supplementary material for: Design and implementation of a multi-country targeted health facility assessment to measure integrated, people-centered, quality of services
Source: PLOS Glob Public Health. 2026 Aug 19;6(8):e0005736. doi: 10.1371/journal.pgph.0005736 (PMC13489417; doi:10.1371/journal.pgph.0005736)
Supplement: Supplementary file 4 [file pgph.0005736.s005.docx]

Dear Editor,

Thank you for the peer review comments. The table below provides an itemized response to each comment provided by the 2 reviewers. Thanks,

Shivam

| **Journal Requirements** | | |
| --- | --- | --- |
| Comment | | Author Response |
| 1. Please include a complete copy of PLOS’ questionnaire on inclusivity in global research in your revised manuscript. Our policy for research in this area aims to improve transparency in the reporting of research performed outside of researchers’ own country or community. The policy applies to researchers who have travelled to a different country to conduct research, research with Indigenous populations or their lands, and research on cultural artefacts. The questionnaire can also be requested at the journal’s discretion for any other submissions, even if these conditions are not met.  Please find more information on the policy and a link to download a blank copy of the questionnaire here: <https://journals.plos.org/globalpublichealth/s/best-practices-in-research-reporting>. Please upload a completed version of your questionnaire as Supporting Information when you resubmit your manuscript. | | Thank you for the comment. We have completed the questionnaire and uploaded it as supporting information. |
| 2. Please amend your detailed Financial Disclosure statement. This is published with the article. It must therefore be completed in full sentences and contain the exact wording you wish to be published.  i. Please clarify all sources of financial support for your study. List the grants, grant numbers, and organizations that funded your study, including funding received from your institution. Please note that suppliers of material support, including research materials, should be recognized in the Acknowledgements section rather than in the Financial Disclosure.  ii. State the initials, alongside each funding source, of each author to receive each grant. For example: "This work was supported by the National Institutes of Health (####### to AM; ###### to CJ) and the National Science Foundation (###### to AM)." iii. State what role the funders took in the study. If the funders had no role in your study, please state: “The funders had no role in study design, data collection and analysis, decision to publish, or preparation of the manuscript.” iv. If any authors received a salary from any of your funders, please state which authors and which funders. | | Thank you for the comment. We revised the financial disclosure and added it to the manuscript.  The design data collection, analyses and interpretation of the targeted health facility assessment (tHFA) project was funded by the Global Fund to fight HIV, Tuberculosis and Malaria through its funds for monitoring of country programs for the grants that the fund disburses to countries. The work was supported by the Gates Foundation (Award number Opp1203694/ Inv-00736 to the Global Fund for SG, JK, RJ, PJ & RR). |
| 3. In the online submission form, you indicated that “The manuscript provides full methodological details of the Targeted Health Facility Assessment (tHFA). Copies of the tHFA questionnaire modules and related materials can be provided upon reasonable request to the corresponding author.”.  All PLOS journals now require all data underlying the findings described in their manuscript to be freely available to other researchers, either  1. In a public repository,  2. Within the manuscript itself, or  3. Uploaded as supplementary information.  This policy applies to all data except where public deposition would breach compliance with the protocol approved by your research ethics board. If your data cannot be made publicly available for ethical or legal reasons (e.g., public availability would compromise patient privacy), please explain your reasons by return email and your exemption request will be escalated to the editor for approval. Your exemption request will be handled independently and will not hold up the peer review process, but will need to be resolved should your manuscript be accepted for publication. One of the Editorial team will then be in touch if there are any issues. | | We thank the editorial team for this reminder. We have uploaded the tHFA questionnaire modules and the detailed indicator methodology document as supplementary information accompanying the manuscript, in compliance with PLOS data availability requirements. |
| 4. Please ensure that your Ethics Statement is available in its entirety at the beginning of your Methods section, under a subheading 'Ethics Statement'. | | We have added an 'Ethics Statement' subheading at the beginning of the Methods section with the full ethics text and removed the duplicate 'Ethical approvals' subsection from later in the Methods. |
| **Comments from Reviewer #1** | | |
| A well-written Ms which provides clear descriptions of the rationale and basis of the study. The use of established norms and standards is welcomed. **While the 11 programme indicators are appropriately detailed, it would assist the reader to have the key information related to these indicators in a tabular format for ease of reference.** | We thank the reviewer for this suggestion. We have replaced the narrative descriptions of the 11 indicators with a structured table (Table 2) that retains all original content including the rationale, components measured, and data collection method for each indicator in a format that is easier to navigate. The detailed indicator computation documentation and the full tHFA questionnaire are provided as online supplementary annexes (Annexes 1 and 2) | |
| The sampling strategy is acceptable and appropriate. The details related to ensuring quality data collection is notable, including embedding and employing independent data quality assurance procedures | We thank the reviewer for this positive feedback on the sampling strategy and data quality assurance procedures. | |
| While the MS is focused on describing the tHFA as a pragmatic and scalable approach to monitor integrated service delivery and people-centered quality of care, there are no formal results presented from this activity. Nevertheless, it does an admirable job of describing the design and implementation protocol for measuring integrated people-centred care. **What appears to be missing is an evaluation framework that will assist in capturing the functionality of the tHFA as a monitoring tool.** | We thank the reviewer for this observation. We have added a paragraph in the discussion noting that the baseline tHFA results are being written up in a separate manuscript. We have also introduced a brief evaluation framework that will be applied to future rounds of the tHFA to assess its effectiveness as a monitoring tool, covering four dimensions: data quality, cost-efficiency, use of results, and sustainability and country ownership. | |
| The Discussion section refers to findings not evident in the MS. For example, on Pp 19, lines 367-372, reference is made to the use of indicators and data collection methods as facilitating comparability across settings while maintaining relevance to country-specific systems is done so in the abstract. Similar statements about tHFA being an agile and scalable platform for integrated service delivery and people-centred quality of care in LMICS in the conclusion to the study cannot be verified without reference to any evaluation data. | We thank the reviewer for this important observation. We have revised the abstract, discussion, and conclusion sections to clearly distinguish between what the tHFA has demonstrably achieved (design, validation, and baseline implementation across 18 countries and 2,272 facilities) and what it is designed or intended to achieve in terms of downstream outcomes. Claims about impacts on decision-making, accountability, and health system strengthening have been reframed using language such as "designed to," "intended to," and "positioned to," reflecting that these outcomes will be assessed through planned follow-up rounds of the tHFA. | |
| **Comments from Reviewer #2** | | |
| I congratulate the authors on a clearly written paper which tackles the fundamental challenge of assessing the ability of health facilities to provide patient-centered care. My most substantial comments concern Tables 1 and 3, and the remaining are minor suggestions | We thank the reviewer for the positive feedback and for the careful reading of the manuscript. The comments are addressed below. | |
| Table 1 is the heart of the paper and provides substantial information, but could benefit from some additional clarifications and perhaps an additional column or two. I think it would be useful to make explicit the method of data collection in a separate column. For example, KPIS1.1 is record review and KPIS1.2 is patient exit interview. The record review is included in the attributes of the first domain, but not in the second. If the authors consistently pull these out into a separate column, it would improve consistency of information and interpretability by the reader. That column could also include an indication of the quantity of data reviewed or collected. **The authors state later in the paper the number of interviews conducted but I don’t think there was information on how many charts or records were reviewed for the quantitative indicators**. In Table 1, or maybe in a separate table in SI, the authors could provide some information on how terms are defined. **For example, the definitions of communication and responsiveness raise questions as the conception of these by patients may vary a bit from setting to setting and it would be helpful for the reader to be able to understand more about how these components are assessed**. Similarly, terms which could be more explicitly defined in the table include: 1) "supportive supervision" under KPIS2 raises questions about supervision by whom and of whom. Being as detailed as possible with titles will help the reader understand the importance of the work; 2) "no stock outs" under KPIS5. | We thank the reviewer for these helpful suggestions. We have made the following changes: (1) corrected "Responsiveness" to "Respect" in the Patient Centeredness component of KPI S1, which accurately reflects the survey question used; (2) expanded the descriptions of "supportive supervision" under KPI S2 and KPI S5 to clarify that this refers to supervision by an external supervisor for KPI S2, and by facility-based or district health staff for KPI S5; and (3) expanded "no stockouts" under KPI S5 to specify the types of commodities, equipment, and job aids covered. Regarding the suggestion to add a data collection method column with sample sizes, we have addressed this in the newly added Table 2, which accompanies Table 1 and includes a dedicated column specifying the data collection method and sample size for each indicator. We felt this was better placed in Table 2 rather than Table 1, as adding a fourth column to the already detailed Table 1 would reduce readability. | |
| In the methodology section, line 115, please make explicit how you are defining health facilities. You have the detailed definition later in the paper, but defining it earlier on will help guide the reader. | We have added an explicit definition of eligible health facilities at the beginning of the Methodology section. The definition covering facility type (public and, in eight countries, private sector), service requirement (ANC and/or HTM services), levels of care (primary, secondary, tertiary), and exclusion criteria (specialized-only facilities), was previously embedded in the sampling subsection and has now been brought forward to orient the reader earlier. | |
| Line 203 is missing a period. | Thank you for catching this. A period has been added after "(Table 3)" in the Sampling section. | |
| In the section beginning on line 213 (7. Programmatic Indicator 3: Health Sector Planning and Governance for Integrated People-Centred Services (HSG)-1.1: Availability of Clinical Guidelines), the authors discuss the review of availability of clinical guidelines. This was the first metric where I paused to consider how good the evidence is on whether the indicator (clinical guidelines availability) is actually associated with the outcomes of interest (integrated, people-centered care). You may want to consider expanding the introduction a bit to include the literature in support of the metrics, or this may be separate work that you could pursue independent of this paper. | We thank the reviewer for this important observation. We have added a sentence in the Introduction acknowledging that structural indicators are necessary but not sufficient conditions for quality care, citing Leslie et al. (2017). In the Discussion, we have added two sentences clarifying that structural indicators in the tHFA are best interpreted directionally, low scores reliably signal system weaknesses, whereas high scores indicate enabling conditions but do not, on their own, confirm that integrated, people-centred care is being delivered. | |
| Sampling: Table 3 and the text in lines 293-301 may be more easily interpreted as a figure or flow chart. Table 3, if it remains, could use clarification in the column headings and information on how the sampling strategy aligns with the overall sample size. For example, the word Domains also appears in Table 1, and they have different meanings across the table, but the word is ambiguous and difficult to interpret without careful reading of the accompanying text. | We thank the reviewer for these suggestions. We have replaced the ambiguous term "Domain" with "IPCQS area" in Table 1 to distinguish it from its use in Table 3, where "Domains" has been updated to "geographic domains" throughout. Column headings in Table 3 have been clarified, and a brief explanatory note has been added to make the relationship between the sampling strategy and overall sample size more transparent. A flow chart illustrating the sampling has been added (Figure 1). | |
| Data collection and quality assurance: In Table 4, you may want to add one additional column to indicate the number of days of data collection. If the point in Table 4 is mainly to convey the overlap in data collection periods, some kind of figure like a gantt chart may do that more clearly with one row per data collection activity (the current table rows). | We have added a gantt chart indicating the number of days of data collection and overlap in data collection periods (Figure 2). | |
| Table 5 would be more interpretable as 3 columns without row markings as I think I should read it up and down, but not across. If the rows are useful, an initial column could give the row topic area to help guide the reader. | We agree with the reviewer's suggestion. The table has been reformatted from a row-based grid into three columns i.e. Before data collection, During data collection, and After data collection, each with a bulleted list of activities, making it easier to read vertically within each phase. | |
| Discussion: Line 385 discusses how this is a cost-efficient data collection tool. Do you envision this as being something that district health officers could do in the future or would this always sit with something like a central MOH team or the department of statistics? It would be interesting to add a comment on the long term vision for uptake. | In the long term, the tHFA is envisioned as a tool that ministries of health and national statistics offices can institutionalize within their routine health facility surveys, building national capacity for periodic, self-financed monitoring of integrated,people-centred service delivery. As country ownership deepens and data collection costs are further optimized, adoption by sub-national health management teams for district-level decision-making would further strengthen its utility for sub-national health management. | |
| The paper talks repeatedly about patient-centered experience, but the methods section don't mention the inclusion of patients in the development of the tools. If they were involved, please be explicit. If there was no role of patients in the development, this should be noted in the limitations section and the authors could reflect on how this impacts the results. | We thank the reviewer for this important observation. While tHFA questionnaire was pilot tested before in-country rollout, patients were not directly involved in the development of the tHFA questions related to patient-centeredness (KPI S1, Dimension 2). These questions are drawn from the WHO PREMs and WHO HHFA, both globally validated instruments, providing confidence that these items have been tested across diverse LMIC settings. Greater engagement of patients and community representatives in adapting and refining the tool for specific country contexts remains an important priority for future rounds. | |
| Figure Resubmissions | | |
| While revising your submission, we strongly recommend that you use PLOS’s NAAS tool (<https://ngplosjournals.pagemajik.ai/artanalysis>) to test your figure files. NAAS can convert your figure files to the TIFF file type and meet basic requirements (such as print size, resolution), or provide you with a report on issues that do not meet our requirements and that NAAS cannot fix. After uploading your figures to PLOS’s NAAS tool - <https://ngplosjournals.pagemajik.ai/artanalysis>, NAAS will process the files provided and display the results in the "Uploaded Files" section of the page as the processing is complete. If the uploaded figures meet our requirements (or NAAS is able to fix the files to meet our requirements), the figure will be marked as "fixed" above. If NAAS is unable to fix the files, a red "failed" label will appear above. When NAAS has confirmed that the figure files meet our requirements, please download the file via the download option, and include these NAAS processed figure files when submitting your revised manuscript. | Thank you for the comment. We used the NAAS tool and uploaded figures accordingly. | |
