# Supplementary material for: Design and implementation of a multi-country targeted health facility assessment to measure integrated, people-centered, quality of services
Source: PLOS Glob Public Health. 2026 Aug 19;6(8):e0005736. doi: 10.1371/journal.pgph.0005736 (PMC13489417; doi:10.1371/journal.pgph.0005736)
Supplement: Supplementary file 5 [file pgph.0005736.s006.docx]

Dear Editor,

Thank you for the peer review comments. The table below provides the response to the comment provided by reviewer #1. Thanks,

Shivam

| **Journal Requirements** | |
| --- | --- |
| Comment | Author Response |
| While the authors indicate that they have removed statements that reflect impact, the concluding statement states: "The tHFA is a pragmatic, agile, scalable platform to monitor integrated service delivery and people-centred quality of care in LMICs. With a focused set 456 of process- and outcome-oriented measures, inclusion of CHWs, and alignment with the Global Fund Strategy (2023–2028), it is designed to supports 457 decision-making, programme monitoring, and health-system strengthening.” | We thank the reviewer for this observation. We agree that the concluding statement contained language that could be read as implying demonstrated impact, despite this not being the intent. We have revised the conclusion to consistently frame the tHFA's contributions in terms of design intent and potential rather than achieved outcomes, in line with the changes made elsewhere in the manuscript. Specifically, we replaced assertive phrasing (e.g., "is a platform to monitor," "adapts to country priorities," "maintaining KPI comparability") with intent-oriented language ("is designed to," "is intended to adapt," "aiming to maintain").  Upon review, we identified similar instances of impact-implying language elsewhere in the manuscript and revised these for consistency as well. |
