# Supplementary material for: Design and implementation of a multi-country targeted health facility assessment to measure integrated, people-centered, quality of services
Source: PLOS Glob Public Health. 2026 Aug 19;6(8):e0005736. doi: 10.1371/journal.pgph.0005736 (PMC13489417; doi:10.1371/journal.pgph.0005736)
Supplement: Peer review history

# Peer review: Decision Letter 0

**DOI:** 10.1371/journal.pgph.0005736.r001

**Authors:** Emma Campbell

13 Apr 2026

PGPH-D-25-03786

Design and implementation of a multi-country targeted health facility assessment to measure integrated, people-centered, quality of services

PLOS Global Public Health

Dear Dr. Gupta,

Thank you for submitting your manuscript to PLOS Global Public Health. After careful consideration, we feel that it has merit but does not fully meet PLOS Global Public Health’s publication criteria as it currently stands. Therefore, we invite you to submit a revised version of the manuscript that addresses the points raised during the review process.

The manuscript has been assessed by 2 reviewers and their comments are available below. Please review their assessments and make the appropriate revisions to address their concerns. The reviewers have noted that in its current form the manuscript is essentially a description of an implementation protocol, to thoroughly improve the publication, it needs to include a comprehensive evaluation framework that will unpack the credibility of the tHFA as a scalable platform and integrate clear results assessing the methodology and implementation of the protocol.

Please submit your revised manuscript by May 28 2026 11:59PM. If you will need more time than this to complete your revisions, please reply to this message or contact the journal office at globalpubhealth@plos.org. When you're ready to submit your revision, log on to https://www.editorialmanager.com/pgph/ and select the 'Submissions Needing Revision' folder to locate your manuscript file.

Please include the following items when submitting your revised manuscript:

- A letter that responds to each point raised by the editor and reviewer(s). You should upload this letter as a separate file labeled 'Response to Reviewers'.
- A marked-up copy of your manuscript that highlights changes made to the original version. You should upload this as a separate file labeled 'Revised Manuscript with Track Changes'.
- An unmarked version of your revised paper without tracked changes. You should upload this as a separate file labeled 'Manuscript'.

Guidelines for resubmitting your figure files are available below the reviewer comments at the end of this letter.

As the corresponding author, your ORCID iD is verified in the submission system and will appear in the published article. PLOS supports the use of ORCID, and we encourage all coauthors to register for an ORCID iD and use it as well. Please encourage your coauthors to verify their ORCID iD within the submission system before final acceptance, as unverified ORCID iDs will not appear in the published article. *Only*  the individual author can complete the verification step; PLOS staff *cannot*  verify ORCID iDs on behalf of authors.

We look forward to receiving your revised manuscript.

Kind regards,

Emma Campbell, Ph.D

Staff Editor

PLOS Global Public Health

**Journal Requirements:**

1. Please include a complete copy of PLOS’ questionnaire on inclusivity in global research in your revised manuscript. Our policy for research in this area aims to improve transparency in the reporting of research performed outside of researchers’ own country or community. The policy applies to researchers who have travelled to a different country to conduct research, research with Indigenous populations or their lands, and research on cultural artefacts. The questionnaire can also be requested at the journal’s discretion for any other submissions, even if these conditions are not met.  Please find more information on the policy and a link to download a blank copy of the questionnaire here: https://journals.plos.org/globalpublichealth/s/best-practices-in-research-reporting. Please upload a completed version of your questionnaire as Supporting Information when you resubmit your manuscript.

2. Please amend your detailed Financial Disclosure statement. This is published with the article. It must therefore be completed in full sentences and contain the exact wording you wish to be published.

i. Please clarify all sources of financial support for your study. List the grants, grant numbers, and organizations that funded your study, including funding received from your institution. Please note that suppliers of material support, including research materials, should be recognized in the Acknowledgements section rather than in the Financial Disclosure.

ii. State the initials, alongside each funding source, of each author to receive each grant. For example: "This work was supported by the National Institutes of Health (####### to AM; ###### to CJ) and the National Science Foundation (###### to AM)."

iii. State what role the funders took in the study. If the funders had no role in your study, please state: “The funders had no role in study design, data collection and analysis, decision to publish, or preparation of the manuscript.”

iv. If any authors received a salary from any of your funders, please state which authors and which funders.

3. In the online submission form, you indicated that “The manuscript provides full methodological details of the Targeted Health Facility Assessment (tHFA). Copies of the tHFA questionnaire modules and related materials can be provided upon reasonable request to the corresponding author.”.

All PLOS journals now require all data underlying the findings described in their manuscript to be freely available to other researchers, either

1. In a public repository,

2. Within the manuscript itself, or

3. Uploaded as supplementary information.

This policy applies to all data except where public deposition would breach compliance with the protocol approved by your research ethics board. If your data cannot be made publicly available for ethical or legal reasons (e.g., public availability would compromise patient privacy), please explain your reasons by return email and your exemption request will be escalated to the editor for approval. Your exemption request will be handled independently and will not hold up the peer review process, but will need to be resolved should your manuscript be accepted for publication. One of the Editorial team will then be in touch if there are any issues.

4. Please ensure that your Ethics Statement is available in its entirety at the beginning of your Methods section, under a subheading 'Ethics Statement'.

If the reviewer comments include a recommendation to cite specific previously published works, please review and evaluate these publications to determine whether they are relevant and should be cited. There is no requirement to cite these works unless the editor has indicated otherwise.

**Additional Editor Comments (if provided):**

[Note: HTML markup is below. Please do not edit.]

Reviewers' comments:

Reviewer's Responses to Questions

**Comments to the Author**

1. Does this manuscript meet PLOS Global Public Health’s publication criteria?

Reviewer #1: No

Reviewer #2: Yes

**********

2. Has the statistical analysis been performed appropriately and rigorously?

Reviewer #1: I don't know

Reviewer #2: N/A

**********

3. Have the authors made all data underlying the findings in their manuscript fully available (please refer to the Data Availability Statement at the start of the manuscript PDF file)?

The PLOS Data policy

Reviewer #1: No

Reviewer #2: Yes

**********

4. Is the manuscript presented in an intelligible fashion and written in standard English?

Reviewer #1: Yes

Reviewer #2: Yes

**********

**Reviewer #1** : A well-wriiten Ms which provides clear descriptions of the rationale and basis of the study. The use of established norms and standards is welcomed. While the 11 programme indicators are appropriately detailed, it would assist the reader to have the key information related to these indicators in a tabular format for ease of reference.

The sampling strategy is acceptable and appropriate. The details related to ensuring quality data collection is notable, including embedding and employing independent data quality assurance procedures.

While the MS is focused on descrbing the tFHA as a pragmatic and scalable approach to monitor integrated service delivery and people-centered quality of care, there are no formal results presented from this acivity. Nevertheless, it does an admirable job of describing the design and implementation protocol for measuring integrated people-centered care. What appears to be missing is an evaluation framework that will assist in capturing the functionality of the tFHA as a monitoring tool.

The Discussion section refers to findings not evident in the MS. For example, on Pp 19, lines 367-372, reference is made to the use of indicators and data collection methods as facilitating comparibility across settings while maintaining relevance to conuntry-specific systems is done so in the abstract. Similar statements about tHFA being an agile and scalable platform for integrated service delivery and people-centred quality of care in LMICS in the conclusion to the study cannot be verified without reference to any evaluation data.

**Reviewer #2:**  I congratulate the authors on a clearly written paper which tackles the fundamental challenge of assessing the ability of health facilities to provide patient-centered care. My most substantial comments concern Tables 1 and 3, and the remaining are minor suggestions.

Questionnaire development:

Table 1 is the heart of the paper and provides substantial information, but could benefit from some additional clarifications and perhaps an additional column or two. I think it would be useful to make explicit the method of data collection in a separate column. For example, KPIS1.1 is record review and KPIS1.2 is patient exit interview. The record review is included in the attributes of the first domain, but not in the second. If the authors consistently pull these out into a separate column, it would improve consistency of information and interpretability by the reader. That column could also include an indication of the quantity of data reviewed or collected. The authors state later in the paper the number of interviews conducted but I don’t think there was information on how many charts or records were reviewed for the quantitative indicators. In Table 1, or maybe in a separate table in SI, the authors could provide some information on how terms are defined. For example, the definitions of communication and responsiveness raise questions as the conception of these by patients may vary a bit from setting to setting and it would be helpful for the reader to be able to understand more about how these components are assessed. Similarly, terms which could be more explicitly defined in the table include: 1) "supportive supervision" under KPIS2 raises questions about supervision by whom and of whom. Being as detailed as possible with titles will help the reader understand the importance of the work; 2) "no stock outs" under KPIS5.

In the methodology section, line 115, please make explicit how you are defining health facilities. You have the detailed definition later in the paper, but defining it earlier on will help guide the reader.

Line 203 is missing a period.

In the section beginning on line 213 (7. Programmatic Indicator 3: Health Sector Planning and Governance for Integrated People-Centred Services (HSG)-1.1: Availability of Clinical Guidelines), the authors discuss the review of availability of clinical guidelines. This was the first metric where I paused to consider how good the evidence is on whether the indicator (clinical guidelines availability) is actually associated with the outcomes of interest (integrated, people-centered care). You may want to consider expanding the introduction a bit to include the literature in support of the metrics, or this may be separate work that you could pursue independent of this paper.

Sampling:

Table 3 and the text in lines 293-301 may be more easily interpreted as a figure or flow chart. Table 3, if it remains, could use clarification in the column headings and information on how the sampling strategy aligns with the overall sample size. For example, the word Domains also appears in Table 1, and they have different meanings across the table, but the word is ambiguous and difficult to interpret without careful reading of the accompanying text.

Data collection and quality assurance:

In Table 4, you may want to add one additional column to indicate the number of days of data collection. If the point in Table 4 is mainly to convey the overlap in data collection periods, some kind of figure like a gantt chart may do that more clearly with one row per data collection activity (the current table rows).

Table 5 would be more interpretable as 3 columns without row markings as I think I should read it up and down, but not across. If the rows are useful, an initial column could give the row topic area to help guide the reader.

Discussion:

Line 385 discusses how this is a cost-efficient data collection tool. Do you envision this as being something that district health officers could do in the future or would this always sit with something like a central MOH team or the department of statistics? It would be interesting to add a comment on the long term vision for uptake.

The paper talks repeatedly about patient-centered experience, but the methods section don't mention the inclusion of patients in the development of the tools. If they were involved, please be explicit. If there was no role of patients in the development, this should be noted in the limitations section and the authors could reflect on how this impacts the results.

**********

what does this mean?). If published, this will include your full peer review and any attached files.

**Do you want your identity to be public for this peer review?**  If you choose “no”, your identity will remain anonymous but your review may still be made public.

For information about this choice, including consent withdrawal, please see our Privacy Policy

Reviewer #1: **Yes:**  Arvin Bhana

Reviewer #2: No

**********

[NOTE: If reviewer comments were submitted as an attachment file, they will be attached to this email and accessible via the submission site. Please log into your account, locate the manuscript record, and check for the action link "View Attachments". If this link does not appear, there are no attachment files.]

**Figure Resubmissions:**

# Author comment: Author response to Decision Letter 1

**DOI:** 10.1371/journal.pgph.0005736.r002

1 Jun 2026

Attachment Submitted filename: Response to Reviewers.docx

# Peer review: Decision Letter 1

**DOI:** 10.1371/journal.pgph.0005736.r003

**Authors:** Alejandro Torrado Pacheco

30 Jun 2026

PGPH-D-25-03786R1

Design and implementation of a multi-country targeted health facility assessment to measure integrated, people-centered, quality of services

PLOS Global Public Health

Dear Dr. Gupta,

Thank you for submitting your manuscript to PLOS Global Public Health. After careful consideration, we feel that it has merit but does not fully meet PLOS Global Public Health’s publication criteria as it currently stands. Therefore, we invite you to submit a revised version of the manuscript that addresses the points raised during the review process.

Please address the comment by Reviewer 1, which is available below.

Please submit your revised manuscript by Jul 30 2026 11:59PM. If you will need more time than this to complete your revisions, please reply to this message or contact the journal office at globalpubhealth@plos.org. When you're ready to submit your revision, log on to https://www.editorialmanager.com/pgph/ and select the 'Submissions Needing Revision' folder to locate your manuscript file.

Please include the following items when submitting your revised manuscript:

- A letter that responds to each point raised by the editor and reviewer(s). You should upload this letter as a separate file labeled 'Response to Reviewers'.
- A marked-up copy of your manuscript that highlights changes made to the original version. You should upload this as a separate file labeled 'Revised Manuscript with Track Changes'.
- An unmarked version of your revised paper without tracked changes. You should upload this as a separate file labeled 'Manuscript'.

Guidelines for resubmitting your figure files are available below the reviewer comments at the end of this letter.

As the corresponding author, your ORCID iD is verified in the submission system and will appear in the published article. PLOS supports the use of ORCID, and we encourage all coauthors to register for an ORCID iD and use it as well. Please encourage your coauthors to verify their ORCID iD within the submission system before final acceptance, as unverified ORCID iDs will not appear in the published article. *Only*  the individual author can complete the verification step; PLOS staff *cannot*  verify ORCID iDs on behalf of authors.

We look forward to receiving your revised manuscript.

Kind regards,

Alejandro Torrado Pacheco, PhD

Staff Editor

PLOS Global Public Health

**Journal Requirements:**

If the reviewer comments include a recommendation to cite specific previously published works, please review and evaluate these publications to determine whether they are relevant and should be cited. There is no requirement to cite these works unless the editor has indicated otherwise.

Please review your reference list to ensure that it is complete and correct. If you have cited papers that have been retracted, please include the rationale for doing so in the manuscript text, or remove these references and replace them with relevant current references. Any changes to the reference list should be mentioned in the rebuttal letter that accompanies your revised manuscript. If you need to cite a retracted article, indicate the article’s retracted status in the References list and also include a citation and full reference for the retraction notice.

**Additional Editor Comments (if provided):**

[Note: HTML markup is below. Please do not edit.]

Reviewers' comments:

Reviewer's Responses to Questions

**Comments to the Author**

Reviewer #1: All comments have been addressed

**********

publication criteria?

Reviewer #1: Yes

**********

3. Has the statistical analysis been performed appropriately and rigorously?

Reviewer #1: N/A

**********

4. Have the authors made all data underlying the findings in their manuscript fully available (please refer to the Data Availability Statement at the start of the manuscript PDF file)?

The PLOS Data policy

Reviewer #1: Yes

**********

5. Is the manuscript presented in an intelligible fashion and written in standard English?

Reviewer #1: Yes

**********

**Reviewer #1:**  While the authors indicate that they have removed statements that reflect impact, the concluding statement states: "The tHFA is a pragmatic, agile, scalable platform to monitor integrated service delivery and people-centred quality of care in LMICs. With a focused set 456 of process- and outcome-oriented measures, inclusion of CHWs, and alignment with the Global Fund Strategy (2023–2028), it is designed to supports 457 decision-making, programme monitoring, and health-system strengthening."

This statement does not appear to be a hypothetical statement, as you cannot describe this outcome without reference to a formal evaluation of the framework, unless the authors are referring to other literature where an evaluation framework was employed!

**********

what does this mean?). If published, this will include your full peer review and any attached files.

**Do you want your identity to be public for this peer review?**  If you choose “no”, your identity will remain anonymous but your review may still be made public.

For information about this choice, including consent withdrawal, please see our Privacy Policy

Reviewer #1: **Yes:**  Arvin Bhana

**********

[NOTE: If reviewer comments were submitted as an attachment file, they will be attached to this email and accessible via the submission site. Please log into your account, locate the manuscript record, and check for the action link "View Attachments". If this link does not appear, there are no attachment files.]

**Figure Resubmissions:**

# Author comment: Author response to Decision Letter 2

**DOI:** 10.1371/journal.pgph.0005736.r004

5 Jul 2026

Attachment Submitted filename: Response to reviewers_July2026.docx

# Editor report: Decision Letter 2

**DOI:** 10.1371/journal.pgph.0005736.r005

**Authors:** Julia Robinson

8 Jul 2026

Design and implementation of a multi-country targeted health facility assessment to measure integrated, people-centered, quality of services

PGPH-D-25-03786R2

Dear Dr. Gupta,

We are pleased to inform you that your manuscript 'Design and implementation of a multi-country targeted health facility assessment to measure integrated, people-centered, quality of services' has been provisionally accepted for publication in PLOS Global Public Health.

Before your manuscript can be formally accepted you will need to complete some formatting changes, which you will receive in a follow up email. A member of our team will be in touch with a set of requests.

Please note that your manuscript will not be scheduled for publication until you have made the required changes, so a swift response is appreciated.

IMPORTANT: The editorial review process is now complete. PLOS will only permit corrections to spelling, formatting or significant scientific errors from this point onwards. Requests for major changes, or any which affect the scientific understanding of your work, will cause delays to the publication date of your manuscript.

If your institution or institutions have a press office, please notify them about your upcoming paper to help maximize its impact. If they'll be preparing press materials, please inform our press team as soon as possible -- no later than 48 hours after receiving the formal acceptance. Your manuscript will remain under strict press embargo until 2 pm Eastern Time on the date of publication. For more information, please contact globalpubhealth@plos.org.

Thank you again for supporting Open Access publishing; we are looking forward to publishing your work in PLOS Global Public Health.

Best regards,

Julia Robinson

Executive Editor

PLOS Global Public Health

***********************************************************

Reviewer Comments (if any, and for reference):
